# Combined use of cutinase and high-resolution mass-spectrometry to query the molecular architecture of cutin

**DOI:** 10.1186/s13007-018-0384-6

**Published:** 2018-12-26

**Authors:** Rupam Kumar Bhunia, Lucas J. Showman, Adarsh Jose, Basil J. Nikolau

**Affiliations:** 1 Department of Biochemistry, Biophysics, and Molecular Biology, Iowa State University, 3254 Molecular Biology Building, 2437 Pammel Drive, Ames, IA 50011 USA; 2 Center for Metabolic Biology, Iowa State University, Ames, IA 50011 USA; 3 W. M. Keck Metabolomics Research Laboratory, Iowa State University, Ames, IA 50011 USA; 4 Plant Tissue Culture and Genetic Engineering, National Agri-Food Biotechnology Institute (NABI), Mohali, Punjab 140306 India

**Keywords:** Cutin, Cutinase, Liquid chromatography–quadrupole time-of-flight (Q-TOF) MS, GC–MS, MALDI-MS imaging, *Malus pumila*

## Abstract

**Background:**

Cutin is a complex, highly cross-linked polyester consisting of hydroxylated and epoxidated acyl lipid monomers. Because of the complexity of the polymer it has been difficult to define the chemical architecture of the polymer, which has further limited the ability to identify the catalytic components that assemble the polymer. Analogous to methods that define the structure of oligosaccharides, we demonstrate a strategy that utilizes cutinase to generate cutin subfragments consisting of up to four monomeric units, whose structure and spatial distribution in the polymer is revealed by high-resolution mass spectrometry. Moreover, the application of mass-spectrometric fragmentation and labelling of the end of the oligomers, one is able to define the order of monomers in the oligomer. The systematic application of this strategy can greatly facilitate understanding the chemical architecture of this complex polymer.

**Results:**

The chemical architecture of plant cutin is dissected by coupling an enzymatic system that deconstructs the polymer into subfragments consisting of dimers, trimers and tetramers of cutin monomers, with group-specific labeling and mass spectrometry. These subfragments can be generated with one of over 1200 of cutinases identified from diverse biological sources. The parallel chemical labeling of the polymer with dansyl, alkyl or *p*-dimethylaminophenacyl reagents can identify the chemical distribution of non-esterified hydroxyl- and carboxyl-groups among the monomers. This combined strategy is applied to cutin isolated from with apple fruit skins, and a combination of gas chromatography–mass spectrometry (GC–MS) and liquid chromatography–quadrupole time-of-flight (Q-TOF) MS is used to determine the order of the monomers in the cutinase-generated subfragments. Finally, we demonstrate the use of matrix-assisted laser desorption-ionization-MS to determine the spatial distribution of the cutinase-generated subfragments.

**Conclusion:**

Our experimental results demonstrate an advancement to overcome the current limitations in identifying cutin oligomeric structure and allows one to more efficiently address new biological questions about cutin biosynthesis. We submit that the systematic application of these methods will enable the construction of more accurate architectural models of cutin, which is a prerequisite to identifying cutin-biosynthetic components.

**Electronic supplementary material:**

The online version of this article (10.1186/s13007-018-0384-6) contains supplementary material, which is available to authorized users.

## Background

The aerial epidermis of terrestrial plants is covered by a hydrophobic structural support matrix, known as the cuticle, which functions primarily as a protective water-barrier, and further provides protection from pathogens and irradiation damage. The cuticle is composed of two components, the non-extractable cutin and the solvent extractable cuticular lipids, historically also called cuticular waxes [1, 2]. Cutin is a non-linear, cross-linked polymer composed of fatty acids (FAs), hydroxy-FAs, epoxy-FAs, diacids, diols and triols, which are cross-linked primarily with covalent ester bonds [3, 4]. Despite extensive knowledge of the monomeric constituents of cutin [5, 6], the complexities of its polymeric structure and its inter-connections with other cellular components has hindered the ability to understand how cutin is assembled and how it serves its biological function [7, 8].

Cutin is one of the most energy-dense type of molecule that biology can produce, and thus represents a highly efficient chemical organization for the chemical storage of energy. Therefore, its chemical structure could serve as a template for efficiently storing of carbon-based energy that is initially harnessed from solar energy. Although advancements have been made in identifying the architecture of cutin using solid state NMR and mass spectrometric analysis of soluble cutin oligomers following acid- or base-catalyzed depolymerization of cutin [9, 10], a comprehensive higher order structure of this important polymer is still unknown.

In this study, we evaluated the use of hydrolytic enzymes in depolymerizing cutin and beginning to decipher the higher order organization and structure of the polymer. The strategy is similar to that previously used in the characterization of complex cell wall polysaccharide structure by using specific hydrolases to depolymerize oligosaccharides and determine the monosaccharide composition [11, 12], primarily using enzymes that hydrolyze the ester-bonds of cutin and generating smaller oligomers whose chemical structures can be subsequently determined by high resolution mass spectrometry. Microorganisms have developed a large number of hydrolytic enzymes, which are used to penetrate the outer plant barrier and enter the plant interior. These include a large number of cutinases that hydrolyze the ester bonds of cutin [13, 14], allowing the germinating fungal spore access to the plant interior. Cutinases are a member of the α/β class of serine esterases that hydrolyze ester bonds [15, 16], and are known to depolymerize intact cutin to its residual cutin monomers [17].

In the present study, we used recombinant cutinase enzymes to generate cutin subfragments from isolated apple cutin preparations. Exemplary monomers, dimers, trimers and tetramers of the cutin monomers were characterized by a combination of gas chromatography–mass spectrometry (GC–MS) and liquid chromatography–mass spectrometry (LC–MS). Using a high mass resolution Quadrupole Time-of-Flight (Q-TOF) mass spectrometer enabled the identification of the composition and chemical order of the monomers that constitute the cutinase-generated oligomers. The sequential application of cutinase treatment and MALDI-based mass spectrometric imaging enabled the determination of the spatial mapping of the cutin subfragments on plant tissue surfaces. Additionally, chemical modification of the isolated cutin identified the chemical nature of the exposed non-esterified hydroxyl and carboxyl groups that constitute the cutin polymer. Ultimately, the combined application of these strategies offers a path to determining a comprehensive understanding of the molecular architecture of the cutin polymer.

## Results

### Bioinformatic identification of cutinases from different phylogenetic sources

**Fig. 1:**
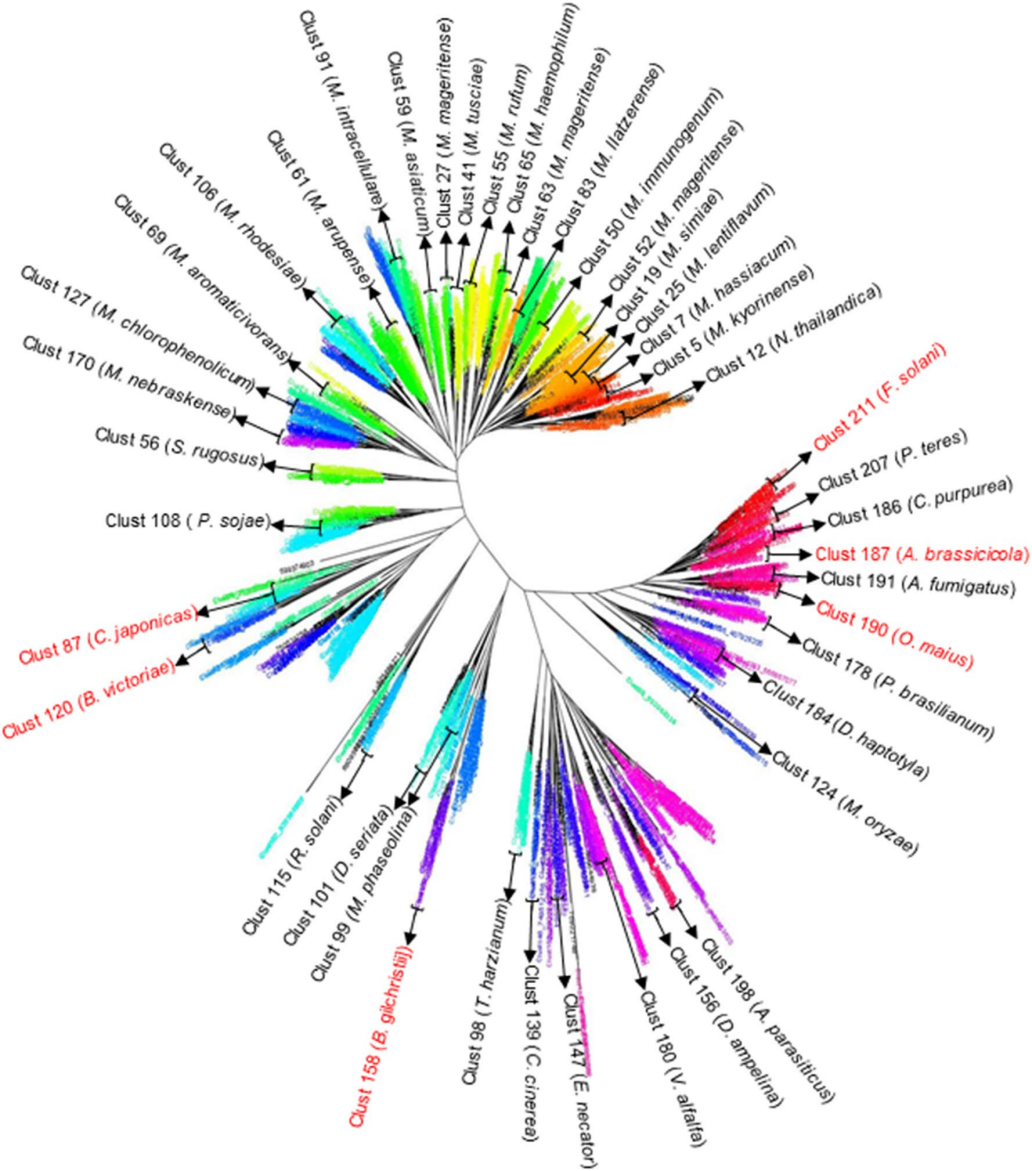
Hierarchical phylogenetic analysis of 1243 cutinase sequences clustered in 211 different clades (color coded). The arrows indicate the 43 cutinases selected from each clade for recombinant expression in *E. coli*

A total of 15,848 non-redundant amino acid sequences were computationally identified after performing BLAST analysis at the NCBI database [18] using *Fusarium solani* cutinase protein as a query sequence (GenBank: AAA33334). All 15,848 amino acid sequences were analyzed with the CD-HIT tool [19, 20], and sequences of between 210 and 250 amino acids were chosen for further analysis. The resulting 1243 sequences (Additional file 1: Table S1) were subjected to phylogenetic analysis using the Cluster Picker tool [21], which grouped these sequences into 211 clusters (Fig. 1). Forty-three cutinases were selected for additional molecular studies, and these broadly represented the 211 clusters. The ORFs encoding the selected 43 cutinases were codon optimized for *E. coli* expression and these DNA sequences were chemically synthesized.

### Expression, purification and biochemical characterization of cutinases from different phylogenetic sources

The codon optimized ORFs were expressed in *E. coli* as His-tagged proteins, and 6 cutinase proteins were successfully recovered. A modified sarkosyl-based protein extraction-purification method [22] was used to isolate the cutinase proteins from cell extracts, and the cutinase enzymes were purified using Ni-column affinity chromatography. The purified cutinases were determined to be greater than 95% homogeneous by SDS-PAGE (Additional file 2: Fig. S1), and they migrated within the expected molecular weight range of between 24 and 30 kDa.

The catalytic activities of the purified cutinases were characterized using artificial *p*-nitrophenyl ester substrates. The *F. solani* and *Alternaria brassicicola* cutinases showed activity with these substrates. Using *p*-nitrophenyl butyrate (*p*-NPB) as the substrate *F. solani* enzyme exhibits a *Km* of 1.26 ± 0.06 mM and *k*_*cat*_ of 25 ± 0.85 s^−1^. The *A. brassicicola* cutinase displays similar *Michaelis*–*Menten* kinetic constants, with a *Km* of 0.87 ± 0.05 mM and *k*_*cat*_ of 12 ± 0.80 s^−1^ (Additional file 3: Fig. S2a). We also explored the substrate preferences of these two enzymes by using *p*-NP esters of FAs of different acyl chain length, ranging from C2 to C18 (Additional file 3: Fig. S2b). Both the cutinases show similar substrate preferences, with highest hydrolytic activity with *p*-nitrophenyl esters of medium acyl chain lengths (8–14 carbons); hydrolytic activity with esters of shorter or longer acyl chain lengths is reduced by more than half from that displayed with the optimal substrate.

### Identification of apple cutin monomers

**Fig. 2:**
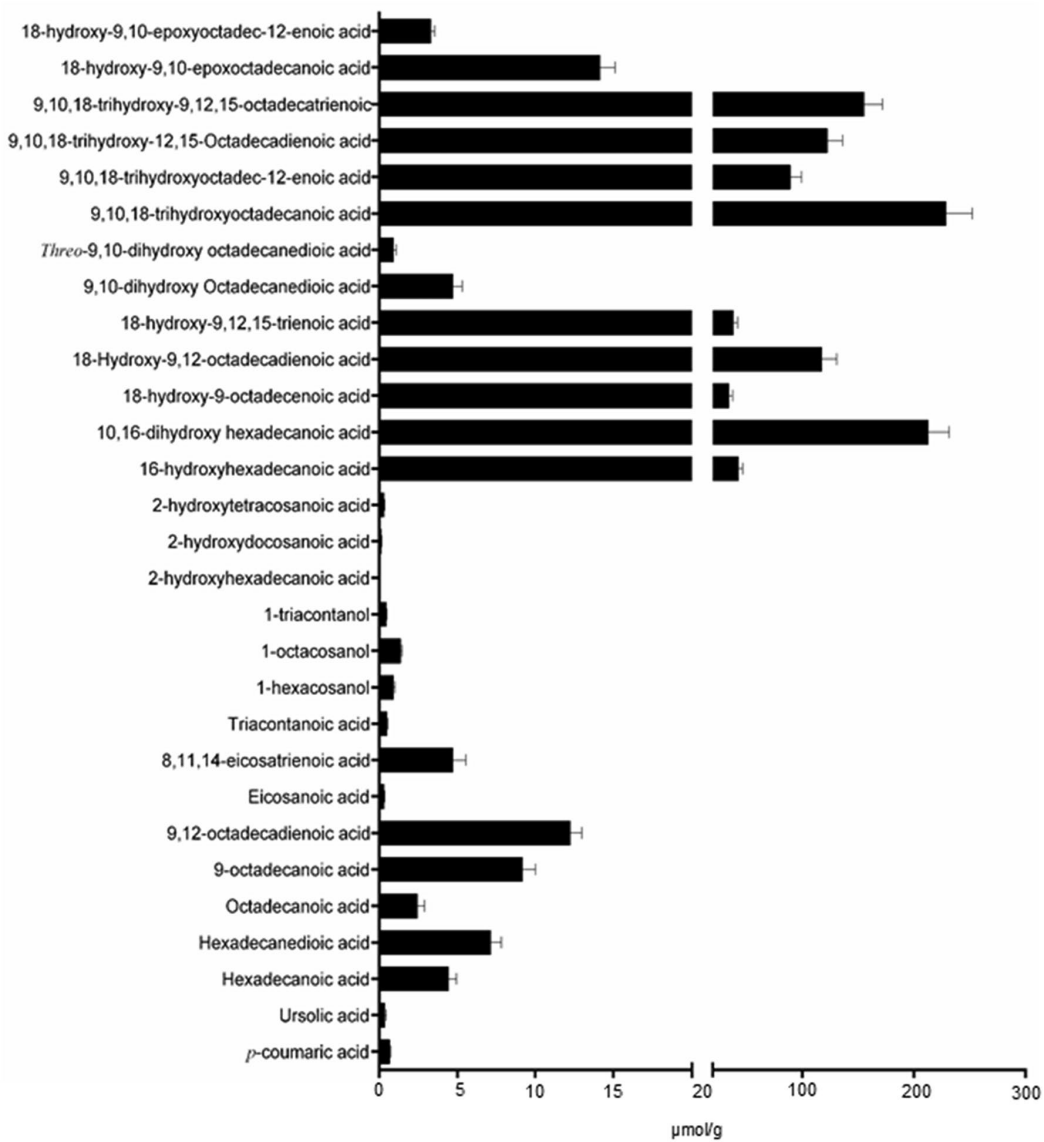
Quantitative analysis of apple cutin monomers recovered following acid-hydrolysis. Error bars indicate the SD values of three determinations

Monomers released by acid-catalyzed methanolysis of isolated apple skin cutin were identified and quantified by GC-MS analysis. These monomers are primarily isomers of monohydroxy-, dihydroxy- and trihydroxy-saturated and unsaturated, 16- and 18-carbon FAs (Fig. 2). The six most abundant of these monomers, which account for ~ 80% of the isolated cutin monomers, are 9,10,18-trihydroxyoctadecanoic acid (9,10,18-trihydroxy-18:0), 10,16-dihydroxy hexadecanoic acid (10,16-dihydroxy-16:0), 9,10,18-trihydroxy-9,12,15-octadecatrienoic acid (9,10,18-trihydroxy-18:3), 9,10,18-trihydroxy-9,12-octadecadienoic acid (9,10,18-trihydroxy-18:2), 18-hydroxy-9,12-octadecadienoic acid (18-hydroxy-18:2), 9,10,18-trihydroxyoctadec-12-enoic acid (9,10,18-trihydroxy-18:1).

**Fig. 3:**
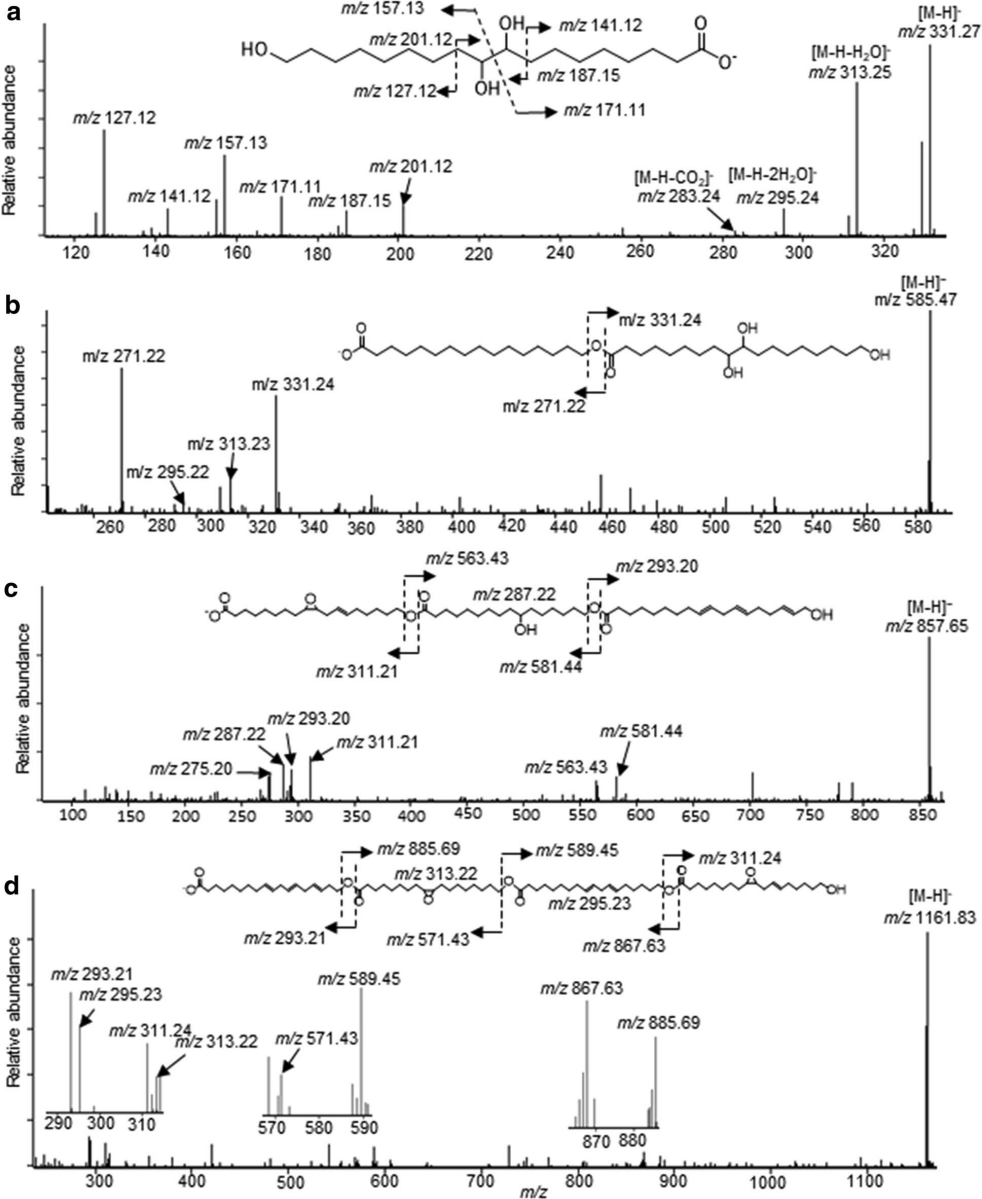
Exemplary MS/MS characterization of cutinase-generated cutin hydrolysis products. **a** Identification of the monomer, 9,10,18-trihydroxyoctadecanoic acid (9,10,18-trihdroxy-18:0). **b** Identification of the dimer, 16-hydroxy-16:0/9,10,18-trihydroxy-18:0 (*m/z* 585.47). **c** Identification of the trimer, 18-hydroxy-9,10-epoxy-18:1/10,16-dihydroxy-16:0/18-hydroxy-18:3 (*m/z* 857.65). **d** Identification of the tetramer, 18-hydroxy-18:3/18-hydroxy-9,10-epoxy-18:0/18-hydroxy-18:2/18-hydroxy-9,10-epoxy-18:0 (*m/z* 1161.83)

Enzymatic deconstruction products of the isolated cutin preparations were identified by a combination of LC–MS and LC–MS/MS analyses. Initially these experiments characterized cutin monomers generated by the near-complete hydrolysis of cutin with the *F. solani* cutinase (Additional file 4: Fig. S3 and Fig. 3a). Exemplary LC–MS/MS spectrum of an isomer of trihydroxy-C18 acid is shown in Fig. 3a. The interpretation of MS/MS fragment ions of *m/z* values: 313.25, 295.24, 283.24, 201.12, 187.15, 171.11, 157.13, 141.12 and 127.12 is consistent with the presence of 2 mid-chain hydroxyl groups at the C_9_ and C_10_ position, and a 3rd hydroxyl group at the ω-end of the FA; thereby identifying this monomer as 9,10,18-trihydroxyoctadecanoic acid (9,10,18-trihydroxy-18:0; *m/z* 331.27). Similarly, one can rationalize the identification of 9,10,18-trihydroxy-18:1 (*m/z* 329.25) (Additional file 4: Fig. S3b), 9,10,18-trihydroxy-18:2 (*m/z* 327.22) (Additional file 4: Fig. S3c) and 9,10,18-trihydroxy-18:3 (*m/z* 325.20). The analogous MS and MS/MS strategy was used to characterize additional dihydroxy-FAs, and these include 10,16-dihydroxy-C16:0 (*m/z* 287.24) (Additional file 4: Fig. S3d and S3e), 10,16-dihydroxy-16:1 (*m/z* 285.24), and the monohydroxy-FAs: 18-hydroxy-18:1 (*m/z* 297.24), 18-hydroxy-C18:2 (*m/z* 295.23), and 18-hydroxy-18:3 (*m/z* 293.23) (Additional file 4: Fig. S3f and S3g).

Similar LC–MS and LC–MS/MS experiments identified three epoxy-FAs (Additional file 4: Fig. S3h–k), and these were predominantly of 18-carbon chain-length and were also hydroxylated at the ω-end of the alkyl-chain. Specifically, 18-hydroxy-9,10-epoxystearic acid (18-OH-9,10-epoxy-18:0, *m/z* 313.25) (Additional file 4: Fig. S3i) generated a set of prominent MS/MS fragmentation ions (*m/z* 295.24, 277.24, 267.25, 171.11, 157.12, 155.12, 141.12 and 127.12), which are consistent with the cleavage reactions illustrated on the chemical structure inserted in Additional file 4: Fig. S3i. Cleavage of C_8_–C_9_ and C_10_–C_11_ bonds, yields fragment of *m/z* values of 141.12 and 127.12, respectively. The fragment ions at *m/z* 157.12, 155.12 and 171.11, are diagnostic of the epoxy-group between the C_9_ and C_10_ positions. Analogous LC–MS and LC–MS/MS experiments were used to deduce the occurrence of 18-hydroxy-9,10-epoxy-18:1 (Additional file 4: Fig. S3j) and 18-hydroxy-9,10-epoxy-18:2 (Additional file 4: Fig. S3k).

### Identification of cutin oligomers from partial hydrolysis of apple cutin with cutinase

Having identified that the cutin monomers are in the *m/z* range of 164–331, we surmised that LC–MS analysis of dimers, trimers and tetramers that would be generated by partial cutinase hydrolysis of cutin will present ions with *m/z* values in the range of 328–662, 492–993 and 656–1324, respectively. Moreover, because these products were analyzed in negative [M–H]^−^ ion mode, rather than positive [M–H]^+^ ion mode, the oligomers that were detected would be expected to carry at least one free carboxyl group. Although positive ion mode was used in preliminary studies, it proved less sensitive than negative ion mode, which may be an indication that in the overall cutin structure dimers, trimers and tetramers of hydroxy-FAs without any free carboxyl groups are rare.

Figure 3b shows an example of a cutin dimer, and the MS spectral analysis that was used to deduce its chemical structure. LC–MS analysis indicates that it exhibits a molecular ion of *m/z* 585.47 [M−H]^−^, and MS/MS fragmentation of this ion results in two major ions of *m/z* 271.22 and 331.24. The dehydration products of the 331.24 ion, generates ions with *m/z* 313.23 (331.24-H_2_O) and 295.22 (331.24-2H_2_O), which reflects the presence of a hydroxyl group on the monomers that constitutes the dimer. Based on this fragmentation pattern, and the characterization of cutin monomers presented in Additional file 4: Fig. S3, leads to the identification of this dimer as consisting of the two monomers, 16-hydroxy-16:0 (*m/z* 271.22) and 9,10,18-trihydroxy-18:0 (*m/z* 331.24), where the hydroxyl-group at the ω-carbon of the former monomer is acylated by the latter acid.

Additional file 5: Fig. S4a shows the characterization of another dimer with [M–H]^−^ molecular ion *m/z* 579.42, consisting of the monomer 10,16-dihydroxy-16:1 (*m/z* 285.20) acylated by 18-hydroxy-9,10-epoxy-18:1 (*m/z* 311.22). The neutral loss of H_2_O is evident of the 10,16-dihydroxy-16:1 moiety (*m/z* 285.20-H_2_O), originating from the non-esterified primary (at the ω-carbon) or secondary (at the 10th-carbon) hydroxyl group, generating the product ion *m/z* 267.19. Similarly, Additional file 5: Fig. S4b and S4c identify additional dimers between moieties of 18-hydroxy-18:3 (*m/z* 293.21), 18-hydroxy-9,10-epoxy-18:0 (*m/z* 313.23) and 18-hydroxy-9,10-epoxy-18:1 (*m/z* 311.22).

Cutinase hydrolysis subfragments consisting of three monomers were identified in the total ion chromatogram at between *m/z* values of 492–993. MS/MS fragmentation experiments identified the constituent cutin monomers of each trimer. Namely, each trimer fragmented to generate two respective dimers and the three monomers. Figure 3c shows a typical example of the characterization of a trimer with molecular ion at [M–H]^−^ *m/z* 857.65. MS/MS fragmentation yields two major dimer daughter ions, *m/z* 581.44 and 563.43, and monomer daughter ions with *m/z* 311.21, 287.22 and 293.20. The latter 3 correspond to 18-hydroxy-9,10-epoxy-18:1, 10,16-dihydroxy-16:0 and 18-hydroxy-18:3. The dimer consisting of 18-hydroxy-9,10-epoxy-18:1 and 10,16-dihydroxy-16:0 was identified based on the fact that it generates ions of, *m/z* 311.21 + 287.22, with the neutral loss of H_2_O yielding the identified ion, *m/z* 581.44. Similarly, a dimer of 10,16-dihydroxy-16:0 and 18-hydroxy-18:3 (*m/z* = 287.22 + 293.20), with neutral loss of H_2_O would yield the identified ion, *m/z* 563.43. Because the *m/z* 287.22 ion is common to both dimer daughter fragments, the 10,16-dihydroxy-16:0 moiety must occupy the central position of the trimer. Consistent with the deduced structure of the trimer shown in Fig. 3c, the neutral loss of H_2_O from the primary hydroxyl group of 18-hydroxy-18:3 (*m/z* 293.20) yields the *m/z* 275.20 ion.

Similar deductive strategies were used to identify the structures of the trimers with molecular ions [M–H]^−^ of *m/z* 859.66, 841.65 and 825.66 (Additional file 6: Fig. S5a, S5b and S5c). These three trimers are composed of monomeric units of (1) 18-hydroxy-18:3 (*m/z* 293.21), 10,16-dihydroxy-16:0 (*m/z* 287.22) and 18-hydroxy-9,10-epoxy-18:0 (*m/z* 313.23); (2) 18-hydroxy-18:3 (*m/z* 293.21), 18-hydroxy-18:2 (*m/z* 295.23) and 10,16-dihydroxy-16:0 (*m/z* 287.21); and (3) 18-hydroxy-18:3 (*m/z* 293.21), 18-hydroxy-18:2 (*m/z* 295.22) and 16-hydroxy-16:0 (*m/z* 271.22), respectively.

Cutinase-generated hydrolysis products consisting of four monomeric units were identified in the LC–MS total ion chromatogram in the *m/z* range of 656–1324. As with the analyses of the dimers and trimers, Fig. 3d shows an exemplary MS/MS characterization of a tetramer with a [M–H]^−^ molecular ion of *m/z* 1161.83. The daughter fragments were identified as two trimers, two dimers and the four monomer fragments. The monomer fragments identified that this tetramer consists of 18-hydroxy-18:3 (*m/z* 293.21), 18-hydroxy-9,10-epoxy-18:0 (*m/z* 313.22), 18-hydroxy-18:2 (*m/z* 295.23), and 18-hydroxy-9,10-epoxy-18:1 (*m/z* 311.24) moieties. The order of these moieties was deduced from the *m/z* values of the two daughter trimers (*m/z* 867.63 and 885.69) and the two daughter dimers (*m/z* 571.43 and 589.45). The trimers arise because of the fragmentation of 18-hydroxy-18:3 (*m/z* 293.21-H_2_O = 275.20) or of 18-hydroxy-9,10-epoxy-18:1 (*m/z* 311.24-H_2_O = 293.21), which establishes that these monomers are at the terminal ends of the tetramer. Additional structural information was gathered from the recovered dimeric ions of *m/z* 571.43 and 589.45. The *m/z* 571.43 ion is due to neutral loss of water from the terminal FA and consists of 18-hydroxy-9,10-epoxy-18:0 (*m/z* 313.22) acylated by 18-hydroxy-18:3 (*m/z* 293.21). The second dimer (*m/z* 589.45) consists of 18-hydroxy-9,10-epoxy-18:1 (*m/z* 311.24) acylated by 18-hydroxy-18:2 (*m/z* 295.23). Integrating these MS/MS data enables the structural deduction of this tetramer as 18-hydroxy-9,10-epoxy-18:1, acylated by 18-hydroxy-18:2, which is acylated by 18-hydroxy-9,10-epoxy-18:0, which is further acylated by 18-hydroxy-18:3. Similarly, Additional file 7: Fig. S6a and S6b shows [M–H]^−^ molecular ions *m/z* 1125.92 and 1143.88 corresponding to tetramers, formed by the esterification of two moieties of 18-hydroxy-18:2 (*m/z* 295.22) and two moieties of 18-hydroxy-18:3 (*m/z* 293.21), and two moieties of 18-hydroxy-18:3 (*m/z* 293.21) with one moiety of 18-hydroxy-18:2 (*m/z* 295.24) and 18-hydroxy-9,10-epoxy-18:0 (*m/z* 313.24), respectively.

### Labeling of the free hydroxyl and carboxyl groups in intact cutin

**Fig. 4:**
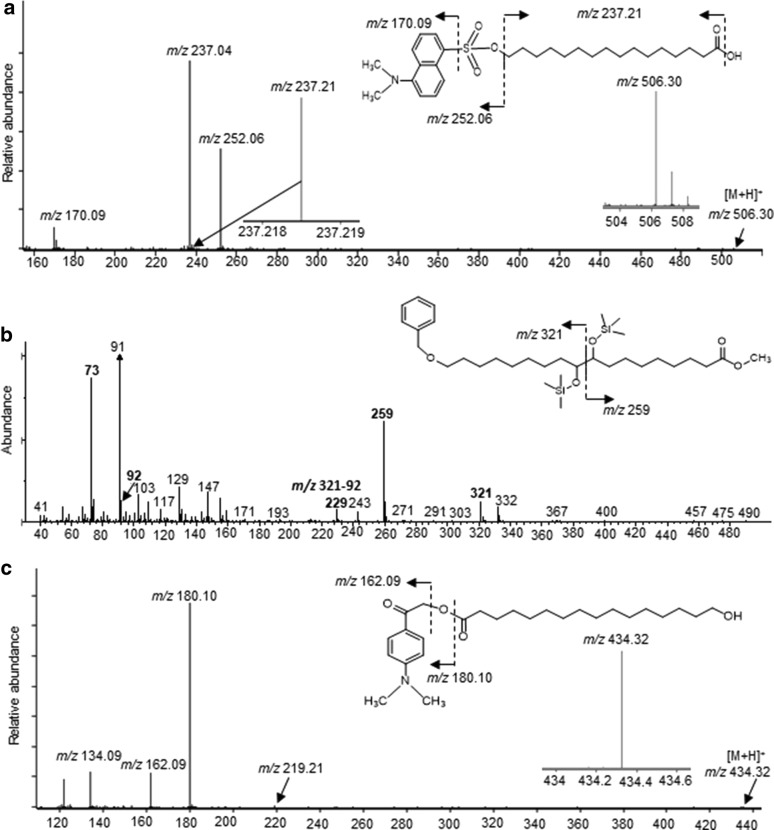
Chemical derivatization of non-esterified-hydroxyl- and carboxyl groups in intact cutin. **a** LC–MS/MS characterization of a cutinase hydrolysis product obtained from dansylated cutin; identification of the dansyl-derivative of 16-hydroxy-16:0 (*m/z* 506.30). **b** GC/MS characterization of a cutinase hydrolysis product obtained from benzyl-*O*-alkylated cutin; identification of 18-*O*-benzyl-derivative of 9,10,18-trihydroxy-18:0. **c** LC–MS/MS characterization of a cutinase hydrolysis product obtained from DmPA-derivatized cutin; identification of DmPA-derivatized 16-hydroxy-16:0 (*m/z* 434.38)

The non-esterified and thus free hydroxyl groups present in the cutin polymer were chemically labeled by either dansylation or benzyl-*O*-alkylation reactions. Hydrolysis of the derivatized cutin and LC-MS/MS or GC–MS analysis, identified those moieties that carried terminal, free hydroxyl groups in the intact cutin polymer. In positive ion mode, an exemplary cutinase-generated, dansylated product of 16-hydroxy-16:0 (*m/z* 506.30) was identified via the characteristic MS/MS fragment ions of *m/z* 252.06, 171.09 and 237.21 (Fig. 4a). Similarly, Additional file 8: Fig. S7a and S7b shows the dansylated products of 18-hydroxy-18:2 (*m/z* 530.29) and 18-hydroxy-9,10-epoxy-18:0 (*m/z* 548.32).

**Fig. 5:**
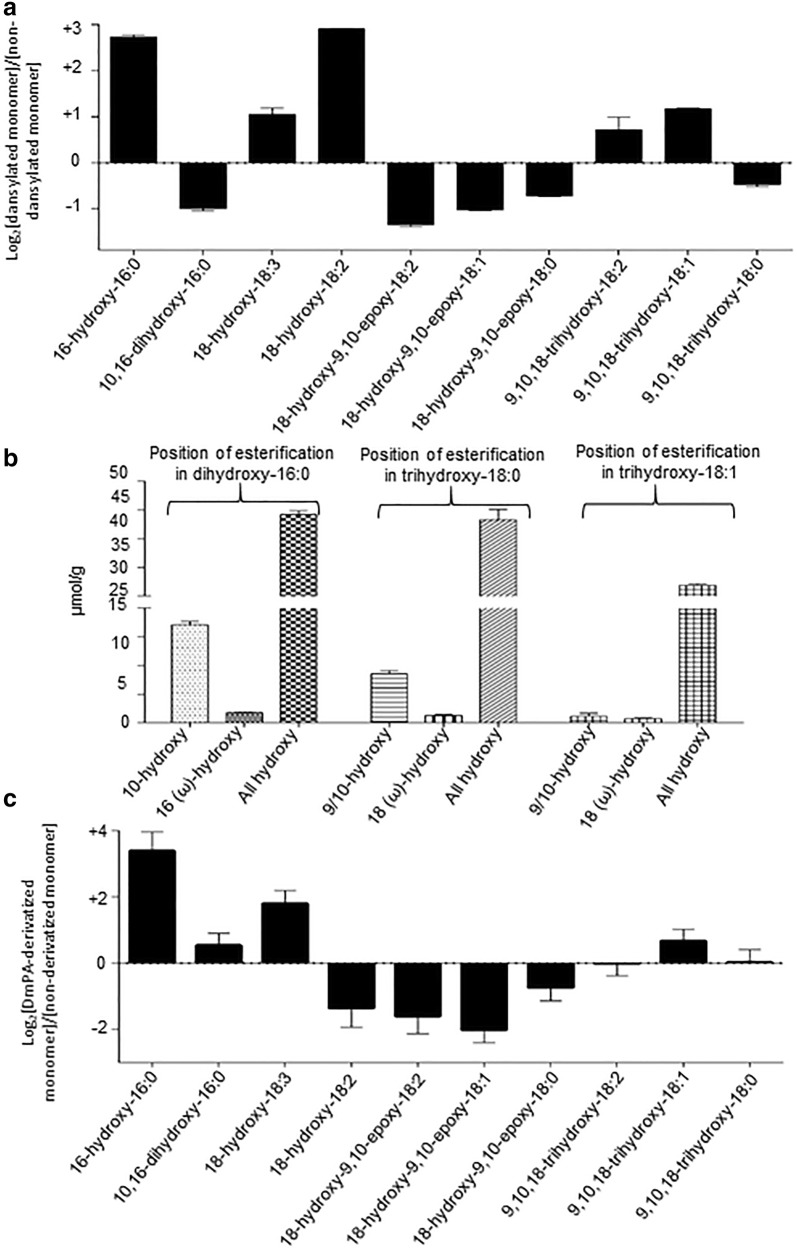
**a** Relative ratio of dansylated and non-dansylated cutin-monomers recovered from cutinase hydrolysis of dansylated intact cutin. **b** Recovery of alkylated and nonalkylated cutin-monomers generated by the hydrolysis of benzyl-*O*-alkylated intact cutin. **c** Relative ratio of DmPA-derivatized and non-derivatized cutin-monomers recovered from hydrolysis of DmPA-treated intact cutin. The calculation of the standard error in panels **a**, **b** is described in the Materials and methods

The parallel negative ion mode analysis of these samples, identified those hydroxy-FAs that were not dansylated, because these hydroxyl-groups were involved in the ester bonds that constitute the intact cutin polymer. Although all expected hydroxy-FAs were dansylated, the log-transformed relative ratio of the dansylated and non-dansylated hydroxy-FAs (Fig. 5a) indicate that the hydroxyl group of 16-hydroxy-16:0 and 18-hydroxy-18:2 predominate as the terminal moieties in the intact cutin polymer, and these hydroxyl-groups tend not to be involved in the ester-bonds that form the cutin polymer.

Analogous experiments were conducted with benzyl-*O*-alkylated cutin, and acid-hydrolysis of the modified polymer was followed by silylation, and the mixture was analyzed by GC–MS. This strategy identified the hydroxyl-groups of 10,16-dihydroxy-16:0, 9,10,18-trihydroxy-18:0 and 9,10,18-trihydroxy-18:1 FAs that are not involved in cross-esterification in the intact polymer and are were thus available for alkylation. Figure 4b shows the exemplary mass spectrum of the alkylated 9,10,18-trihydroxy-18:0 FA recovered from these analyses, in which the benzyl group occurs at the primary hydroxyl group (ω-end) of the fatty acid. Additional file 9: Fig. S8 shows the other examples of non-alkylated and alkylated 10,16-dihydroxy-16:0 FAs and 9,10,18-trihydroxy-18:0 FA. Specifically, the alkylated FAs that were recovered were all modified with a single benzyl-ether group. Figure 5b presents quantitative data, which establishes that the vast majority (> 95%) of these hydroxy-FAs are not alkylated. Therefore, in the intact cutin polymer the hydroxyl groups on these FAs are involved in ester bond formation, indicating that these trihydroxy FAs are highly involved in cross-linking of the cutin polymer.

A similar strategy, using *p*-dimethylaminophenacyl (DmPA) bromide as the derivatizing reagent was used to label and identify the non-esterified carboxyl groups in the cutin polymer. MS/MS analysis of the cutinase-hydrolyzed derivatized polymer, identified the DmPA modified 16-hydroxy-16:0 (*m/z* 434.32) by the characteristic daughter ions of *m/z* 180.10 and 162.09 (Fig. 4c). Similarly, Additional file 10: Fig. S9a and S9b showing the DmPA modified products of 18-hydroxy-18:3 (*m/z* 458.32) and 9,10,18-trihydroxy-C18:1 (*m/z* 492.30). Similar to the interpretation of the dansyl- and benzyl-derivatized cutin, the analysis of the DmPA-derivatized cutin identified the carboxyl-groups of 16-hydroxy-C16:0 and 18-hydroxy-18:3 as the predominant moieties available for DmPA-derivatization (Fig. 5c), which implies that they are enriched as the terminal moieties in the intact cutin polymer.

### Imaging the spatial distribution of cutinase-generated cutin fragments

**Fig. 6:**
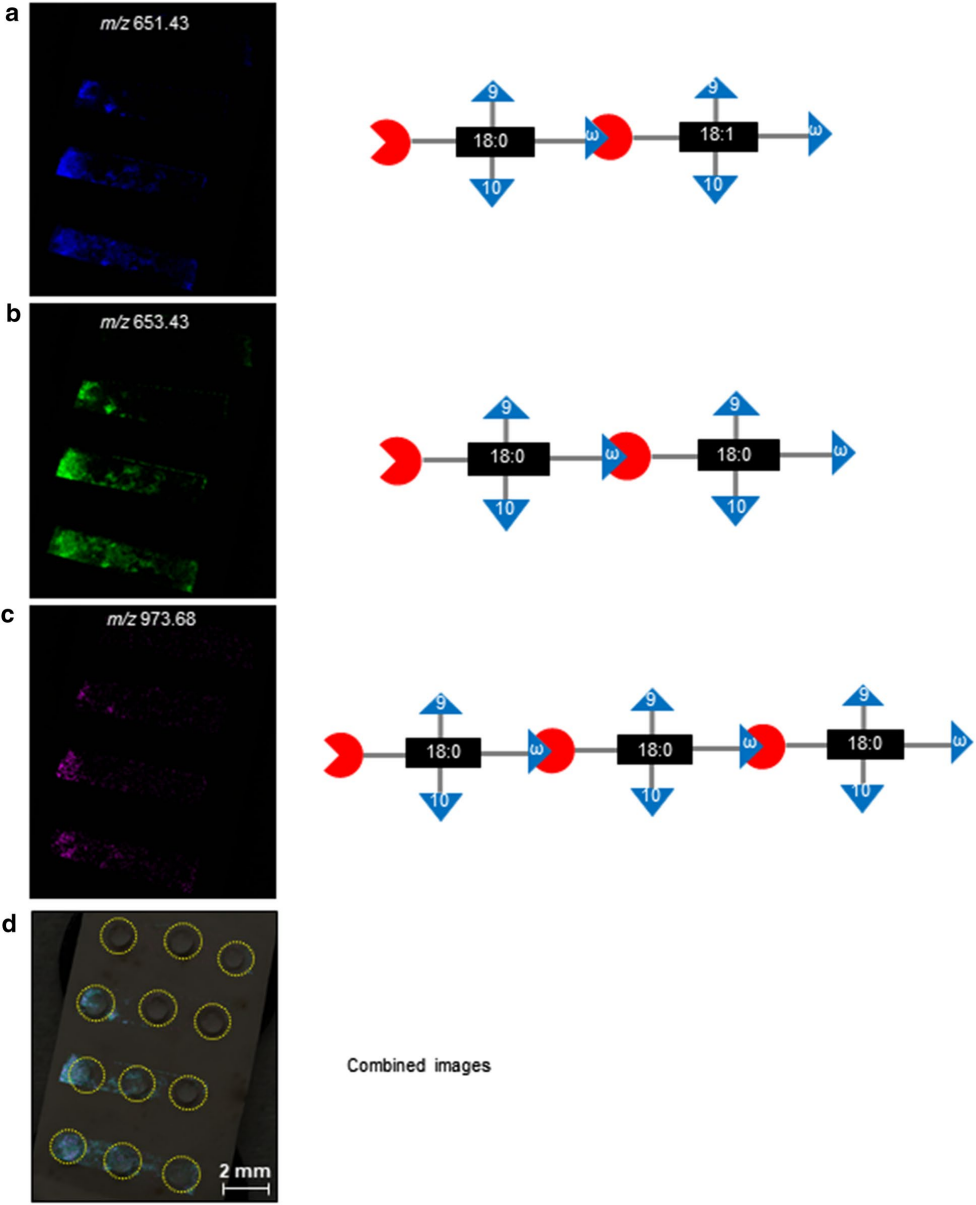
Spatial distribution of cutinase-generated cutin fragments visualized by MALDI-based mass spectrometry. False color intensity of the relative abundance of **a** 9,10,18-trihydroxy-18:0/9,10,18-trihydroxy-18:1 (*m/z* 651.43) dimer (blue); **b** 9,10,18-trihydroxy-18:0/9,10,18-trihydroxy-18:0 (*m/z* 653.43) dimer (green); **c** 9,10,18-trihydroxy-18:0/9,10,18-trihydroxy-18:0/9,10,18-trihydroxy-18:0 (*m/z* 973.68) trimer (purple). **d** The three molecular images shown in panels **a**–**c**, are overlayed on the visual image of the apple skin section with the cutinase containing droplets (highlighted by the yellow circles)

MALDI-based mass-spectrometric imaging [23] was applied to visualize the spatial distribution of cutinase-generated cutin fragments on the apple-skin surface. In these experiments apple peel preparations were initially exhaustively extracted with polar and non-polar solvents in order to remove non-polymeric metabolites from the preparations. The resulting de-lipidated peels were dried and placed on a MALDI-imaging plate. A dilution series of a cutinase preparation was spotted in adjoining positions of the peel and was allowed to hydrolyze the cutin for 1 h at room temperature. The cutinase reaction was terminated by *in vacuo* drying and following the application of lithium-doped 2,5-dihydroxybenzoic acid (Li-DHB) as the MALDI matrix, the spatial distribution of the digested products was determined by mass-spectrometric imaging. Cutinase hydrolysis of cutin polymer efficiently generated three oligomers, these being the dimers 9,10,18-trihydroxy-18:0 acylated by 9,10,18-trihydroxy-18:1 (*m/z* 651.43), and 9,10,18-trihydroxy-18:0 acylated by 9,10,18-trihydroxy-18:0 (*m/z* 653.43), and the lithium adduct (+ 2Li–H^+^) of the trimer 9,10,18-trihydroxy-18:0 acylated by 9,10,18-trihydroxy-18:0 acylated by 9,10,18-trihydroxy-18:0 (*m/z* 973.68). The chemical identities of these oligomers were achieved based on the accurate mass measurements made possible by the FTICR mass spectrometer, and by MS–MS experiments. Exemplary molecular images of these ions are shown in Fig. 6.

Several features of these molecular images provide confidence that they represent the spatial distribution of the cutin subfragments on the 2-dimensional surface of the apple skin. First, these molecular ions are located in the areas that have been treated with the cutinase enzyme, and the intensity of the generated ions is dependent on the concentration of the cutinase enzyme that was applied to that area. Therefore, the ions appear to be cutinase generated and their distribution represents the spatial sites of the ester bonds that are hydrolyzed to release these subfragments. The relative intensities of the signal strengths of each ion is very similar across the surface that was imaged, which would indicate that although there is a spatial asymmetry in the distribution of these cutinase-susceptible bonds, the three-pairs of ester bonds that were hydrolyzed are spatially co-localized at the resolution afforded by the imaging technology. This later resolution is primarily determined by the laser spot size that was used to generate the ions (~ 80-µm), and the rastering steps that were used to illuminate the different positions of the peel with the laser. These results therefore indicate the potential of combining cutinase enzymes and mass-spectrometric imaging as an efficient tool to map the spatial distribution of the chemistry (i.e., ester bonds) which organizes the architecture of the cutin polymer.

## Discussion

Cutin is a structural lipid support polymer that coats the aerial epidermis of terrestrial plants and acts as a “double-sided raincoat”, keeping water *in planta* and keeping water off the surface of the plant [24]. The former is of significance in restricting water exchange primarily through the stomata, whose opening and closing can be regulated to attenuate water balance and maintain gas exchange for optimal photosynthetic capability. Understanding the chemical structure of this polymer is of major significance in recognizing how plants maintain transpirational viability and optimal photosynthetic capabilities in order to capture solar energy and store it in the form of chemical energy. As a heavily cross-linked polymer of highly hydrophobic monomers, cutin is insoluble in aqueous matrices, and is probably covalently bound to other extracellular components (e.g., the cell wall). These attributes prevent addressing the intriguing question concerning the chemical nature of cutin, and how this polymer is biosynthetically assembled. Because of this complexity, cutin is often represented as an amorphous maze-like network of hydroxy-acyl monomers that are covalently cross-linked via ester-bonds. In this study, we applied methods that can systematically characterize the bonding connections and patterns of the monomeric units within cutin by the combined use of cutin hydrolytic enzymes (cutinases) and high-resolution mass spectrometry. The present study therefore illustrates analytical methods that can be combined to define the molecular architecture of cutin, the biological polymer that plays a highly significant role in establishing a barrier between the highly organized cellular processes and the environment.

Collectively therefore, we applied tandem quadrupole and time-of-flight, high resolution mass-spectrometry, coupled with the enzymatic specificity of cutinase to characterize isolated apple cutin as the model polymer to establish these techniques. Initial acid-catalyzed hydrolysis coupled with GC/MS analysis quantitatively identifies the acyl-monomers that constitute this model cutin. Consistent with prior characterizations [5, 25], these analyses identified the major monomeric acyl constituents of apple cutin as 16- and 18-carbon saturated and unsaturated FAs that are mid-chain oxygenated with hydroxyl- and epoxy-groups, and by hydroxylation at the ω-end of the acyl-chains.

The dansylation and *O*-benzyl alkylation of intact cutin identified that the hydroxyl groups associated with 16-hydroxy-16:0 and 18-hydroxy-18:2 are enriched as the terminal monomers of the cutin polymer. Therefore, these hydroxyl-groups tend not to be utilized in ester-bond formations that covalently link these monomers into the polymeric structure of cutin. Similar analysis with DmPA-modification identified that the carboxyl-groups associated with 16-hydroxy-16:0 and 18-hydroxy-C18:3 are enriched as the carboxyl ends of the cutin polymer.

**Fig. 7:**
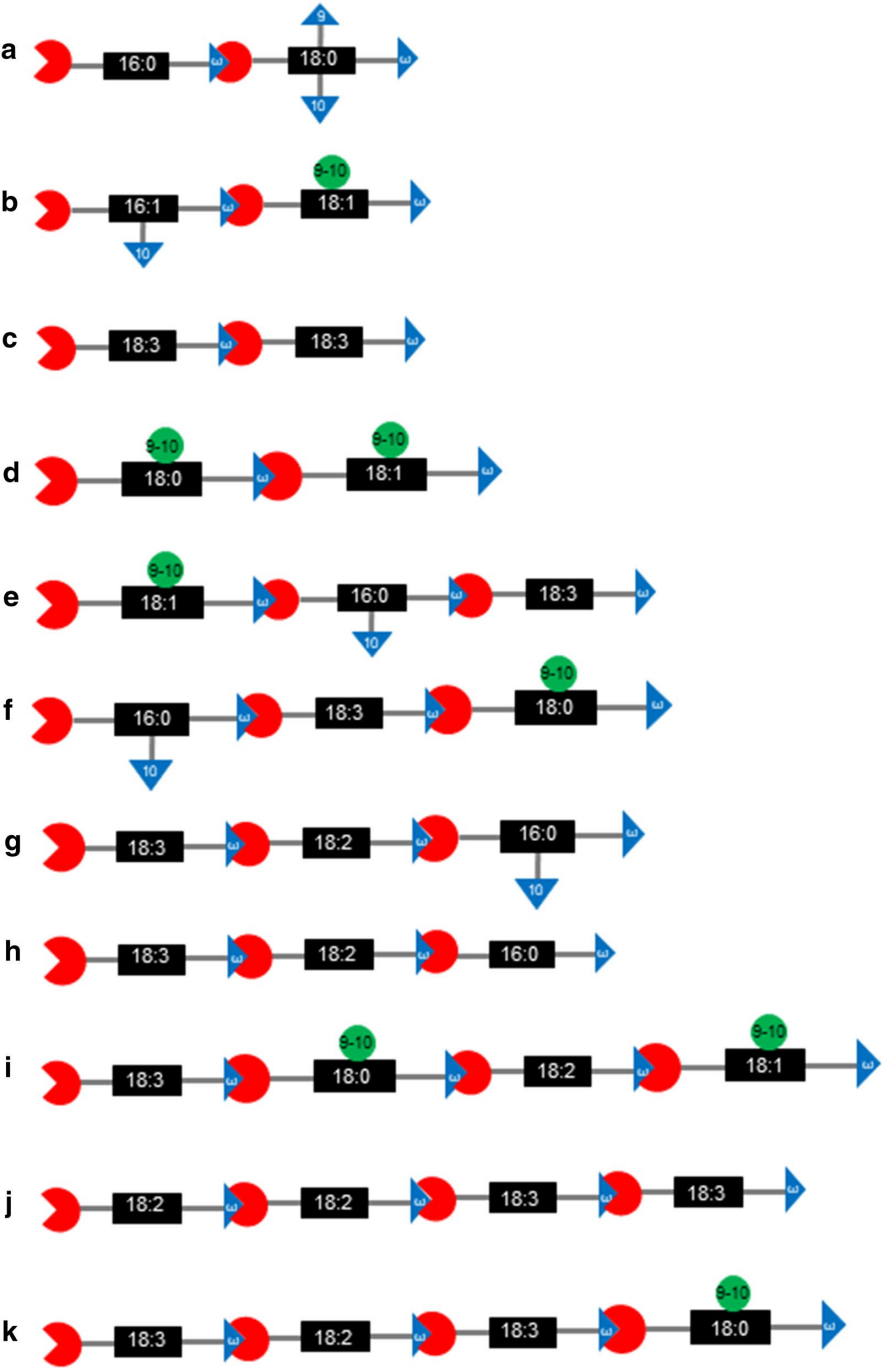
Schematic structures of cutinase-generated cutin dimers (**a**–**d**), trimers (**e**–**h**) and tetramers (**i**–**k**). The digits within the black-filled rectangle represent the nature of the acyl moiety in the standard short-hand fatty acid nomenclature. The red-shaded “PAC-MAN” symbol represents the carboxyl-group of each acyl-chain. The digits in the blue-shaded triangles represent the positions of hydroxyl groups on the acyl-chain, and the digits in the green-shaded circles represent the positions of epoxy-groups on the acyl-chain. The blue-shaded triangle juxtaposed in the red PAC-MAN schematic represents the ester bond between adjoining acyl-monomers

Partial cutinase treatment of isolated cutin released cutin oligomers consisting of 2, 3, or 4 monomeric units that are linked via ester bonds. In the context of the analogy to the primary structure of oligosaccharide [11, 12], the structure of these oligomers represents the “acyl-chain sequence” of the monomers in the cutin polymer. Analogous to the monomeric monosaccharides that constitute oligosaccharide, we propose that a common-name nomenclature or symbols would greatly facilitate the presentation of these cutin moieties. Figure 7 presents a proposed symbolic nomenclature that is adapted from a recent review [7], which may readily aid representing the primary structure of the cutin polymer. We specifically illustrate the use of this symbolic nomenclature to represent the exemplary monomers (Additional file 11: Fig. S10), dimers, trimers and tetramers (Fig. 7) whose structures were determined as isolated from apple cutin.

As with “sequencing” of polymers (e.g., peptides), one can deduce higher order structures of cutin by finding unique sequence of monomers that overlap on two fragments. For example, one could deduce from the schematic structures shown in Fig. 7, that because cutin subfragments that share common terminal monomers maybe adjoining in the intact polymer. For example, the cutin subfragment shown in Fig. 7c, maybe adjoining the subfragment shown in Fig. 7j, or the cutin subfragment shown in Fig. 7e, maybe adjoining subfragment shown in Fig. 7f. Because the overlap between the latter two subfragments is two monomeric units (the red-boxed monomers in Additional file 12: Fig. S11), rather than the single monomer overlap in the former example, one has greater confidence of this alignment of monomer-sequence.

Additional confidence in such deduction would be gained if the overlapping sequence of monomers was more extensive. Thus, additional insights would be provided by the isolation and characterization of larger oligomers, consisting of 5 or more monomeric units. This latter capability maybe further enhanced by the combined application of NMR and LC–MS/MS. Although NMR may be somewhat limited by its detection sensitivity, its higher chemical resolution would provide additional capabilities that are beyond mass spectrometry (e.g., ability to infer the regiochemistry and stereochemistry of the monomeric units). Moreover, access to additional cutinase enzymes, as identified in the current study, may be useful if these enzymes display different substrate preferences, as for example differences in the substrate specificities of α-amylases versus amyloglucosidases in the case of glycosyl hydrolases. Finally, we have also demonstrated the combined application of partial cutinase digestion with mass-spectrometric imaging of the spatial distribution of cutinase-generated fragments could provide a path to understanding the tissue-, organ- and developmental effects on the assembly of the cutin polymer. Moreover, the further characterization of the additional cutinases identified herein, would provide new tools for determining the detailed molecular structure of cutin.

## Conclusion

A strategy that is generally used to determine the primary structure of plant wall polysaccharide is applicable to determining the detailed architecture of plant cutin, a cross-linked polyester of acyl-chain monomers. Analogous to the use of hydrolase to generate monosaccharide fragments, we demonstrate that cutinase enzymes are capable of generating oligomers of cutin monomers, whose composition and structure can be deduced by high-resolution mass spectrometry. Here, we also coupled the chemical modification of “free” carboxyl and hydroxyl groups of the cutin polymer and thus “tagged” the potential ends of the cutin acyl chains. In addition, we applied mass-spectrometric imaging technology to spatially map the location of partial cutinase-generated cutin fragments.

## Methods

### Chemicals and reagents

Isopropanol, chloroform, methanol, acetonitrile and water were purchased as HPLC grade (Fisher Chemicals, Pittsburgh, PA). High purity reagents: butylated hydroxytoluene (BHT), pyridine, *N*,*O*-bis(trimethylsilyl)-trifluoroacetamide (BSTFA), monoesters of *p*-nitophenyl, heptadecanoic acid, dansyl chloride, sarkosyl, 2-benzyloxy-1-methylpyridinium triflate, magnesium oxide and alpha,alpha,alpha-trifluorotoluene were purchased from Sigma-Aldrich (Milwaukee, WI), and dimethylaminophenacyl (DmPA) bromide was purchased from Synquest Lab (Alachua, FL).

### Bioinformatics analysis

*Fusarium solini* cutinase sequence (GenBank: AAA33334) was used as the query to perform BLAST analysis at the NCBI database (https://blast.ncbi.nlm.nih.gov/Blast.cgi). Homologous sequences were identified as those that showed homology, with a cut-off e-value of (1 × 10^−4^). These sequences were clustered using the CD-HIT algorithm [19, 20] and the resulting cluster-sequences were used to construct a phylogenetic tree using the JAVA based program, Cluster Picker [21]. Based upon this phylogenetic classification, 43 diverse cutinase coding sequences were selected for expression in *E. coli*.

### Cloning, expression and purification of recombinant cutinases

The DNA encoding ORFs of the selected 43 cutinases were codon optimized for expression in *E. coli* and chemically synthesized by GenScript (Piscataway, NJ). Cutinase expression constructs, in the pET30f vector [26], was used to transform to *E. coli* Stellar™ Competent Cells (Clontech Laboratories, Mountain View, CA). Positive transformants were verified using colony PCR, restriction digestion and DNA sequencing analysis. Plasmids carrying the correct cutinase ORF sequence were transferred to *E. coli* strain One Shot BL21 (DE3) (Invitrogen, Carlsbad, CA). Expression of recombinant cutinases was achieved by growing *E. coli* cultures in 2 L flask with 500-mL of liquid LB medium containing 0.25 mM IPTG to induce cutinase expression. Cells were collected by centrifugation at 5000 rpm for 5 min after overnight of growth. Cutinase was extracted from the cell pellet using established procedures [27]. The cell pellet was suspended in a buffer consisting of 2% (w/v) sarkosyl, 2 mM imidazole, 500 mM NaCl, 20 mM potassium phosphate buffer, pH 7.4. The mixture was sonicated (20 amplitude, 7–8 times with 30 s cooling time) and incubated for 2–4 h at 15 °C. Following centrifugation (18,000*g* for 30 min) and filtration through a 0.45 µm filter, the soluble protein fraction that contained the His-tagged cutinase enzyme was passed through 5 mL polypropylene column (Thermo Fisher Scientific, Waltham, MA) containing 2 mL Ni–NTA resin (G-Bioscience, St. Louis, MO), which had previously been equilibrated with 2 mM imidazole, 500 mM NaCl, and 20 mM potassium phosphate buffer, pH 7.4. Following exhaustive washing of the column with the same buffer to remove unbound proteins, a series of buffers containing increasing concentration of imidazole (20 mM, 40 mM, 60 mM, 80 mM, 100 mM and 250 mM) were used to elute the His-tagged cutinase enzyme. The cutinase enzyme was collected in the final buffer solution, and dialyzed overnight at 4 °C, against 2 L of 20 mM potassium phosphate buffer, pH 7.4. The dialyzed protein was concentrated using Amicon ultra 15 mL centrifuge filters (Merck Millipore, Burlington, MA). Purity of cutinase protein was assessed by Coomassie-staining of SDS-PAGE gels, which showed presence of near-homogenous, pure protein preparations (greater than 95% purity). Protein concentrations were determined by Bradford assay (BioRad, Hercules, CA). The concentrated proteins were either stored at − 80 °C or used immediately for biochemical assays.

### Cutin monomer and oligomer preparation

Apple fruit (*Malus pumila*), cultivar Cameo, was obtained from a local producer. The fruit was peeled and cutin was prepared from the peels, using established procedures [28, 29]. Aliquots of 50–100 mg of fresh apple peel were delipidated by immersing for 10 min into preheated (85 °C) isopropanol containing 0.01% BHT in PTFE screw capped glass tubes. The mixture was kept on a rotatory shaker overnight at room temperature. Next day the solvent was discarded and the tissue was extracted twice with chloroform:methanol (2:1) containing 0.01% BHT, and once with methanol containing 0.01% BHT; each extraction was for a period of 2-h at room temperature. Finally, the methanol was discarded and the residue cutin preparation was dried by lyophilized to a constant dry weight.

Two different cutin depolymerization strategies were used to generate the monomers and oligomeric cutin fragments. Acid-catalyzed transmethylation was conducted by adding to the dry cutin preparation 2 mL of freshly prepared methanolic sulfuric acid (4%) and 0.200 mL of toluene, along with 10 µg of heptadecanoic acid as an internal standard. The mixture was then heated at 80 °C for 2 h. After cooling, the mixture was extracted with 4 mL dichloromethane and 1 mL of 0.9% NaCl (w/v) in 100 mM Tris–HCl buffer, pH 8.0. The organic non-polar phase was separated from the polar phase by centrifugation at 1500 rpm for 2 min. The two phases were recovered separately and dried. Silylation was conducted at 70 °C for 30 min, with 100 µL anhydrous pyridine and 100 µL BSTFA [*N*,*O*-*bis*(trimethylsilyl)-trifluoroacetamide]. The mixture was subsequently dried under a gentle stream of nitrogen gas, and the residue was dissolved in 500 µL of heptane: toluene (1:1, v/v) and subjected to GC–MS analysis.

Cutinase-mediated hydrolysis was conducted by treating 100 mg of dried apple peel with 100 µg of isolated cutinase enzyme preparation, in 20 mM potassium phosphate buffer (pH 8.0) in a final volume of 5 mL. Following incubation at 37 °C, for different time periods, the reaction was terminated by adjusting the mixture to pH 5.0, using acetic acid. The aqueous mixture was extracted with multiple aliquots of chloroform, which were pooled and taken to dryness under a gentle stream of nitrogen gas. The dried sample was dissolved in isopropanol:methanol: acetonitrile (1:1:1) solvent and subjected to GC–MS and LC–MS analysis.

### Enzyme assay

Cutinase activity was assayed with different *p*-nitropenol acyl esters, including *p*-nitrophenyl acetate, *p*-nitrophenyl butyrate, *p*-nitrophenyl octanoate, *p*-nitrophenyl laurate, *p*-nitrophenyl palmitate, and *p*-nitrophenyl stearate [28]. One unit of cutinase activity is defined as the production of 1 µmol of *p*-nitrophenol/min. The standard assay was conducted in a final volume of 250 µL, containing 200 ng of cutinase, 0.1 mM *p*-nitrophenyl ester, 20 mM Tris–HCl (pH 8), 10 mM NaCl and 10% acetonitrile. The appearance of the yellow colored *p*-nitrophenol product was spectrophotometrically monitored by the increasing absorbance at 405 nm.

### Chemical labelling of the non-esterified hydroxyl and carboxyl groups of isolated cutin

The free hydroxyl groups of intact cutin were labelled by using dansyl chloride and 2-benzyloxy-1-methylpyridinium triflate, and free carboxyl groups were esterified with *p*-dimethylaminophenacyl (DmPA) bromide according to previous protocols [30–32]. After derivatization the samples were dried under a stream of nitrogen gas and subjected to depolymerization with cutinase and acid as described above.

### GC–MS analysis of cutin monomers

Methylated and silylated cutin monomers were analyzed using an Agilent Technologies Model 7890A gas chromatograph coupled to Model 5975C mass spectrometer. GC condition were as follows: the inlet temperature was held constant at 280 °C, the helium carrier gas was at a constant flow of 1 mL min^−1^ through an Agilent 122-0112 DB-1ms column (15 m × 250 µm × 0.25 µm). The oven temperature was initially set on 70 °C, and then raised by 10 °C min^−1^ to 340 °C and held at that temperature for 6 min; transfer line was set to 280 °C.

Detection and quantification of individual cutin monomers were accomplished using an Agilent Model 5975C mass spectrometer under standard conditions with 280 °C ion source. Absolute quantification of cutin monomers were determined by comparing the ion signal to that of the heptadecanoic acid internal standard and measured surface areas of individual cutin monomers. Identification of individual cutin monomers was achieved by using AMDIS software with the NIST14 mass spectral library (http://nistmassspeclibrary.com).

### LC–MS/MS analysis of cutin oligomers

Cutinase-generated oligomeric hydrolysis products contain hydroxy fatty acid esters of apple cutin were separated UHPLC with an Eclipse plus C18 analytical column (50 mm × 2.1 mm i.d., 1.8 µm) on an Agilent Technologies 1290 Infinity instrument. The temperature of the column was at 37 °C. The mobile phase was a gradient formed by the use of Buffer A [acetonitrile:water (60:40) with 20 mM ammonium formate] and Buffer B [20 mM ammonium formate in isopropanol:acetonitrile (75:25)]. The gradient was initiated at 100% Buffer A, which was linearly changed to 100% Buffer B over 15 min-period, and was held there for 5 min. Thereafter, the gradient program was brought back to its initial conditions and held there for 2 min, with a total runtime of 24 min. A 10 µL sample injection initiated the gradient, which was run at a flow rate of 0.6 mL/min.

Eluted cutin oligomers were detected using an Agilent Technologies 6540 UHD accurate mass Q-TOF MS, which enabled the acquisition of accurate *m/z* data following MS and MS/MS experiments. An Agilent jet stream electro spray ion source was used in negative ionization mode for cutin oligomer detection. We ignore adduct ions in negative mode to reduce complexity in ion detection. Positive ionization mode was applied to detect dansylated products and *p*-dimethylaminophenacylated products. The vaporizer temperature was set at 350 °C and the capillary voltage was set at 4000 V. Identification of cutin oligomers were performed using MS full scan mode ranging from *m/z* 100 to 1700. MS scan rate was 1.5 scans per second and 1.0 scan per second for MS/MS experiment with the collision energy set at 30 V. The data was analyzed using Agilent Mass Hunter software.

### Sample preparation for MSI

Apple peel sections were prepared as mentioned in Cutin monomer and oligomer preparation section. 2 µL of cutinase enzyme (2.3 mg/mL) with dilutions of 100, 50, 25 and 0, spotted on apple tissue and was fixed in conductive indium tin oxide (ITO) coated slides using double sided conductive carbon tape. Li-DHB matrix was prepared as previously described [23]. The Li-DHB matrix, containing 2,5-dihydroxybenzoic acid (DHB) was diluted to 30 mg/mL with metnanol-H_2_O [1:1 (v/v)], which contained 100 mM LiCl. This matrix solution was applied to slide holding the apple-skin tissue using an oscillating capillary nebulizer sprayer. MALD-MS imaging was performed using a Bruker SolariX FT-ICR MS instrument equipped with a 7.0 a superconducting magnet (Bruker, Billerica, MA). MALDI-MS data was acquired in positive mode within a mass range from m/z 200 to 2000, while collecting 1 mega words of data points per scan. The laser was set to raster at 50 µm spot-size. Bruker Daltonics Flex imaging software Version 4 (Bruker, Billerica, MA) was used to collect and analyze the image data.

### Statistical analysis

The standard errors of log-ratios were calculated using a delta-method approximation [33]. Namely standard error of log-ratio = 1/ln 2 * √[(se_fr_/fr)^2^ + (se_es_/es)^2^], where se_fr_ and se_es_ are the standard errors of the average free/esterified hydroxy or carboxyl groups in the cutin monomers.

## Additional files

**Additional file 1: Table S1.** Catalog of 1243 cutinases (with identified gi numbers) distributed among 211 phylogenetic clades.

**Additional file 2: Fig. S1.** SDS-PAGE analysis of purified cutinases recombinantly expressed in *E. coli*. a) *Fusarium solani* cutinase (gi 168146); b) *Alternaria brassicicola* cutinase (gi 1169141); c) *Blastomyces gilchristii* cutinase (gi 261196822); d) *Bipolaris victoriae* cutinase (gi 578495481); e) *Catenuloplanes japonicus* cutinase (gi 703060160); f) *Oidiodendron maius* cutinase (gi 751745794).

**Additional file 3: Fig. S2.** Enzymological characterization of *F. solani* and *A. brassicicola* cutinases. Cutinase activity was determined as the rate of *p*-nitrophenyl butyrate ester hydrolysis, which was monitored by the increasing absorbance at 405 nm. a) The dependence of *F. solani* and *A. brassicicola* cutinase on the concentration of the substrate *p*-nitrophenyl butyrate ester. Error bars represent the standard deviation of three determinations. b) Substrate specificity of *F. solani* and *A. brassicicola* cutinases using the substrates *p*-nitrophenyl acetate (C2), *p*-nitrophenyl butyrate (C4), *p*-nitrophynyl octanoate (C8), *p*-nitrophynyl laurate (C12), *p*-nitrophenyl myristate (C14), *p*-nitrophenyl palmitate (C16) and *p*-nitrophenyl stearate (C18). Error bars correspond to the standard deviation of three determinations.

**Additional file 4: Fig. S3.** LC–MS and LC–MS/MS characterization of individual cutin monomers of saturated and unsaturated FAs a) trihydroxy-18-carbon FAs; b) 9,10,18-trihydroxy-18:1 (*m/z* 329.25); c) 9,10,18-trihydroxy-18:2 (*m/z* 327.22); d) dihydroxy-16-carbon FAs; e) 10,16-dihydroxy-16:0 (*m/z* 287.24); f) hydroxy-18-carbon FAs; g) 18-hydroxy-18:3 (*m/z* 293.23); h) hydroxy-9,10-epoxy-18-carbon FAs; i) 18-hydroxy-9,10-epoxy-18:0 (*m/z* 313.25); j) 18-hydroxy-9,10-epoxy-18:1 (*m/z* 311.22); k) 18-hydroxy-9,10-epoxy-18:2 (*m/z* 309.20).

**Additional file 5: Fig. S4.** LC–MS/MS fragmentation spectra of cutinase-generated cutin dimers: a) 10,16-dihydroxy-16:1 acylated by 18-hydroxy-9,10-epoxy-18:1 (*m/z* 579.42); b) 18-hydroxy-18:3 acylated by 18-hydroxy-18:3 (*m/z* 569.47); c) 18-hydroxy-9,10-epoxy-18:0 acylated by 18-hydroxy-9,10-epoxy-18:1 (*m/z* 607.45).

**Additional file 6: Fig. S5.** LC–MS/MS fragmentation spectra of cutinase-generated cutin trimers: a) 10,16-dihydroxy-16:0 acylated by 18-hydroxy-18:3, which is acylated by 18-hydroxy-9,10-epoxy-18:0 (*m/z* 859.66); b) 18-hydroxy-18:3 acylated by 18-hydroxy-18:2, which is acylated by 10,16-dihydroxy-16:0 (*m/z* 841.65); c) 18-hydroxy-18:3 acylated by 18-hydroxy-18:2, which is acylated by 16-hydroxy-16:0 (*m/z* 825.66).

**Additional file 7: Fig. S6.** LC–MS/MS fragmentation spectra of cutinase-generated cutin tetramers: a) 18-hydroxy-18:2 acylated by 18-hydroxy-18:2, which is acylated by 18-hydroxy-18:3, which is further acylated by 18-hydroxy-18:3 (*m/z* 1125.92); b) 18-hydroxy-18:3 acylated by 18-hydroxy-18:2, which is acylated by 18-hydroxy-18:3, which is further acylated by 18-hydroxy-9,10-epoxy-18:0 (*m/z* 1143.88).

**Additional file 8: Fig. S7.** LC–MS/MS identification of dansyl-derivatized hydroxy-FAs a) 18-hydroxy-18:2 (*m/z* 530.29); and b) 18-hydroxy-9,10-epoxy-18:0 (*m/z* 548.32).

**Additional file 9: Fig. S8.** GC/MS identification of silylated, benzyl-*O*-alkylated hydroxy-FA methyl esters: a) non-alkylated 10,16-dihydroxy-16:0; b) ω-alkylated 10,16-dihydroxy-16:0; c) 10-alkylated 10,16-dihydroxy-16:0; d) non-alkylated 9,10,18-trihydroxy-18:0; e) 10- or 9-alkylated 9,10,18-trihydroxy-18:0.

**Additional file 10: Fig. S9.** LC–MS/MS identification of DmPA-derivatized a) 18-hydroxy-18:3 (*m/z* 458.32); and b) 9,10,18-trihydroxy-18:0 0 (*m/z* 492.30).

**Additional file 11: Fig. S10.** Schematic representation of cutin monomers. The digits within the black-filled rectangle represent the nature of the acyl moiety in the standard short-hand fatty acid nomenclature. The red-shaded “PAC-MAN” symbol represents the carboxyl-group of each acyl-chain. The digits in the blue-shaded triangles represent the positions of hydroxyl groups on the acyl-chain, and the digits in the green-shaded circles represent the positions of epoxy-groups on the acyl-chain. The blue-shaded triangle juxtaposed in the red PAC-MAN schematic represents the ester bond between adjoining acyl-monomers. a) Hexadecanoic acid (palmitic acid). b) 16-Hydroxyhexadecanoic acid. c) 10,16-Dihydroxyhexadecanoic acid. d) 2-Hydroxyhexadecanoic acid. e) Hexadecanedioic acid. f) Octadecanoic acid (stearic acid). g) 9-Octadecenoic acid (oleic acid). h) 9,12-octadecadienoic acid (linoleic acid). i) 18-hydroxy-9-octadecenoic acid. j) 18-hydroxy-9,12-octadecenoic acid. k) 18-hydroxy-9,12,15-octadecenoic acid. l) 9,10,18-trihydroxyoctadecanoic acid. m) 9,10,18-trihydroxyoctadec-12-enoic acid. n) 9,10,18-trihydroxyoctadec-12,15-dienoic acid. o) 9,10-dihydroxy-octadecanedioic acid. p) 18-hydroxy-9,10-epoxoyctadecanoic acid. q) 18-hydroxy-9,10-epoxyoctadeca-12-enoic acid. r) Eicosanoic acid. s) 8,11,14-eicosatrienoic acid. t) 2-hydroxydocosanoic acid. u) 2-hydroxytetracosanoic acid. v) 1-hexacosanol. w) 1-octacosanol. x) Triacontanoic acid. y) 1-triacontanol.

**Additional file 12: Fig. S11.** Schematic representation of overlapping region of cutin subfragments. The red-box identifies monomer overlaps among different subfragments that may indicate they are adjoining in the cutin polymer.

## Abbreviations

GC–MS: gas chromatography–mass spectrometry
LC–MS: liquid chromatography–mass spectrometry
Q-TOF: quadrupole time-of-flight
MALDI: matrix-assisted laser desorption/ionization
CD-HIT: Cluster Database at High Identity with Tolerance
DmPA: *p*-dimethylaminophenacyl
Li-DHB: lithium-doped 2,5-dihydroxybenzoic acid
