# Supplementary material for: Combined use of cutinase and high-resolution mass-spectrometry to query the molecular architecture of cutin
Source: Plant Methods. 2018 Dec 26;14:117. doi: 10.1186/s13007-018-0384-6 (PMC6306009; doi:10.1186/s13007-018-0384-6)
Supplement: Supplementary file 1 — Additional file 1: Table S1. Catalog of 1243 cutinases (with identified gi numbers) distributed among 211 phylogenetic clades. [file 13007_2018_384_MOESM1_ESM.docx]

| **Table S1.** Catalog of 1243 cutinases distributed among 211 phylogenetic clades | | |
| --- | --- | --- |
| **Phylogenetic Clade**† | **GI numbers** | **Source micro organism** |
| **1** | 565996190 | cutinase [Mycobacterium neoaurum] |
| **1** | 565996191 | MULTISPECIES: cutinase [Mycobacterium] |
| **1** | 642752689 | cutinase [Mycobacterium neoaurum] |
| **1** | 662771341 | cutinase [Mycobacterium neoaurum] |
| **1** | 662771342 | cutinase [Mycobacterium neoaurum] |
| **1** | 738444906 | cutinase [Mycobacterium cosmeticum] |
| **1** | 780286266 | hypothetical protein [Mycobacterium neoaurum] |
| **2** | 356480608 | serine esterase, cutinase [Mycobacterium thermoresistibile ATCC 19527] |
| **2** | 808804699 | cutinase [Mycobacterium sp. UM_NZ2] |
| **3** | 433300064 | Cutinase [Mycobacterium smegmatis JS623] |
| **3** | 499881621 | MULTISPECIES: cutinase [Mycobacterium] |
| **3** | 656081678 | cutinase [Mycobacterium sp. URHB0044] |
| **3** | 829458314 | cutinase [Mycobacterium sp. EPa45] |
| **4** | 518944423 | MULTISPECIES: hypothetical protein [Mycobacterium] |
| **4** | 759680642 | cutinase [Mycobacterium sp. UNC280MFTsu5.1] |
| **5** | 576429276 | cutinase family protein [Mycobacterium xenopi 3993] |
| **5** | 779913307 | cutinase [Mycobacterium kyorinense] |
| **6** | 399234660 | Cutinase Cut1 [Mycobacterium smegmatis str. MC2 155] |
| **6** | 489976680 | MULTISPECIES: cutinase [Mycobacterium] |
| **6** | 518174066 | MULTISPECIES: cutinase [Mycobacterium] |
| **6** | 656079286 | cutinase [Mycobacterium sp. URHD0025] |
| **6** | 738479748 | cutinase [Mycobacterium mageritense] |
| **6** | 738498538 | cutinase [Mycobacterium vulneris] |
| **6** | 764950286 | cutinase [Mycobacterium septicum] |
| **7** | 359820143 | Cutinase [Mycobacterium rhodesiae NBB3] |
| **7** | 489986281 | cutinase [Mycobacterium phlei] |
| **7** | 491846735 | cutinase [Mycobacterium hassiacum] |
| **7** | 750241590 | cutinase, partial [Mycobacterium tusciae] |
| **7** | 820790316 | cutinase [Mycobacterium elephantis] |
| **7** | 857807275 | Cutinase [Mycobacterium sp. GPK 1020] |
| **7** | 858009925 | Cutinase [Mycobacterium sp. WCM 7299] |
| **8** | 808801616 | MULTISPECIES: cutinase [Mycobacterium] |
| **8** | 809062074 | cutinase [Mycobacterium sp. UM_Kg1] |
| **9** | 521366078 | serine esterase, cutinase family protein [Mycobacterium yongonense 05-1390] |
| **9** | 738510462 | cutinase [Mycobacterium intracellulare] |
| **10** | 518815103 | hypothetical protein [Mycobacterium sp. 141] |
| **10** | 597303388 | Cutinase [Mycobacterium cosmeticum] |
| **10** | 738468459 | cutinase [Mycobacterium sp. 155] |
| **10** | 738475676 | cutinase [Mycobacterium sp. UM_WWY] |
| **10** | 738485244 | cutinase [Mycobacterium sp. URHB0044] |
| **11** | 110817180 | probable cutinase [Rhodococcus jostii RHA1] |
| **11** | 491384688 | cutinase [Rhodococcus opacus] |
| **11** | 739314021 | cutinase [Rhodococcus sp. UNC363MFTsu5.1] |
| **12** | 738950473 | MULTISPECIES: cutinase [Actinobacteria] |
| **12** | 759972329 | cutinase [Nocardia thailandica] |
| **13** | 443886369 | putative cutinase [Gordonia soli NBRC 108243] |
| **13** | 750221286 | cutinase [Gordonia effusa] |
| **13** | 797060315 | cutinase [Williamsia sp. ARP1] |
| **14** | 377527967 | hypothetical protein GOSPT_062_00240 [Gordonia sputi NBRC 100414] |
| **14** | 441446205 | hypothetical protein GOACH_15_00560 [Gordonia aichiensis NBRC 108223] |
| **14** | 464803770 | cutinase family protein [Gordonia sp. NB4-1Y] |
| **14** | 493412686 | cutinase [Gordonia polyisoprenivorans] |
| **14** | 748736558 | cutinase [Gordonia otitidis] |
| **15** | 403400333 | hypothetical protein GONAM_61_00220 [Gordonia namibiensis NBRC 108229] |
| **15** | 441450416 | hypothetical protein GOAMI_33_00350 [Gordonia amicalis NBRC 100051 = JCM 11271] |
| **15** | 490121900 | cutinase [Gordonia terrae] |
| **15** | 491337504 | cutinase [Gordonia rubripertincta] |
| **15** | 493957904 | hypothetical protein [Gordonia paraffinivorans] |
| **15** | 498813489 | MULTISPECIES: cutinase [Gordonia] |
| **15** | 502598528 | cutinase [Gordonia bronchialis] |
| **15** | 755022159 | cutinase [Gordonia alkanivorans] |
| **16** | 494533220 | cutinase [Gordonia araii] |
| **16** | 502598519 | cutinase [Gordonia bronchialis] |
| **17** | 493954342 | hypothetical protein [Gordonia sihwensis] |
| **17** | 495655780 | hypothetical protein [Gordonia malaquae] |
| **17** | 652524411 | cutinase [Gordonia shandongensis] |
| **17** | 750214511 | cutinase [Gordonia neofelifaecis] |
| **18** | 145216250 | cutinase [Mycobacterium gilvum PYR-GCK] |
| **18** | 490028957 | cutinase [Mycobacterium vaccae] |
| **18** | 500106904 | cutinase [Mycobacterium vanbaalenii] |
| **18** | 500221830 | cutinase [Mycobacterium gilvum] |
| **18** | 504631193 | cutinase [Mycobacterium chubuense] |
| **18** | 638979749 | cutinase [Mycobacterium iranicum] |
| **18** | 662771456 | MULTISPECIES: cutinase [Mycobacterium] |
| **18** | 674843906 | MULTISPECIES: cutinase [Mycobacterium] |
| **18** | 750246572 | cutinase [Mycobacterium vaccae] |
| **18** | 754167655 | None |
| **18** | 759697876 | cutinase [Mycobacterium rufum] |
| **18** | 810937932 | cutinase [Mycobacterium obuense] |
| **18** | 860420920 | cutinase [Mycobacterium chubuense] |
| **18** | 860584507 | cutinase [Mycobacterium chlorophenolicum] |
| **19** | 400331457 | serine esterase cutinase [Mycobacterium colombiense CECT 3035] |
| **19** | 489515590 | MULTISPECIES: cutinase [Mycobacterium tuberculosis complex] |
| **19** | 489970148 | MULTISPECIES: cutinase [Mycobacterium avium complex (MAC)] |
| **19** | 489996643 | cutinase [Mycobacterium tuberculosis] |
| **19** | 494291541 | cutinase [Mycobacterium parascrofulaceum] |
| **19** | 495538641 | MULTISPECIES: cutinase [Mycobacterium avium complex (MAC)] |
| **19** | 500062021 | cutinase [Mycobacterium ulcerans] |
| **19** | 501365195 | cutinase [Mycobacterium marinum] |
| **19** | 505104579 | cutinase [Mycobacterium canettii] |
| **19** | 522812366 | serine esterase cutinase family [Mycobacterium marinum] |
| **19** | 523651424 | serine esterase cutinase family [Mycobacterium sp. 012931] |
| **19** | 556612605 | cutinase [Mycobacterium kansasii] |
| **19** | 738409140 | cutinase [Mycobacterium asiaticum] |
| **19** | 738411098 | cutinase [Mycobacterium asiaticum] |
| **19** | 738454484 | cutinase [Mycobacterium kansasii] |
| **19** | 738466261 | cutinase [Mycobacterium gastri] |
| **19** | 738516781 | cutinase [Mycobacterium triplex] |
| **19** | 757677482 | cutinase [Mycobacterium avium] |
| **19** | 764942033 | cutinase [Mycobacterium simiae] |
| **19** | 806824273 | cutinase precursor Cut5 [Mycobacterium lentiflavum] |
| **19** | 806833546 | cutinase [Mycobacterium nebraskense] |
| **19** | 808662167 | serine esterase cutinase [Mycobacterium europaeum] |
| **19** | 829095024 | cutinase [Mycobacterium haemophilum] |
| **20** | 499881862 | MULTISPECIES: cutinase [Mycobacterium] |
| **20** | 656082211 | cutinase [Mycobacterium sp. URHB0044] |
| **21** | 124601010 | cutinase cut1 [Mycobacterium tuberculosis C] |
| **21** | 505106076 | None |
| **21** | 620813607 | cutinase Cut1, partial [Mycobacterium tuberculosis MD20344] |
| **21** | 622926540 | cutinase Cut1 [Mycobacterium tuberculosis H1249] |
| **21** | 829094643 | cutinase [Mycobacterium haemophilum] |
| **22** | 333487705 | cutinase Cut5 [Mycobacterium sinense] |
| **22** | 638969488 | cutinase [Mycobacterium sp. UM_WGJ] |
| **22** | 759617371 | hypothetical protein, partial [Mycobacterium avium] |
| **22** | 806841101 | cutinase [Mycobacterium arupense] |
| **22** | 808801348 | cutinase [Mycobacterium sp. UM_NZ2] |
| **22** | 809005325 | cutinase [Mycobacterium sp. UM_Kg17] |
| **22** | 809065629 | cutinase [Mycobacterium sp. UM_Kg1] |
| **22** | 829148953 | cutinase [Mycobacterium heraklionense] |
| **23** | 315263690 | Cutinase [Mycobacterium gilvum Spyr1] |
| **23** | 390616586 | Cutinase [Mycobacterium chubuense NBB4] |
| **23** | 500225189 | cutinase [Mycobacterium gilvum] |
| **23** | 638991766 | cutinase [Mycobacterium iranicum] |
| **23** | 750243454 | hypothetical protein, partial [Mycobacterium vaccae] |
| **23** | 759687023 | cutinase [Mycobacterium rufum] |
| **23** | 810937662 | cutinase [Mycobacterium obuense] |
| **23** | 859092089 | Cutinase [Mycobacterium chlorophenolicum] |
| **23** | 859092926 | Cutinase [Mycobacterium chubuense] |
| **24** | 433297633 | Cutinase [Mycobacterium smegmatis JS623] |
| **24** | 493289458 | serine esterase [Mycobacterium tusciae] |
| **24** | 503977751 | serine esterase [Mycobacterium rhodesiae] |
| **24** | 504689887 | serine esterase [Mycobacterium smegmatis] |
| **24** | 505117652 | hypothetical protein [Mycobacterium smegmatis] |
| **24** | 517428428 | hypothetical protein [Mycobacterium sp. 155] |
| **24** | 518948502 | hypothetical protein [Mycobacterium sp. 360MFTsu5.1] |
| **24** | 602523287 | serine esterase, cutinase [Mycobacterium vulneris] |
| **24** | 602543879 | serine esterase, cutinase [Mycobacterium mageritense DSM 44476] |
| **24** | 633839154 | serine esterase, cutinase [Mycobacterium farcinogenes] |
| **24** | 639009793 | MULTISPECIES: serine esterase [Mycobacterium] |
| **24** | 656079515 | serine esterase [Mycobacterium sp. URHD0025] |
| **24** | 657267197 | serine esterase [Mycobacterium sp. UM_WWY] |
| **24** | 738512085 | serine esterase [Mycobacterium sp. 141] |
| **24** | 746332366 | serine esterase [Mycobacterium setense] |
| **24** | 759683229 | MULTISPECIES: serine esterase [Mycobacterium] |
| **24** | 764945388 | None |
| **24** | 777215276 | Cutinase Cut1 [Mycobacterium smegmatis] |
| **24** | 808670676 | serine esterase, cutinase [Mycobacterium conceptionense] |
| **24** | 858018895 | serine esterase, cutinase [Mycobacterium fortuitum subsp. fortuitum DSM 46621] |
| **25** | 738520333 | cutinase [Mycobacterium genavense] |
| **25** | 738520564 | cutinase [Mycobacterium triplex] |
| **25** | 806821634 | cutinase Cut3 [Mycobacterium lentiflavum] |
| **26** | 495046642 | cutinase [Mycobacterium colombiense] |
| **26** | 508734966 | cutinase Cut3 [Mycobacterium avium subsp. hominissuis TH135] |
| **26** | 564166206 | cutinase [Mycobacterium avium subsp. silvaticum ATCC 49884] |
| **26** | 564190045 | cutinase [Mycobacterium avium subsp. hominissuis 10-4249] |
| **26** | 564199821 | cutinase [Mycobacterium avium subsp. hominissuis 10-5606] |
| **26** | 750341108 | cutinase [Mycobacterium parascrofulaceum] |
| **26** | 806835251 | cutinase [Mycobacterium nebraskense] |
| **27** | 356477509 | cutinase Cut1 [Mycobacterium thermoresistibile ATCC 19527] |
| **27** | 490029059 | cutinase [Mycobacterium vaccae] |
| **27** | 500046014 | cutinase [Mycobacterium smegmatis] |
| **27** | 602540059 | serine esterase cutinase [Mycobacterium mageritense DSM 44476] |
| **27** | 738503402 | cutinase [Mycobacterium smegmatis] |
| **28** | 383338345 | serine esterase, cutinase [Mycobacterium xenopi RIVM700367] |
| **28** | 576482867 | cutinase family protein [Mycobacterium xenopi 4042] |
| **28** | 738474167 | cutinase [Mycobacterium sp. UM_WWY] |
| **29** | 503978955 | cutinase [Mycobacterium rhodesiae] |
| **29** | 653080863 | cutinase [Mycobacterium tusciae] |
| **29** | 764949732 | cutinase [Mycobacterium septicum] |
| **29** | 857808630 | Cutinase [Mycobacterium sp. GPK 1020] |
| **29** | 858007237 | Cutinase [Mycobacterium sp. WCM 7299] |
| **30** | 635709879 | hypothetical protein Y900_011625 [Mycobacterium aromaticivorans JS19b1 = JCM 16368] |
| **30** | 656076548 | hypothetical protein [Mycobacterium sp. URHD0025] |
| **31** | 638967304 | cutinase [Mycobacterium sp. UM_WGJ] |
| **31** | 809074017 | cutinase [Mycobacterium sp. UM_Kg1] |
| **31** | 829156727 | cutinase [Mycobacterium heraklionense] |
| **32** | 576437239 | cutinase family protein [Mycobacterium xenopi 3993] |
| **32** | 576472185 | cutinase family protein [Mycobacterium xenopi 4042] |
| **32** | 748631221 | cutinase [Mycobacterium xenopi] |
| **33** | 504629335 | cutinase [Mycobacterium chubuense] |
| **33** | 759700131 | cutinase [Mycobacterium rufum] |
| **34** | 353192324 | cutinase [Mycobacterium rhodesiae JS60] |
| **34** | 635709614 | cutinase [Mycobacterium aromaticivorans JS19b1 = JCM 16368] |
| **34** | 829463753 | cutinase [Mycobacterium sp. EPa45] |
| **35** | 501362599 | MULTISPECIES: cutinase [Mycobacterium] |
| **35** | 556617579 | cutinase [Mycobacterium kansasii] |
| **35** | 574762590 | cutinase [Mycobacterium gastri 'Wayne'] |
| **35** | 576440566 | cutinase family protein [Mycobacterium xenopi 3993] |
| **35** | 738443198 | cutinase [Mycobacterium kansasii] |
| **35** | 738449672 | cutinase [Mycobacterium kansasii] |
| **35** | 738454628 | cutinase [Mycobacterium kansasii] |
| **35** | 738463590 | cutinase [Mycobacterium gastri] |
| **35** | 748631369 | cutinase [Mycobacterium xenopi] |
| **36** | 518943267 | MULTISPECIES: cutinase [Mycobacterium] |
| **36** | 759681874 | cutinase [Mycobacterium sp. UNC280MFTsu5.1] |
| **37** | 183176263 | cutinase Cfp21 [Mycobacterium marinum M] |
| **37** | 308215329 | cutinase cfp21 [Mycobacterium tuberculosis SUMu001] |
| **37** | 494299678 | cutinase [Mycobacterium parascrofulaceum] |
| **37** | 499251770 | cutinase [Mycobacterium avium] |
| **37** | 505106228 | None |
| **37** | 556021876 | cutinase [Mycobacterium kansasii ATCC 12478] |
| **37** | 574761547 | cutinase [Mycobacterium gastri 'Wayne'] |
| **37** | 576412764 | cutinase family protein [Mycobacterium kansasii 732] |
| **37** | 623275073 | cutinase CFP21 [Mycobacterium tuberculosis MAL020174] |
| **37** | 656999746 | hypothetical protein K883_04198 [Mycobacterium sp. TKK-01-0059] |
| **37** | 738408316 | cutinase [Mycobacterium asiaticum] |
| **37** | 738477014 | cutinase [Mycobacterium sp. 012931] |
| **37** | 738520956 | cutinase [Mycobacterium triplex] |
| **37** | 750347913 | cutinase [Mycobacterium colombiense] |
| **37** | 764935607 | cutinase [Mycobacterium simiae] |
| **37** | 800176908 | cutinase cfp21 [Mycobacterium tuberculosis] |
| **37** | 806834887 | cutinase [Mycobacterium nebraskense] |
| **37** | 808579032 | serine esterase, cutinase [Mycobacterium bohemicum DSM 44277] |
| **37** | 808658903 | serine esterase, cutinase [Mycobacterium europaeum] |
| **38** | 494291537 | cutinase [Mycobacterium parascrofulaceum] |
| **38** | 495047260 | cutinase [Mycobacterium colombiense] |
| **38** | 808577817 | cutinase Cfp21 [Mycobacterium bohemicum DSM 44277] |
| **38** | 808662164 | cutinase Cfp21 [Mycobacterium europaeum] |
| **39** | 500104539 | cutinase [Mycobacterium austroafricanum] |
| **39** | 503237106 | None |
| **39** | 504628735 | cutinase [Mycobacterium chubuense] |
| **39** | 738515212 | cutinase [Mycobacterium iranicum] |
| **39** | 750246200 | cutinase [Mycobacterium vaccae] |
| **39** | 759692257 | cutinase [Mycobacterium rufum] |
| **39** | 859095338 | Cutinase [Mycobacterium obuense] |
| **39** | 860415643 | cutinase [Mycobacterium chubuense] |
| **40** | 296179726 | cutinase [Segniliparus rotundus DSM 44985] |
| **40** | 540598958 | hypothetical protein HMPREF9336_01509 [Segniliparus rugosus ATCC BAA-974] |
| **41** | 119954214 | cutinase [Mycobacterium vanbaalenii PYR-1] |
| **41** | 359821119 | Cutinase [Mycobacterium rhodesiae NBB3] |
| **41** | 490031214 | cutinase [Mycobacterium vaccae] |
| **41** | 493287665 | cutinase [Mycobacterium tusciae] |
| **41** | 504626500 | cutinase [Mycobacterium chubuense] |
| **41** | 638984548 | cutinase [Mycobacterium iranicum] |
| **41** | 656081332 | cutinase [Mycobacterium sp. URHB0044] |
| **41** | 738488187 | cutinase, partial [Mycobacterium sp. URHB0044] |
| **41** | 759687411 | cutinase [Mycobacterium rufum] |
| **41** | 857806889 | Cutinase [Mycobacterium sp. GPK 1020] |
| **41** | 857809295 | cutinase [Mycobacterium sp. GPK 1020] |
| **41** | 858007094 | Cutinase [Mycobacterium sp. WCM 7299] |
| **41** | 860421984 | cutinase [Mycobacterium chubuense] |
| **41** | 860438016 | cutinase [Mycobacterium obuense] |
| **41** | 860577346 | cutinase [Mycobacterium chlorophenolicum] |
| **42** | 647292675 | cutinase [Mycobacterium genavense] |
| **42** | 738520946 | cutinase [Mycobacterium triplex] |
| **42** | 738524198 | cutinase [Mycobacterium triplex] |
| **42** | 764935605 | cutinase [Mycobacterium simiae] |
| **42** | 806821920 | cutinase Cut1 [Mycobacterium lentiflavum] |
| **42** | 806822333 | cutinase Cut1 [Mycobacterium lentiflavum] |
| **43** | 405131879 | putative cutinase [Mycobacterium indicus pranii MTCC 9506] |
| **43** | 41396473 | hypothetical protein MAP_2020 [Mycobacterium avium subsp. paratuberculosis K-10] |
| **43** | 41398424 | hypothetical protein MAP_3495c [Mycobacterium avium subsp. paratuberculosis K-10] |
| **43** | 504192802 | MULTISPECIES: cutinase [Mycobacterium avium complex (MAC)] |
| **43** | 518563883 | cutinase [Mycobacterium avium] |
| **43** | 564221506 | cutinase [Mycobacterium avium subsp. paratuberculosis 10-8425] |
| **43** | 738410169 | cutinase [Mycobacterium asiaticum] |
| **43** | 750347589 | MULTISPECIES: cutinase [Mycobacterium] |
| **43** | 750347846 | cutinase [Mycobacterium colombiense] |
| **43** | 750348669 | cutinase [Mycobacterium colombiense] |
| **43** | 756413334 | cutinase [Mycobacterium avium] |
| **43** | 779908603 | cutinase [Mycobacterium kyorinense] |
| **43** | 806833631 | cutinase [Mycobacterium nebraskense] |
| **43** | 806835015 | cutinase [Mycobacterium nebraskense] |
| **43** | 806838475 | cutinase, partial [Mycobacterium nebraskense] |
| **43** | 808578711 | serine esterase cutinase [Mycobacterium bohemicum DSM 44277] |
| **43** | 808658578 | cutinase Cut1 [Mycobacterium europaeum] |
| **43** | 808659289 | serine esterase cutinase [Mycobacterium europaeum] |
| **43** | 808661112 | serine esterase cutinase [Mycobacterium europaeum] |
| **43** | 808662693 | serine esterase cutinase [Mycobacterium europaeum] |
| **44** | 517427815 | cutinase [Mycobacterium sp. 155] |
| **44** | 518816046 | cutinase [Mycobacterium sp. 141] |
| **44** | 657266974 | cutinase [Mycobacterium sp. UM_WWY] |
| **45** | 503594490 | cutinase [Mycobacterium sinense] |
| **45** | 638967306 | cutinase [Mycobacterium sp. UM_WGJ] |
| **45** | 759617558 | cutinase [Mycobacterium avium] |
| **45** | 806840729 | MULTISPECIES: cutinase [Mycobacterium] |
| **45** | 808800942 | cutinase [Mycobacterium sp. UM_NZ2] |
| **45** | 809074031 | cutinase [Mycobacterium sp. UM_Kg1] |
| **45** | 829156746 | cutinase [Mycobacterium heraklionense] |
| **46** | 500224140 | cutinase [Mycobacterium gilvum] |
| **46** | 638987856 | cutinase [Mycobacterium iranicum] |
| **47** | 363995785 | cutinase cut3 precursor [Mycobacterium abscessus subsp. bolletii BD] |
| **47** | 392079513 | putative cutinase cut2 [Mycobacterium abscessus 5S-0422] |
| **47** | 491197622 | cutinase [Mycobacterium abscessus] |
| **47** | 491240188 | cutinase [Mycobacterium abscessus] |
| **47** | 501253670 | cutinase [Mycobacterium abscessus] |
| **47** | 662145785 | cutinase [Mycobacterium abscessus] |
| **47** | 662145788 | cutinase [Mycobacterium abscessus] |
| **47** | 759017620 | cutinase [Mycobacterium immunogenum] |
| **48** | 493289044 | cutinase [Mycobacterium tusciae] |
| **48** | 503976472 | cutinase [Mycobacterium rhodesiae] |
| **49** | 407374700 | phospholipase/Carboxylesterase family protein [Mycobacterium hassiacum DSM 44199] |
| **49** | 500051309 | serine esterase [Mycobacterium smegmatis] |
| **49** | 602545268 | serine esterase, cutinase [Mycobacterium mageritense DSM 44476] |
| **50** | 491211342 | cutinase [Mycobacterium abscessus] |
| **50** | 662143120 | cutinase [Mycobacterium abscessus] |
| **50** | 759020205 | cutinase [Mycobacterium immunogenum] |
| **51** | 808801299 | cutinase [Mycobacterium sp. UM_NZ2] |
| **51** | 809027345 | cutinase [Mycobacterium sp. UM_Kg27] |
| **51** | 809065638 | cutinase [Mycobacterium sp. UM_Kg1] |
| **51** | 829150368 | cutinase [Mycobacterium heraklionense] |
| **52** | 633833577 | cutinase Cfp21 [Mycobacterium farcinogenes] |
| **52** | 738487937 | cutinase [Mycobacterium mageritense] |
| **52** | 746276953 | cutinase [Mycobacterium setense] |
| **53** | 489982021 | MULTISPECIES: cutinase [Mycobacterium] |
| **53** | 602520368 | cutinase Cfp21 [Mycobacterium vulneris] |
| **53** | 656076779 | cutinase [Mycobacterium sp. URHD0025] |
| **54** | 490029807 | cutinase [Mycobacterium vaccae] |
| **54** | 500104305 | MULTISPECIES: cutinase [Mycobacterium] |
| **55** | 390616390 | Cutinase [Mycobacterium chubuense NBB4] |
| **55** | 759692649 | cutinase [Mycobacterium rufum] |
| **55** | 810933052 | cutinase [Mycobacterium obuense] |
| **55** | 859106938 | Cutinase [Mycobacterium chubuense] |
| **55** | 860576682 | cutinase [Mycobacterium chlorophenolicum] |
| **56** | 494733240 | cutinase [Segniliparus rugosus] |
| **56** | 502902113 | cutinase [Segniliparus rotundus] |
| **57** | 495987303 | hypothetical protein [Rhodococcus sp. AW25M09] |
| **57** | 653334177 | MULTISPECIES: cutinase [Rhodococcus] |
| **57** | 653364807 | cutinase [Rhodococcus sp. UNC23MFCrub1.1] |
| **57** | 694031698 | cutinase [Rhodococcus fascians] |
| **57** | 694050206 | cutinase [Rhodococcus fascians] |
| **57** | 755779546 | cutinase [Rhodococcus sp. MEB064] |
| **57** | 852245210 | cutinase [Rhodococcus fascians] |
| **58** | 738450522 | cutinase [Mycobacterium cosmeticum] |
| **58** | 738470142 | cutinase [Mycobacterium sp. 360MFTsu5.1] |
| **58** | 738511675 | MULTISPECIES: cutinase [Mycobacterium] |
| **58** | 759684931 | cutinase [Mycobacterium sp. UNC280MFTsu5.1] |
| **58** | 780285881 | cutinase [Mycobacterium neoaurum] |
| **59** | 289696172 | LOW QUALITY PROTEIN: serine esterase, partial [Mycobacterium tuberculosis EAS054] |
| **59** | 489997684 | MULTISPECIES: cutinase [Mycobacterium tuberculosis complex] |
| **59** | 500059197 | MULTISPECIES: cutinase [Mycobacterium] |
| **59** | 556614978 | cutinase [Mycobacterium kansasii] |
| **59** | 738401161 | cutinase [Mycobacterium asiaticum] |
| **59** | 738447150 | cutinase [Mycobacterium kansasii] |
| **59** | 763229809 | cutinase [Mycobacterium bovis] |
| **60** | 518567979 | cutinase [Mycobacterium avium] |
| **60** | 638976408 | MULTISPECIES: cutinase [Mycobacterium] |
| **60** | 752669916 | None |
| **60** | 806842084 | cutinase [Mycobacterium arupense] |
| **60** | 808804278 | cutinase [Mycobacterium sp. UM_NZ2] |
| **60** | 809067571 | cutinase [Mycobacterium sp. UM_Kg1] |
| **60** | 829160496 | cutinase [Mycobacterium heraklionense] |
| **61** | 503596130 | cutinase [Mycobacterium sinense] |
| **61** | 518567978 | cutinase [Mycobacterium avium] |
| **61** | 638976405 | MULTISPECIES: cutinase [Mycobacterium] |
| **61** | 806842083 | cutinase [Mycobacterium arupense] |
| **61** | 808804276 | cutinase [Mycobacterium sp. UM_NZ2] |
| **61** | 809067569 | cutinase [Mycobacterium sp. UM_Kg1] |
| **61** | 829160495 | cutinase [Mycobacterium heraklionense] |
| **62** | 503978529 | cutinase [Mycobacterium rhodesiae] |
| **62** | 518343645 | hypothetical protein [Mycobacterium neoaurum] |
| **62** | 738510999 | cutinase [Mycobacterium sp. UNCCL9] |
| **63** | 648564843 | cutinase [Mycobacterium sp. 155] |
| **63** | 657266596 | cutinase [Mycobacterium sp. UM_WWY] |
| **63** | 738439524 | MULTISPECIES: cutinase [Mycobacterium] |
| **63** | 738480569 | cutinase [Mycobacterium mageritense] |
| **63** | 738494097 | cutinase [Mycobacterium vulneris] |
| **63** | 738512730 | cutinase [Mycobacterium sp. 141] |
| **63** | 738527884 | cutinase [Mycobacterium sp. URHD0025] |
| **63** | 740780461 | MULTISPECIES: cutinase [Mycobacterium] |
| **63** | 746279029 | cutinase [Mycobacterium setense] |
| **63** | 764943667 | cutinase [Mycobacterium septicum] |
| **64** | 433300724 | Cutinase [Mycobacterium smegmatis JS623] |
| **64** | 489980598 | MULTISPECIES: cutinase [Mycobacterium] |
| **64** | 500106915 | cutinase [Mycobacterium vanbaalenii] |
| **64** | 518341035 | MULTISPECIES: hypothetical protein [Mycobacterium] |
| **64** | 518948436 | hypothetical protein [Mycobacterium sp. 360MFTsu5.1] |
| **64** | 633835831 | cutinase [Mycobacterium farcinogenes] |
| **64** | 639002929 | MULTISPECIES: cutinase [Mycobacterium] |
| **64** | 642750565 | cutinase [Mycobacterium neoaurum] |
| **64** | 656077549 | cutinase [Mycobacterium sp. URHD0025] |
| **64** | 656086715 | cutinase [Mycobacterium sp. URHB0044] |
| **64** | 656087741 | cutinase [Mycobacterium sp. UNC410CL29Cvi84] |
| **64** | 657266592 | cutinase [Mycobacterium sp. UM_WWY] |
| **64** | 738425771 | cutinase [Mycobacterium austroafricanum] |
| **64** | 738469972 | cutinase [Mycobacterium sp. 360MFTsu5.1] |
| **64** | 738493553 | cutinase [Mycobacterium vulneris] |
| **64** | 738512721 | cutinase [Mycobacterium sp. 141] |
| **64** | 738514756 | cutinase [Mycobacterium sp. UNCCL9] |
| **64** | 746278579 | cutinase [Mycobacterium setense] |
| **64** | 758330550 | cutinase [Mycobacterium llatzerense] |
| **64** | 764948457 | cutinase [Mycobacterium septicum] |
| **64** | 857808793 | cutinase [Mycobacterium sp. GPK 1020] |
| **65** | 442584538 | cutinase [Mycobacterium liflandii 128FXT] |
| **65** | 518565378 | cutinase [Mycobacterium avium] |
| **65** | 638967519 | cutinase [Mycobacterium sp. UM_Kg27] |
| **65** | 806834406 | cutinase [Mycobacterium nebraskense] |
| **65** | 806841186 | cutinase [Mycobacterium arupense] |
| **65** | 808802113 | cutinase [Mycobacterium sp. UM_NZ2] |
| **65** | 809005229 | cutinase [Mycobacterium sp. UM_Kg17] |
| **65** | 809066717 | cutinase [Mycobacterium sp. UM_Kg1] |
| **65** | 829094317 | cutinase [Mycobacterium haemophilum] |
| **65** | 829158745 | cutinase [Mycobacterium heraklionense] |
| **66** | 392064669 | putative cutinase cut3 [Mycobacterium abscessus 4S-0303] |
| **66** | 758526297 | serine esterase [Mycobacterium immunogenum] |
| **66** | 763235288 | serine esterase [Mycobacterium abscessus] |
| **66** | 808097621 | serine esterase [Mycobacterium chelonae] |
| **67** | 491197328 | cutinase [Mycobacterium abscessus] |
| **67** | 515399194 | cutinase [Mycobacterium abscessus] |
| **67** | 576468910 | cutinase family protein [Mycobacterium abscessus 1948] |
| **67** | 662145746 | cutinase [Mycobacterium abscessus] |
| **67** | 759017572 | cutinase [Mycobacterium immunogenum] |
| **67** | 808097211 | cutinase [Mycobacterium chelonae] |
| **68** | 151362571 | cutinase [Kineococcus radiotolerans SRS30216 = ATCC BAA-149] |
| **68** | 296022763 | cutinase [Cellulomonas flavigena DSM 20109] |
| **68** | 697886498 | hypothetical protein Q760_05675 [Cellulomonas cellasea DSM 20118] |
| **68** | 697974255 | cutinase [Cellulomonas bogoriensis 69B4 = DSM 16987] |
| **68** | 697974256 | cutinase [Cellulomonas bogoriensis 69B4 = DSM 16987] |
| **69** | 491281951 | cutinase [Mycobacterium rhodesiae] |
| **69** | 738388949 | cutinase [Mycobacterium aromaticivorans] |
| **69** | 829461880 | cutinase [Mycobacterium sp. EPa45] |
| **70** | 490016743 | cutinase [Mycobacterium xenopi] |
| **70** | 576413135 | cutinase family protein [Mycobacterium xenopi 4042] |
| **70** | 779921133 | cutinase, partial [Mycobacterium kyorinense] |
| **71** | 119955270 | Cutinase [Mycobacterium vanbaalenii PYR-1] |
| **71** | 145218027 | cutinase [Mycobacterium gilvum PYR-GCK] |
| **71** | 433295688 | Cutinase [Mycobacterium smegmatis JS623] |
| **71** | 490708285 | MULTISPECIES: cutinase [Mycobacterium] |
| **71** | 491281959 | cutinase [Mycobacterium rhodesiae] |
| **71** | 500047003 | cutinase [Mycobacterium smegmatis] |
| **71** | 503975085 | cutinase [Mycobacterium rhodesiae] |
| **71** | 504627238 | cutinase [Mycobacterium chubuense] |
| **71** | 517430829 | cutinase [Mycobacterium sp. 155] |
| **71** | 518342144 | MULTISPECIES: cutinase [Mycobacterium] |
| **71** | 635709134 | cutinase [Mycobacterium aromaticivorans JS19b1 = JCM 16368] |
| **71** | 638983423 | cutinase [Mycobacterium iranicum] |
| **71** | 638994003 | MULTISPECIES: cutinase [Mycobacterium] |
| **71** | 656074160 | MULTISPECIES: cutinase [Mycobacterium] |
| **71** | 656075884 | cutinase [Mycobacterium sp. URHD0025] |
| **71** | 656084830 | cutinase [Mycobacterium sp. URHB0044] |
| **71** | 657264655 | cutinase [Mycobacterium sp. UM_WWY] |
| **71** | 662770113 | cutinase [Mycobacterium neoaurum] |
| **71** | 738437771 | cutinase [Mycobacterium farcinogenes] |
| **71** | 738478758 | cutinase [Mycobacterium mageritense] |
| **71** | 738490956 | cutinase [Mycobacterium vulneris] |
| **71** | 746269147 | cutinase [Mycobacterium setense] |
| **71** | 750241427 | cutinase [Mycobacterium tusciae] |
| **71** | 750243882 | cutinase [Mycobacterium vaccae] |
| **71** | 759689204 | cutinase [Mycobacterium rufum] |
| **71** | 763104342 | cutinase [Mycobacterium llatzerense] |
| **71** | 764945763 | cutinase [Mycobacterium septicum] |
| **71** | 810953344 | cutinase [Mycobacterium obuense] |
| **71** | 860419231 | cutinase [Mycobacterium chubuense] |
| **72** | 620038265 | cutinase [Mycobacterium triplex] |
| **72** | 647293055 | cutinase [Mycobacterium genavense] |
| **72** | 764939394 | cutinase [Mycobacterium simiae] |
| **72** | 806820005 | cutinase [Mycobacterium lentiflavum] |
| **73** | 41399168 | hypothetical protein MAP_4236c [Mycobacterium avium subsp. paratuberculosis K-10] |
| **73** | 494294752 | cutinase [Mycobacterium parascrofulaceum] |
| **73** | 648290051 | MULTISPECIES: cutinase [Mycobacterium] |
| **73** | 750349052 | cutinase [Mycobacterium colombiense] |
| **73** | 808659800 | cutinase [Mycobacterium europaeum] |
| **73** | 829166289 | cutinase [Mycobacterium nebraskense] |
| **74** | 620040932 | serine esterase cutinase [Mycobacterium triplex] |
| **74** | 764936635 | cutinase [Mycobacterium simiae] |
| **74** | 806822969 | serine esterase cutinase [Mycobacterium lentiflavum] |
| **75** | 521090090 | cutinase [Amycolatopsis sp. ATCC 39116] |
| **75** | 568605465 | cutinase family protein [uncultured bacterium] |
| **75** | 654462031 | cutinase [Amycolatopsis thermoflava] |
| **75** | 703479632 | cutinase [Saccharothrix syringae] |
| **75** | 766802289 | cutinase [Amycolatopsis orientalis] |
| **76** | 549075575 | hypothetical protein NCAST_32_04700 [Nocardia asteroides NBRC 15531] |
| **76** | 750201388 | cutinase, partial [Leucobacter chromiiresistens] |
| **76** | 755632917 | cutinase [Leucobacter komagatae] |
| **76** | 815725744 | hypothetical protein [Leucobacter sp. Ag1] |
| **77** | 493287934 | cutinase [Mycobacterium tusciae] |
| **77** | 503980186 | cutinase [Mycobacterium rhodesiae] |
| **78** | 625016972 | hypothetical protein K875_01241 [Mycobacterium tuberculosis TKK-01-0051] |
| **78** | 738518359 | cutinase [Mycobacterium triplex] |
| **78** | 750349054 | cutinase [Mycobacterium colombiense] |
| **78** | 806820004 | Cutinase [Mycobacterium lentiflavum] |
| **78** | 829166291 | cutinase [Mycobacterium nebraskense] |
| **79** | 576459082 | cutinase family protein [Mycobacterium xenopi 3993] |
| **79** | 779921079 | cutinase [Mycobacterium kyorinense] |
| **80** | 500059198 | cutinase [Mycobacterium ulcerans] |
| **80** | 523654645 | serine esterase cutinase family [Mycobacterium sp. 012931] |
| **80** | 576420534 | cutinase family protein [Mycobacterium kansasii 732] |
| **80** | 576499485 | cutinase family protein [Mycobacterium ulcerans str. Harvey] |
| **80** | 738439735 | cutinase [Mycobacterium kansasii] |
| **80** | 738463439 | cutinase [Mycobacterium gastri] |
| **81** | 289422114 | cutinase precursor cut3 [Mycobacterium tuberculosis CPHL_A] |
| **81** | 489513495 | MULTISPECIES: cutinase [Mycobacterium tuberculosis complex] |
| **81** | 505104363 | cutinase precursor Cut3 [Mycobacterium canettii] |
| **81** | 829094088 | cutinase [Mycobacterium haemophilum] |
| **82** | 407371952 | cutinase family protein [Mycobacterium hassiacum DSM 44199] |
| **82** | 750351165 | cutinase [Mycobacterium phlei] |
| **82** | 820789233 | cutinase [Mycobacterium elephantis] |
| **82** | 857807598 | Cutinase [Mycobacterium sp. GPK 1020] |
| **83** | 489990787 | cutinase family serine esterase [Mycobacterium smegmatis] |
| **83** | 489992764 | cutinase family serine esterase [Mycobacterium smegmatis] |
| **83** | 491290181 | cutinase [Mycobacterium rhodesiae] |
| **83** | 500049208 | cutinase [Mycobacterium smegmatis] |
| **83** | 505121531 | lipase essential for disintegration of autophagic bodies inside the vacuole [Mycobacterium smegmatis] |
| **83** | 517431522 | cutinase [Mycobacterium sp. 155] |
| **83** | 518175780 | MULTISPECIES: cutinase [Mycobacterium] |
| **83** | 518343726 | MULTISPECIES: cutinase [Mycobacterium] |
| **83** | 518814329 | cutinase [Mycobacterium sp. 141] |
| **83** | 639004926 | MULTISPECIES: cutinase [Mycobacterium] |
| **83** | 639007697 | MULTISPECIES: cutinase [Mycobacterium] |
| **83** | 656073625 | MULTISPECIES: cutinase [Mycobacterium] |
| **83** | 656078541 | cutinase [Mycobacterium sp. URHD0025] |
| **83** | 738393445 | cutinase [Mycobacterium aromaticivorans] |
| **83** | 738475951 | cutinase [Mycobacterium sp. UM_WWY] |
| **83** | 738493504 | cutinase [Mycobacterium mageritense] |
| **83** | 738500375 | cutinase [Mycobacterium vulneris] |
| **83** | 740779377 | MULTISPECIES: cutinase [Mycobacterium] |
| **83** | 746274273 | cutinase [Mycobacterium setense] |
| **83** | 763103891 | cutinase [Mycobacterium llatzerense] |
| **83** | 764952984 | cutinase [Mycobacterium septicum] |
| **83** | 810950502 | cutinase [Mycobacterium obuense] |
| **83** | 829463403 | cutinase [Mycobacterium sp. EPa45] |
| **84** | 518344585 | MULTISPECIES: hypothetical protein [Mycobacterium] |
| **84** | 662771343 | cutinase [Mycobacterium neoaurum] |
| **84** | 763108057 | cutinase [Mycobacterium llatzerense] |
| **84** | 806842518 | cutinase [Mycobacterium arupense] |
| **84** | 810942823 | cutinase [Mycobacterium obuense] |
| **85** | 268310716 | cutinase [Thermomonospora curvata DSM 43183] |
| **85** | 502614953 | cutinase [Thermomonospora curvata] |
| **86** | 583001639 | serine esterase, cutinase family [Actinokineospora spheciospongiae] |
| **86** | 750595267 | cutinase [Streptomyces scabrisporus] |
| **87** | 492006927 | hypothetical protein L083_4737 [Actinoplanes sp. N902-109] |
| **87** | 556035192 | hypothetical protein AFR_20835 [Actinoplanes friuliensis DSM 7358] |
| **87** | 703060160 | hypothetical protein [Catenuloplanes japonicus] |
| **88** | 632917113 | cutinase 1 [Ustilaginoidea virens] |
| **88** | 781084024 | hypothetical protein UVI_046280 [Ustilaginoidea virens] |
| **89** | 343427889 | conserved hypothetical protein [Sporisorium reilianum SRZ2] |
| **89** | 388857390 | uncharacterized protein UHOR_08679 [Ustilago hordei] |
| **89** | 573027082 | hypothetical protein PaG_05532 [Pseudozyma aphidis DSM 70725] |
| **89** | 673530464 | cutinase family protein [Melanopsichium pennsylvanicum 4] |
| **89** | 674217147 | cutinase [Pseudozyma antarctica] |
| **89** | 758987421 | hypothetical protein UMAG_11211 [Ustilago maydis 521] |
| **89** | 808364652 | cutinase [Pseudozyma hubeiensis SY62] |
| **89** | 858044272 | hypothetical protein [Sporisorium scitamineum] |
| **90** | 517430830 | hypothetical protein [Mycobacterium sp. 155] |
| **90** | 518177479 | MULTISPECIES: hypothetical protein [Mycobacterium] |
| **90** | 518816295 | hypothetical protein [Mycobacterium sp. 141] |
| **90** | 656074159 | cutinase [Mycobacterium sp. 360MFTsu5.1] |
| **90** | 657264652 | cutinase [Mycobacterium sp. UM_WWY] |
| **90** | 738478760 | cutinase [Mycobacterium mageritense] |
| **90** | 738490959 | cutinase [Mycobacterium vulneris] |
| **90** | 738526717 | cutinase [Mycobacterium sp. URHD0025] |
| **90** | 746269144 | cutinase [Mycobacterium setense] |
| **90** | 759684933 | cutinase [Mycobacterium sp. UNC280MFTsu5.1] |
| **90** | 764945761 | cutinase [Mycobacterium septicum] |
| **91** | 295896598 | Cutinase [Mycobacterium parascrofulaceum ATCC BAA-614] |
| **91** | 489970203 | cutinase [Mycobacterium avium] |
| **91** | 504193974 | cutinase [Mycobacterium intracellulare] |
| **91** | 523910942 | serine esterase cutinase [Mycobacterium yongonense] |
| **91** | 564204152 | cutinase [Mycobacterium avium 11-0986] |
| **91** | 576447478 | cutinase family protein [Mycobacterium avium subsp. avium 2285 (R)] |
| **91** | 576459449 | cutinase family protein [Mycobacterium intracellulare 1956] |
| **91** | 620038266 | serine esterase, cutinase family protein [Mycobacterium triplex] |
| **91** | 750349206 | cutinase [Mycobacterium colombiense] |
| **91** | 764939395 | cutinase [Mycobacterium simiae] |
| **91** | 764976127 | cutinase [Mycobacterium avium] |
| **91** | 806820006 | serine esterase, cutinase family protein [Mycobacterium lentiflavum] |
| **91** | 806839109 | cutinase [Mycobacterium nebraskense] |
| **91** | 808575761 | serine esterase, cutinase [Mycobacterium bohemicum DSM 44277] |
| **91** | 808659801 | serine esterase, cutinase family protein [Mycobacterium europaeum] |
| **92** | 295896443 | Cutinase [Mycobacterium parascrofulaceum ATCC BAA-614] |
| **92** | 41398357 | hypothetical protein MAP_3428c [Mycobacterium avium subsp. paratuberculosis K-10] |
| **92** | 504193910 | MULTISPECIES: hypothetical protein [Mycobacterium] |
| **92** | 576434621 | cutinase family protein [Mycobacterium avium subsp. avium 2285 (S)] |
| **92** | 625017088 | hypothetical protein K875_01357 [Mycobacterium tuberculosis TKK-01-0051] |
| **92** | 738445246 | cutinase [Mycobacterium cosmeticum] |
| **92** | 738518671 | cutinase [Mycobacterium triplex] |
| **92** | 738519952 | cutinase [Mycobacterium genavense] |
| **92** | 764942401 | cutinase [Mycobacterium simiae] |
| **92** | 806820150 | cutinase Cut3 [Mycobacterium lentiflavum] |
| **92** | 808659930 | cutinase Cut3 [Mycobacterium europaeum] |
| **92** | 829164951 | cutinase [Mycobacterium nebraskense] |
| **93** | 490026458 | cutinase [Mycobacterium vaccae] |
| **93** | 738422454 | cutinase [Mycobacterium austroafricanum] |
| **93** | 752637152 | cutinase [Mycobacterium vanbaalenii] |
| **94** | 493590637 | cutinase [Frankia sp. EUN1f] |
| **94** | 494773459 | cutinase [Frankia sp. CN3] |
| **94** | 503187102 | None |
| **94** | 517325045 | hypothetical protein [Frankia sp. BCU110501] |
| **94** | 648619273 | cutinase [Frankia sp. BMG5.12] |
| **95** | 752274904 | hypothetical protein ANO11243_076330 [fungal sp. No.11243] |
| **95** | 752277218 | hypothetical protein ANO11243_061480 [fungal sp. No.11243] |
| **96** | 169857640 | hypothetical protein CC1G_05430 [Coprinopsis cinerea okayama7#130] |
| **96** | 359835666 | hypothetical protein ACPL_3212 [Actinoplanes sp. SE50/110] |
| **96** | 557947610 | cutinase precursor [Leucoagaricus gongylophorus] |
| **96** | 648168820 | hypothetical protein GALMADRAFT_275674 [Galerina marginata CBS 339.88] |
| **96** | 648168829 | hypothetical protein GALMADRAFT_87819 [Galerina marginata CBS 339.88] |
| **96** | 749851431 | carbohydrate esterase family 5 protein [Sphaerobolus stellatus SS14] |
| **96** | 749858257 | carbohydrate esterase family 5 protein [Sphaerobolus stellatus SS14] |
| **96** | 749858258 | carbohydrate esterase family 5 protein [Sphaerobolus stellatus SS14] |
| **97** | 531862081 | carbohydrate esterase family 5 protein [Ophiocordyceps sinensis CO18] |
| **97** | 799243572 | hypothetical protein HIM_08068 [Hirsutella minnesotensis 3608] |
| **98** | 125656332 | putative cutinase [Trichoderma harzianum] |
| **98** | 358388326 | carbohydrate esterase family 5 protein [Trichoderma virens Gv29-8] |
| **98** | 358392577 | carbohydrate esterase family 5 protein [Trichoderma atroviride IMI 206040] |
| **98** | 573987111 | cutinase precursor, putative [Cordyceps militaris CM01] |
| **98** | 629686455 | putative cutinase [Metarhizium acridum CQMa 102] |
| **98** | 629725673 | Cutinase, monofunctional [Metarhizium robertsii ARSEF 23] |
| **98** | 667658913 | cutinase precursor [Beauveria bassiana ARSEF 2860] |
| **98** | 672795110 | Cutinase-like protein [Acremonium chrysogenum ATCC 11550] |
| **98** | 685425699 | Chain A, Structure Of Cutinase From Trichoderma Reesei In Its Native Form. |
| **98** | 729182953 | hypothetical protein VHEMI07569 [Torrubiella hemipterigena] |
| **98** | 734656748 | Cutinase [Metarhizium album ARSEF 1941] |
| **98** | 743636491 | Cutinase, partial [Metarhizium brunneum ARSEF 3297] |
| **98** | 818156094 | cutinase [Trichoderma harzianum] |
| **99** | 407925366 | Cutinase [Macrophomina phaseolina MS6] |
| **99** | 407927716 | Cutinase [Macrophomina phaseolina MS6] |
| **99** | 615417003 | putative cutinase protein [Neofusicoccum parvum UCRNP2] |
| **99** | 615430070 | putative cutinase protein [Neofusicoccum parvum UCRNP2] |
| **99** | 821060986 | hypothetical protein UCDDS831_g06950 [Diplodia seriata] |
| **99** | 821065743 | putative cutinase family protein [Diplodia seriata] |
| **100** | 453089277 | carbohydrate esterase family 5 protein [Sphaerulina musiva SO2202] |
| **100** | 662530319 | cutinase-domain-containing protein [Aureobasidium pullulans EXF-150] |
| **100** | 662543307 | carbohydrate esterase family 5 protein [Aureobasidium subglaciale EXF-2481] |
| **100** | 796709931 | hypothetical protein TI39_contig307g00020 [Zymoseptoria brevis] |
| **101** | 407918291 | Cutinase [Macrophomina phaseolina MS6] |
| **101** | 407920373 | hypothetical protein MPH_09279 [Macrophomina phaseolina MS6] |
| **101** | 615402414 | putative cutinase protein [Neofusicoccum parvum UCRNP2] |
| **101** | 615414033 | putative cutinase protein [Neofusicoccum parvum UCRNP2] |
| **101** | 821070545 | putative cutinase precursor [Diplodia seriata] |
| **101** | 821074065 | putative cutinase precursor [Diplodia seriata] |
| **102** | 325184956 | predicted protein putative [Albugo laibachii Nc14] |
| **102** | 635367507 | unnamed protein product [Albugo candida] |
| **102** | 635367508 | unnamed protein product [Albugo candida] |
| **103** | 518947188 | hypothetical protein [Mycobacterium sp. 360MFTsu5.1] |
| **103** | 738487101 | serine esterase, partial [Mycobacterium sp. URHB0044] |
| **103** | 759675684 | MULTISPECIES: serine esterase [Mycobacterium] |
| **104** | 517432172 | hypothetical protein [Mycobacterium sp. 155] |
| **104** | 648665336 | cutinase [Mycobacterium sp. 141] |
| **104** | 738474014 | cutinase [Mycobacterium sp. UM_WWY] |
| **105** | 738470206 | cutinase [Mycobacterium sp. 360MFTsu5.1] |
| **105** | 763104343 | cutinase [Mycobacterium llatzerense] |
| **106** | 491281956 | cutinase [Mycobacterium rhodesiae] |
| **106** | 635709135 | cutinase [Mycobacterium aromaticivorans JS19b1 = JCM 16368] |
| **106** | 829463665 | cutinase [Mycobacterium sp. EPa45] |
| **107** | 119955269 | cutinase [Mycobacterium vanbaalenii PYR-1] |
| **107** | 390614705 | Cutinase [Mycobacterium chubuense NBB4] |
| **107** | 399230473 | Cutinase Cut4 [Mycobacterium smegmatis str. MC2 155] |
| **107** | 490023457 | cutinase [Mycobacterium thermoresistibile] |
| **107** | 499877795 | cutinase [Mycobacterium sp. KMS] |
| **107** | 500225703 | cutinase [Mycobacterium gilvum] |
| **107** | 597300995 | serine esterase, cutinase [Mycobacterium cosmeticum] |
| **107** | 638983420 | cutinase [Mycobacterium iranicum] |
| **107** | 642749772 | serine esterase, cutinase [Mycobacterium neoaurum] |
| **107** | 674843752 | MULTISPECIES: cutinase [Mycobacterium] |
| **107** | 759698724 | cutinase [Mycobacterium rufum] |
| **107** | 859090507 | Cutinase [Mycobacterium obuense] |
| **107** | 859093003 | Cutinase [Mycobacterium chlorophenolicum] |
| **108** | 29838408 | cutinase [Phytophthora brassicae] |
| **108** | 301102243 | cutinase, putative [Phytophthora infestans T30-4] |
| **108** | 301102398 | cutinase, putative [Phytophthora infestans T30-4] |
| **108** | 566028883 | hypothetical protein F443_04909 [Phytophthora parasitica P1569] |
| **108** | 566031981 | hypothetical protein F443_02353 [Phytophthora parasitica P1569] |
| **108** | 566032254 | hypothetical protein F443_02142 [Phytophthora parasitica P1569] |
| **108** | 566032255 | hypothetical protein F443_02143 [Phytophthora parasitica P1569] |
| **108** | 566032256 | hypothetical protein F443_02144 [Phytophthora parasitica P1569] |
| **108** | 567966235 | hypothetical protein L915_04776 [Phytophthora parasitica] |
| **108** | 66270075 | cutinase [Phytophthora infestans] |
| **108** | 675181403 | hypothetical protein PPTG_07182 [Phytophthora parasitica INRA-310] |
| **108** | 695430249 | cutin hydrolase [Phytophthora sojae] |
| **108** | 695436778 | cutin hydrolase [Phytophthora sojae] |
| **108** | 695436782 | cutin hydrolase [Phytophthora sojae] |
| **108** | 695436786 | cutin hydrolase [Phytophthora sojae] |
| **108** | 695436789 | cutin hydrolase [Phytophthora sojae] |
| **108** | 695436797 | hypothetical protein PHYSODRAFT_336225 [Phytophthora sojae] |
| **108** | 695436802 | hypothetical protein PHYSODRAFT_517467 [Phytophthora sojae] |
| **108** | 695436805 | cutin hydrolase [Phytophthora sojae] |
| **108** | 695436821 | cutin hydrolase [Phytophthora sojae] |
| **108** | 695436825 | cutin hydrolase [Phytophthora sojae] |
| **109** | 489179477 | esterase [Amycolatopsis vancoresmycina] |
| **109** | 490093089 | cutinase [Streptomyces viridochromogenes] |
| **109** | 490699131 | esterase [Amycolatopsis vancoresmycina] |
| **109** | 491617733 | esterase [Streptomyces bottropensis] |
| **109** | 493426163 | cutinase [Streptomyces turgidiscabies] |
| **109** | 497723924 | cutinase [Streptomyces chartreusis] |
| **109** | 502770323 | cutinase [Streptomyces scabiei] |
| **109** | 518964274 | cutinase [Streptomyces canus] |
| **109** | 518969991 | cutinase [Streptomyces sp. 303MFCol5.2] |
| **109** | 519333552 | cutinase [Streptomyces afghaniensis] |
| **109** | 522135636 | hypothetical protein [Amycolatopsis balhimycina] |
| **109** | 637494906 | cutinase [Amycolatopsis rifamycinica] |
| **109** | 648459531 | cutinase [Streptomyces prunicolor] |
| **109** | 648486901 | cutinase [Streptomyces sp. R1-NS-10] |
| **109** | 655410948 | cutinase [Streptomyces sp. 351MFTsu5.1] |
| **109** | 663207235 | MULTISPECIES: cutinase [Streptomyces] |
| **109** | 663325281 | cutinase [Streptomyces sp. NRRL B-3229] |
| **109** | 664073505 | cutinase [Streptomyces fulvoviolaceus] |
| **109** | 664128658 | cutinase [Streptomyces cellulosae] |
| **109** | 664318667 | cutinase [Streptomyces sp. NRRL S-475] |
| **109** | 664405008 | cutinase [Streptomyces violaceoruber] |
| **109** | 664425225 | cutinase [Streptomyces sp. NRRL S-646] |
| **109** | 664535216 | cutinase [Streptomyces sp. NRRL WC-3774] |
| **109** | 664611276 | MULTISPECIES: cutinase [Streptomyces] |
| **109** | 665555123 | MULTISPECIES: cutinase [Streptomyces] |
| **109** | 702832769 | cutinase [Streptomyces sp. NRRL F-525] |
| **109** | 703236303 | cutinase [Kibdelosporangium aridum] |
| **109** | 716918490 | cutinase [Streptomyces galbus] |
| **109** | 739396081 | cutinase [Rhodococcus rhodnii] |
| **109** | 739825985 | cutinase [Streptomyces griseus] |
| **109** | 739854196 | cutinase [Streptomyces scabiei] |
| **109** | 739858230 | cutinase [Streptomyces mirabilis] |
| **109** | 739956342 | cutinase [Streptomyces sp. NRRL F-3213] |
| **109** | 759985044 | cutinase [Streptomyces xylophagus] |
| **109** | 780338139 | cutinase [Streptomyces sp. FxanaA7] |
| **109** | 818928182 | cutinase [Streptomyces europaeiscabiei] |
| **109** | 820273781 | cutinase [Streptomyces sp. MUSC119T] |
| **110** | 154277416 | predicted protein [Histoplasma capsulatum NAm1] |
| **110** | 225561142 | conserved hypothetical protein [Histoplasma capsulatum G186AR] |
| **110** | 239614748 | Axe2 [Blastomyces dermatitidis ER-3] |
| **110** | 240280301 | conserved hypothetical protein [Histoplasma capsulatum H143] |
| **110** | 824375628 | hypothetical protein EMPG_11657 [Emmonsia parva UAMH 139] |
| **111** | 752275688 | hypothetical protein ANO11243_074960 [fungal sp. No.11243] |
| **111** | 752277209 | hypothetical protein ANO11243_061390 [fungal sp. No.11243] |
| **111** | 752282145 | hypothetical protein ANO11243_002680 [fungal sp. No.11243] |
| **111** | 752282826 | hypothetical protein ANO11243_009560 [fungal sp. No.11243] |
| **112** | 477533229 | cutinase family protein [Colletotrichum orbiculare MAFF 240422] |
| **112** | 530462294 | cutinase [Colletotrichum gloeosporioides Cg-14] |
| **112** | 596710333 | cutinase [Colletotrichum gloeosporioides Nara gc5] |
| **112** | 615478420 | cutinase [Colletotrichum fioriniae PJ7] |
| **112** | 630010435 | hypothetical protein PFICI_02431 [Pestalotiopsis fici W106-1] |
| **112** | 640923941 | putative cutinase [Colletotrichum sublineola] |
| **112** | 827075870 | cutinase [Colletotrichum graminicola M1.001] |
| **113** | 169605001 | hypothetical protein SNOG_05516 [Phaeosphaeria nodorum SN15] |
| **113** | 330923965 | hypothetical protein PTT_11697 [Pyrenophora teres f. teres 0-1] |
| **113** | 396469340 | hypothetical protein LEMA_P113170.1 [Leptosphaeria maculans JN3] |
| **113** | 628067523 | carbohydrate esterase family 5 protein [Bipolaris sorokiniana ND90Pr] |
| **113** | 628221722 | carbohydrate esterase family 5 protein [Bipolaris zeicola 26-R-13] |
| **114** | 353240080 | hypothetical protein PIIN_05900 [Piriformospora indica DSM 11827] |
| **114** | 353245104 | hypothetical protein PIIN_10180 [Piriformospora indica DSM 11827] |
| **114** | 660960877 | cutinase [Rhizoctonia solani 123E] |
| **114** | 751671852 | carbohydrate esterase family 5 protein [Serendipita vermifera MAFF 305830] |
| **114** | 751835379 | hypothetical protein RSOLAG1IB_07369 [Rhizoctonia solani AG-1 IB] |
| **115** | 471870100 | cutinase [Rhizoctonia solani AG-1 IB] |
| **115** | 471870101 | cutinase [Rhizoctonia solani AG-1 IB] |
| **115** | 471881575 | cutinase [Rhizoctonia solani AG-1 IB] |
| **115** | 576992519 | cutinase [Rhizoctonia solani AG-3 Rhs1AP] |
| **115** | 751841714 | putative cutinase Rv1984c/MT2037 OS=Mycobacterium tuberculosis GN=Rv1984c PE=3 SV=1 [Rhizoctonia solani AG-1 IB] |
| **116** | 471904197 | Cutinase [Rhizoctonia solani AG-1 IB] |
| **116** | 576989850 | cutinase [Rhizoctonia solani AG-3 Rhs1AP] |
| **116** | 639564772 | hypothetical protein RSAG8_06851, partial [Rhizoctonia solani AG-8 WAC10335] |
| **117** | 403655696 | cutinase [Mycobacterium fortuitum subsp. fortuitum DSM 46621] |
| **117** | 489989858 | cutinase [Mycobacterium smegmatis] |
| **117** | 657264654 | cutinase [Mycobacterium sp. UM_WWY] |
| **117** | 738438857 | cutinase [Mycobacterium farcinogenes] |
| **117** | 738478759 | cutinase [Mycobacterium mageritense] |
| **117** | 738496753 | cutinase [Mycobacterium vulneris] |
| **117** | 738526900 | cutinase [Mycobacterium sp. URHD0025] |
| **117** | 746333072 | cutinase [Mycobacterium setense] |
| **117** | 764948747 | cutinase [Mycobacterium septicum] |
| **118** | 403661001 | hypothetical protein MFORT_04181 [Mycobacterium fortuitum subsp. fortuitum DSM 46621] |
| **118** | 602520170 | cutinase Cut3 [Mycobacterium vulneris] |
| **118** | 633836632 | cutinase Cut3 [Mycobacterium farcinogenes] |
| **118** | 656079107 | cutinase [Mycobacterium sp. URHD0025] |
| **118** | 764951489 | cutinase [Mycobacterium septicum] |
| **118** | 808668834 | cutinase Cut3 [Mycobacterium conceptionense] |
| **119** | 738633727 | cutinase [Nocardia cyriacigeorgica] |
| **119** | 750460673 | cutinase [Nocardia tenerifensis] |
| **119** | 759976942 | cutinase [Nocardia thailandica] |
| **120** | 477592106 | carbohydrate esterase family 5 protein [Bipolaris maydis ATCC 48331] |
| **120** | 578495481 | carbohydrate esterase family 5 protein [Bipolaris victoriae FI3] |
| **120** | 627819081 | carbohydrate esterase family 5 protein [Bipolaris oryzae ATCC 44560] |
| **120** | 628087040 | carbohydrate esterase family 5 protein [Bipolaris sorokiniana ND90Pr] |
| **120** | 636581129 | carbohydrate esterase family 5 protein [Setosphaeria turcica Et28A] |
| **121** | 615472313 | hypothetical protein CFIO01_04188 [Colletotrichum fioriniae PJ7] |
| **121** | 666403721 | hypothetical protein S7711_08133 [Stachybotrys chartarum IBT 7711] |
| **121** | 667728730 | hypothetical protein S40285_04063 [Stachybotrys chlorohalonata IBT 40285] |
| **121** | 672792849 | cutinase-like protein [Acremonium chrysogenum ATCC 11550] |
| **122** | 145241506 | hypothetical protein ANI_1_996084 [Aspergillus niger CBS 513.88] |
| **122** | 154308307 | hypothetical protein BC1G_07899 [Botrytis cinerea B05.10] |
| **122** | 347827317 | carbohydrate esterase family 5 protein [Botrytis cinerea T4] |
| **122** | 350639811 | hypothetical protein ASPNIDRAFT_188942 [Aspergillus niger ATCC 1015] |
| **122** | 358373643 | hypothetical protein AKAW_08354 [Aspergillus kawachii IFO 4308] |
| **122** | 662508256 | acetylxylan esterase precursor [Aureobasidium melanogenum CBS 110374] |
| **123** | 630028590 | hypothetical protein PFICI_09769 [Pestalotiopsis fici W106-1] |
| **123** | 630033329 | hypothetical protein PFICI_11443 [Pestalotiopsis fici W106-1] |
| **124** | 389627108 | cutinase [Magnaporthe oryzae 70-15] |
| **124** | 630012043 | hypothetical protein PFICI_03235 [Pestalotiopsis fici W106-1] |
| **124** | 685409557 | cutinase [Gaeumannomyces graminis var. tritici R3-111a-1] |
| **124** | 835901034 | cutinase [Magnaporthiopsis poae ATCC 64411] |
| **125** | 568445934 | hypothetical protein AGABI2DRAFT_176382 [Agaricus bisporus var. bisporus H97] |
| **125** | 763725213 | carbohydrate esterase family 5 protein [Hypholoma sublateritium FD-334 SS-4] |
| **126** | 115383710 | predicted protein [Aspergillus terreus NIH2624] |
| **126** | 119481743 | cutinase family protein [Neosartorya fischeri NRRL 181] |
| **126** | 145231462 | cutinase [Aspergillus niger CBS 513.88] |
| **126** | 159129830 | cutinase, putative [Aspergillus fumigatus A1163] |
| **126** | 238490254 | cutinase, putative [Aspergillus flavus NRRL3357] |
| **126** | 255936417 | Pc13g08100 [Penicillium rubens Wisconsin 54-1255] |
| **126** | 259486235 | TPA: cutinase, putative (AFU_orthologue; AFUA_2G14420) [Aspergillus nidulans FGSC A4] |
| **126** | 317146439 | cutinase [Aspergillus oryzae RIB40] |
| **126** | 350634234 | hypothetical protein ASPNIDRAFT_36638 [Aspergillus niger ATCC 1015] |
| **126** | 358376252 | hypothetical protein AKAW_10929 [Aspergillus kawachii IFO 4308] |
| **126** | 425765642 | hypothetical protein PDIP_88200 [Penicillium digitatum Pd1] |
| **126** | 525583715 | putative cutinase [Penicillium oxalicum 114-2] |
| **126** | 599157839 | cutinase-domain-containing protein [Aspergillus ruber CBS 135680] |
| **126** | 635514152 | hypothetical protein AO1008_04290 [Aspergillus oryzae 100-8] |
| **126** | 67524751 | hypothetical protein AN2833.2 [Aspergillus nidulans FGSC A4] |
| **126** | 700445764 | Cutinase [Penicillium expansum] |
| **126** | 700491156 | Cutinase [Penicillium italicum] |
| **126** | 71002188 | cutinase [Aspergillus fumigatus Af293] |
| **126** | 768695561 | Cutinase [Penicillium solitum] |
| **126** | 768706972 | Cutinase [Aspergillus flavus AF70] |
| **126** | 802090976 | hypothetical protein T310_5227 [Rasamsonia emersonii CBS 393.64] |
| **126** | 816349280 | hypothetical protein AOCH_001615 [Aspergillus ochraceoroseus] |
| **126** | 83768507 | unnamed protein product [Aspergillus oryzae RIB40] |
| **126** | 846915806 | cutinase [Aspergillus fumigatus Z5] |
| **126** | 849270321 | cutinase [Neosartorya udagawae] |
| **126** | 859269571 | Putative Function: the Cutinase CutA from Botrytis cinerea catalyzes the hydrolysis of cutin (Precursor) [Penicillium brasilianum] |
| **127** | 490027018 | cutinase [Mycobacterium vaccae] |
| **127** | 500102692 | cutinase [Mycobacterium vanbaalenii] |
| **127** | 500225704 | cutinase [Mycobacterium gilvum] |
| **127** | 504627236 | cutinase [Mycobacterium chubuense] |
| **127** | 638983417 | cutinase [Mycobacterium iranicum] |
| **127** | 662770111 | MULTISPECIES: cutinase [Mycobacterium] |
| **127** | 759698721 | cutinase [Mycobacterium rufum] |
| **127** | 810953343 | cutinase [Mycobacterium obuense] |
| **127** | 821599595 | cutinase [Mycobacterium sp. UM_NYF] |
| **127** | 859093002 | Cutinase [Mycobacterium chlorophenolicum] |
| **127** | 859095981 | Cutinase [Mycobacterium chubuense] |
| **128** | 356478532 | cutinase [Mycobacterium thermoresistibile ATCC 19527] |
| **128** | 489983866 | cutinase [Mycobacterium phlei] |
| **128** | 499877794 | MULTISPECIES: cutinase [Mycobacterium] |
| **128** | 820789232 | cutinase [Mycobacterium elephantis] |
| **128** | 857807597 | cutinase [Mycobacterium sp. GPK 1020] |
| **128** | 858009309 | cutinase [Mycobacterium sp. WCM 7299] |
| **129** | 491281950 | cutinase [Mycobacterium rhodesiae] |
| **129** | 738388952 | cutinase [Mycobacterium aromaticivorans] |
| **129** | 829461881 | cutinase [Mycobacterium sp. EPa45] |
| **130** | 489978261 | MULTISPECIES: serine esterase [Mycobacterium] |
| **130** | 489990420 | cutinase [Mycobacterium smegmatis] |
| **130** | 500047429 | serine esterase [Mycobacterium smegmatis] |
| **130** | 517430248 | hypothetical protein [Mycobacterium sp. 155] |
| **130** | 518815645 | hypothetical protein [Mycobacterium sp. 141] |
| **130** | 602522428 | serine esterase, cutinase [Mycobacterium vulneris] |
| **130** | 738472560 | hypothetical protein [Mycobacterium sp. UM_WWY] |
| **130** | 738477814 | hypothetical protein [Mycobacterium mageritense] |
| **130** | 738526391 | serine esterase [Mycobacterium sp. URHD0025] |
| **130** | 746270018 | serine esterase [Mycobacterium setense] |
| **130** | 764946511 | serine esterase [Mycobacterium septicum] |
| **130** | 823282798 | MULTISPECIES: serine esterase [Mycobacterium] |
| **131** | 635708692 | serine esterase [Mycobacterium aromaticivorans JS19b1 = JCM 16368] |
| **131** | 738471181 | serine esterase [Mycobacterium rhodesiae] |
| **131** | 829459576 | hypothetical protein [Mycobacterium sp. EPa45] |
| **132** | 433295689 | Cutinase [Mycobacterium smegmatis JS623] |
| **132** | 517430828 | cutinase [Mycobacterium sp. 155] |
| **132** | 518816293 | cutinase [Mycobacterium sp. 141] |
| **132** | 602522988 | Cutinase [Mycobacterium vulneris] |
| **132** | 602543517 | Cutinase [Mycobacterium mageritense DSM 44476] |
| **132** | 633838894 | Cutinase [Mycobacterium farcinogenes] |
| **132** | 738473006 | cutinase [Mycobacterium sp. UM_WWY] |
| **132** | 738526720 | cutinase [Mycobacterium sp. URHD0025] |
| **132** | 746269149 | cutinase [Mycobacterium setense] |
| **132** | 808670296 | Cutinase [Mycobacterium conceptionense] |
| **132** | 858012815 | Cutinase [Mycobacterium fortuitum subsp. fortuitum DSM 46621] |
| **133** | 531860947 | carbohydrate esterase family 5 protein [Ophiocordyceps sinensis CO18] |
| **133** | 666407080 | hypothetical protein S7711_00988 [Stachybotrys chartarum IBT 7711] |
| **133** | 667723848 | hypothetical protein S40285_02411 [Stachybotrys chlorohalonata IBT 40285] |
| **133** | 672794044 | Acetylxylan esterase-like protein [Acremonium chrysogenum ATCC 11550] |
| **134** | 584126899 | hypothetical protein FVEG_00397 [Fusarium verticillioides 7600] |
| **134** | 835889887 | hypothetical protein MAPG_01224 [Magnaporthiopsis poae ATCC 64411] |
| **135** | 380490910 | cutinase-2 [Colletotrichum higginsianum] |
| **135** | 477535815 | cutinase precursor [Colletotrichum orbiculare MAFF 240422] |
| **135** | 615444260 | cutinase-2 [Colletotrichum fioriniae PJ7] |
| **136** | 407928205 | Cutinase [Macrophomina phaseolina MS6] |
| **136** | 615430615 | putative cutinase 1 protein [Neofusicoccum parvum UCRNP2] |
| **136** | 821067229 | putative cutinase 1 [Diplodia seriata] |
| **137** | 407922944 | Cutinase [Macrophomina phaseolina MS6] |
| **137** | 667839089 | hypothetical protein W97_07719 [Coniosporium apollinis CBS 100218] |
| **138** | 751698614 | carbohydrate esterase family 5 protein [Hebeloma cylindrosporum h7] |
| **138** | 751698620 | carbohydrate esterase family 5 protein [Hebeloma cylindrosporum h7] |
| **139** | 169850627 | triacylglycerol lipase [Coprinopsis cinerea okayama7#130] |
| **139** | 169850838 | cutinase [Coprinopsis cinerea okayama7#130] |
| **140** | 489506990 | MULTISPECIES: cutinase [Mycobacterium tuberculosis complex] |
| **140** | 493287454 | cutinase [Mycobacterium tusciae] |
| **140** | 503975086 | cutinase [Mycobacterium rhodesiae] |
| **140** | 505117989 | hypothetical protein [Mycobacterium smegmatis] |
| **140** | 638967343 | cutinase [Mycobacterium sp. UM_WGJ] |
| **140** | 656317449 | cutinase [Mycobacterium avium] |
| **140** | 752671028 | cutinase [Mycobacterium sinense] |
| **140** | 806842775 | MULTISPECIES: cutinase [Mycobacterium] |
| **140** | 808800901 | cutinase [Mycobacterium sp. UM_NZ2] |
| **140** | 809072872 | cutinase [Mycobacterium sp. UM_Kg1] |
| **140** | 829156169 | cutinase [Mycobacterium heraklionense] |
| **141** | 504797243 | cutinase [Nocardia brasiliensis] |
| **141** | 659852747 | cutinase [Nocardia brasiliensis] |
| **141** | 738618896 | cutinase [Nocardia sp. CNY236] |
| **141** | 748227122 | cutinase [Nocardia araoensis] |
| **141** | 750419597 | cutinase [Nocardia vinacea] |
| **141** | 750459800 | cutinase [Nocardia tenerifensis] |
| **141** | 750496033 | cutinase [Nocardia pneumoniae] |
| **141** | 754902859 | cutinase [Nocardia brasiliensis] |
| **141** | 760007006 | cutinase [Nocardia abscessus] |
| **141** | 760041890 | cutinase [Nocardia asiatica] |
| **142** | 549076082 | hypothetical protein NCAST_32_09760 [Nocardia asteroides NBRC 15531] |
| **142** | 750424853 | cutinase [Nocardia takedensis] |
| **142** | 759967293 | cutinase [Nocardia thailandica] |
| **143** | 629675334 | putative cutinase protein [Eutypa lata UCREL1] |
| **143** | 630009955 | hypothetical protein PFICI_02191 [Pestalotiopsis fici W106-1] |
| **144** | 573986939 | cutinase, putative [Cordyceps militaris CM01] |
| **144** | 667643269 | cutinase-2 protein [Beauveria bassiana ARSEF 2860] |
| **144** | 701778347 | putative cutinase 1 [Beauveria bassiana D1-5] |
| **145** | 477528349 | cutinase precursor [Colletotrichum orbiculare MAFF 240422] |
| **145** | 530479921 | cutinase [Colletotrichum gloeosporioides Cg-14] |
| **145** | 615444360 | cutinase [Colletotrichum fioriniae PJ7] |
| **145** | 697066507 | cutinase [Verticillium dahliae VdLs.17] |
| **145** | 759226692 | hypothetical protein PV09_04998 [Verruconis gallopava] |
| **146** | 748477957 | hypothetical protein AOL_s00004g25 [Arthrobotrys oligospora ATCC 24927] |
| **146** | 748499005 | hypothetical protein H072_4336 [Dactylellina haptotyla CBS 200.50] |
| **146** | 748512166 | hypothetical protein H072_7464 [Dactylellina haptotyla CBS 200.50] |
| **146** | 748513584 | hypothetical protein H072_7835 [Dactylellina haptotyla CBS 200.50] |
| **146** | 748525524 | hypothetical protein H072_11321 [Dactylellina haptotyla CBS 200.50] |
| **147** | 156036436 | hypothetical protein SS1G_12907 [Sclerotinia sclerotiorum 1980] |
| **147** | 29839380 | RecName: Full=Cutinase; AltName: Full=Cutin hydrolase; Flags: Precursor |
| **147** | 347826610 | carbohydrate esterase family 5 protein [Botrytis cinerea T4] |
| **147** | 521773519 | Cutinase [Blumeria graminis f. sp. tritici 96224] |
| **147** | 563298776 | hypothetical protein SBOR_0736 [Sclerotinia borealis F-4157] |
| **147** | 597574467 | putative Cutinase [Marssonina brunnea f. sp. 'multigermtubi' MB_m1] |
| **147** | 730175582 | putative cutinase [Erysiphe necator] |
| **147** | 730185322 | putative cutinase [Erysiphe necator] |
| **147** | 730186984 | putative cutinase [Erysiphe necator] |
| **148** | 302908268 | hypothetical protein NECHADRAFT_30450 [Nectria haematococca mpVI 77-13-4] |
| **148** | 342878797 | hypothetical protein FOXB_09361 [Fusarium oxysporum Fo5176] |
| **148** | 517310282 | uncharacterized protein FFUJ_01779 [Fusarium fujikuroi IMI 58289] |
| **148** | 584141097 | hypothetical protein FVEG_09653 [Fusarium verticillioides 7600] |
| **148** | 596542157 | hypothetical protein FG05_01570 [Fusarium graminearum] |
| **148** | 597576061 | cutinase [Marssonina brunnea f. sp. 'multigermtubi' MB_m1] |
| **148** | 636759935 | alpha/beta-Hydrolase [Glarea lozoyensis ATCC 20868] |
| **148** | 667828772 | hypothetical protein W97_02465 [Coniosporium apollinis CBS 100218] |
| **148** | 685850233 | hypothetical protein FPSE_00764 [Fusarium pseudograminearum CS3096] |
| **148** | 695123561 | unnamed protein product [Fusarium sp. FIESC_5 CS3069] |
| **148** | 751347977 | cutinase precursor [Fusarium avenaceum] |
| **148** | 758190008 | cutinase precursor [Fusarium graminearum PH-1] |
| **148** | 821078377 | putative cutinase [Diaporthe ampelina] |
| **149** | 302422734 | cutinase [Verticillium alfalfae VaMs.102] |
| **149** | 380487347 | cutinase [Colletotrichum higginsianum] |
| **149** | 530461018 | cutinase [Colletotrichum gloeosporioides Cg-14] |
| **149** | 596691934 | cutinase [Colletotrichum gloeosporioides Nara gc5] |
| **149** | 615462579 | cutinase [Colletotrichum fioriniae PJ7] |
| **149** | 697069959 | cutinase [Verticillium dahliae VdLs.17] |
| **149** | 751746517 | carbohydrate esterase family 5 protein [Oidiodendron maius Zn] |
| **150** | 225680449 | hypothetical protein PABG_01052 [Paracoccidioides brasiliensis Pb03] |
| **150** | 701690260 | hypothetical protein PAAG_12274 [Paracoccidioides sp. 'lutzii' Pb01] |
| **151** | 501363041 | cutinase [Mycobacterium marinum] |
| **151** | 556608188 | cutinase [Mycobacterium kansasii] |
| **151** | 738454196 | cutinase [Mycobacterium kansasii] |
| **151** | 738465572 | cutinase [Mycobacterium gastri] |
| **151** | 829092610 | cutinase [Mycobacterium haemophilum] |
| **152** | 857807599 | Cutinase [Mycobacterium sp. GPK 1020] |
| **152** | 858009905 | Cutinase [Mycobacterium sp. WCM 7299] |
| **153** | 748258742 | cutinase [Nocardia otitidiscaviarum] |
| **153** | 750534711 | cutinase [Nocardia concava] |
| **153** | 759916272 | cutinase [Nocardia otitidiscaviarum] |
| **154** | 398388860 | cutinase [Zymoseptoria tritici IPO323] |
| **154** | 398408083 | cutinase [Zymoseptoria tritici IPO323] |
| **154** | 453083289 | carbohydrate esterase family 5 protein [Sphaerulina musiva SO2202] |
| **154** | 796695272 | Chain A like protein [Zymoseptoria brevis] |
| **154** | 796707233 | cutinase like protein [Zymoseptoria brevis] |
| **155** | 549042908 | Similar to Cutinase; acc. no. Q8X1P1 [Pyronema omphalodes CBS 100304] |
| **155** | 549048373 | Similar to Cutinase; acc. no. Q8TGB8 [Pyronema omphalodes CBS 100304] |
| **155** | 549053180 | Similar to Cutinase; acc. no. Q9Y7G8 [Pyronema omphalodes CBS 100304] |
| **155** | 549056352 | Similar to Cutinase; acc. no. Q8X1P1 [Pyronema omphalodes CBS 100304] |
| **156** | 302422638 | conserved hypothetical protein [Verticillium alfalfae VaMs.102] |
| **156** | 380495941 | cutinase [Colletotrichum higginsianum] |
| **156** | 477529032 | cutinase [Colletotrichum orbiculare MAFF 240422] |
| **156** | 530467552 | cutinase [Colletotrichum gloeosporioides Cg-14] |
| **156** | 596709992 | cutinase [Colletotrichum gloeosporioides Nara gc5] |
| **156** | 615468998 | cutinase [Colletotrichum fioriniae PJ7] |
| **156** | 630014533 | hypothetical protein PFICI_04480 [Pestalotiopsis fici W106-1] |
| **156** | 640926883 | putative cutinase [Colletotrichum sublineola] |
| **156** | 697069867 | triacylglycerol lipase [Verticillium dahliae VdLs.17] |
| **156** | 821082146 | putative cutinase [Diaporthe ampelina] |
| **156** | 827071516 | cutinase [Colletotrichum graminicola M1.001] |
| **157** | 302503069 | cutinase, putative [Arthroderma benhamiae CBS 112371] |
| **157** | 302659792 | cutinase, putative [Trichophyton verrucosum HKI 0517] |
| **157** | 326474173 | cutinase [Trichophyton tonsurans CBS 112818] |
| **157** | 327296247 | cutinase [Trichophyton rubrum CBS 118892] |
| **158** | 261196822 | cutinase [Blastomyces gilchristii] |
| **158** | 821509721 | cutinase [Emmonsia crescens UAMH 3008] |
| **158** | 824366356 | cutinase [Emmonsia parva UAMH 139] |
| **159** | 154279758 | predicted protein [Histoplasma capsulatum NAm1] |
| **159** | 225562731 | cutinase [Histoplasma capsulatum G186AR] |
| **159** | 240279542 | cutinase [Histoplasma capsulatum H143] |
| **160** | 738519645 | cutinase [Mycobacterium genavense] |
| **160** | 738520924 | cutinase [Mycobacterium triplex] |
| **160** | 764935588 | cutinase [Mycobacterium simiae] |
| **161** | 302412090 | cutinase-2 [Verticillium alfalfae VaMs.102] |
| **161** | 380479355 | cutinase [Colletotrichum higginsianum] |
| **161** | 389641269 | cutinase 1 [Magnaporthe oryzae 70-15] |
| **161** | 477532600 | cutinase precursor [Colletotrichum orbiculare MAFF 240422] |
| **161** | 530469357 | cutinase [Colletotrichum gloeosporioides Cg-14] |
| **161** | 615461470 | cutinase [Colletotrichum fioriniae PJ7] |
| **161** | 640921206 | putative cutinase [Colletotrichum sublineola] |
| **161** | 666398936 | hypothetical protein S7711_08069 [Stachybotrys chartarum IBT 7711] |
| **161** | 827077303 | cutinase [Colletotrichum graminicola M1.001] |
| **162** | 169624696 | hypothetical protein SNOG_15608 [Phaeosphaeria nodorum SN15] |
| **162** | 189198862 | cutinase precursor [Pyrenophora tritici-repentis Pt-1C-BFP] |
| **162** | 330914278 | hypothetical protein PTT_06704 [Pyrenophora teres f. teres 0-1] |
| **162** | 396464483 | hypothetical protein LEMA_P043880.1 [Leptosphaeria maculans JN3] |
| **162** | 452003466 | carbohydrate esterase family 5 protein, partial [Bipolaris maydis C5] |
| **162** | 578493188 | carbohydrate esterase family 5 protein [Bipolaris victoriae FI3] |
| **162** | 627820357 | carbohydrate esterase family 5 protein [Bipolaris oryzae ATCC 44560] |
| **162** | 636580335 | carbohydrate esterase family 5 protein [Setosphaeria turcica Et28A] |
| **163** | 521774262 | cutinase precursor [Blumeria graminis f. sp. tritici 96224] |
| **163** | 528302866 | cutinase [Blumeria graminis f. sp. hordei DH14] |
| **163** | 730184063 | putative carbohydrate esterase family 5 protein [Erysiphe necator] |
| **164** | 154302189 | hypothetical protein BC1G_09775 [Botrytis cinerea B05.10] |
| **164** | 472246483 | putative cutinase precursor protein [Botrytis cinerea BcDW1] |
| **164** | 597573159 | cutinase [Marssonina brunnea f. sp. 'multigermtubi' MB_m1] |
| **165** | 169616334 | hypothetical protein SNOG_11338 [Phaeosphaeria nodorum SN15] |
| **165** | 189197533 | cutinase precursor [Pyrenophora tritici-repentis Pt-1C-BFP] |
| **165** | 330935699 | hypothetical protein PTT_17835 [Pyrenophora teres f. teres 0-1] |
| **165** | 396480690 | hypothetical protein LEMA_P089840.1 [Leptosphaeria maculans JN3] |
| **165** | 396484681 | hypothetical protein LEMA_P077490.1 [Leptosphaeria maculans JN3] |
| **165** | 451993053 | carbohydrate esterase family 5 protein, partial [Bipolaris maydis C5] |
| **165** | 578484651 | carbohydrate esterase family 5 protein [Bipolaris victoriae FI3] |
| **165** | 627833849 | carbohydrate esterase family 5 protein [Bipolaris oryzae ATCC 44560] |
| **165** | 636578107 | carbohydrate esterase family 5 protein [Setosphaeria turcica Et28A] |
| **166** | 389628920 | hypothetical protein MGG_05798 [Magnaporthe oryzae 70-15] |
| **166** | 685419909 | hypothetical protein GGTG_12644 [Gaeumannomyces graminis var. tritici R3-111a-1] |
| **166** | 835898762 | hypothetical protein MAPG_09146 [Magnaporthiopsis poae ATCC 64411] |
| **167** | 389623035 | cutinase [Magnaporthe oryzae 70-15] |
| **167** | 631378438 | carbohydrate esterase family 5 protein [Pseudocercospora fijiensis CIRAD86] |
| **167** | 835889458 | cutinase [Magnaporthiopsis poae ATCC 64411] |
| **168** | 342888911 | hypothetical protein FOXB_01352 [Fusarium oxysporum Fo5176] |
| **168** | 440463620 | cutinase [Magnaporthe oryzae Y34] |
| **168** | 440476190 | cutinase precursor [Magnaporthe oryzae Y34] |
| **168** | 587680539 | hypothetical protein FOYG_02076 [Fusarium oxysporum FOSC 3-a] |
| **168** | 587722682 | hypothetical protein FOWG_06652 [Fusarium oxysporum f. sp. lycopersici MN25] |
| **168** | 590024460 | hypothetical protein FOMG_17063 [Fusarium oxysporum f. sp. melonis 26406] |
| **168** | 630015057 | hypothetical protein PFICI_04742 [Pestalotiopsis fici W106-1] |
| **169** | 154310781 | hypothetical protein BC1G_06369 [Botrytis cinerea B05.10] |
| **169** | 156033332 | hypothetical protein SS1G_13386 [Sclerotinia sclerotiorum 1980] |
| **169** | 563292923 | hypothetical protein SBOR_6308 [Sclerotinia borealis F-4157] |
| **170** | 295899630 | Cutinase [Mycobacterium parascrofulaceum ATCC BAA-614] |
| **170** | 386789345 | serine esterase, cutinase family protein [Mycobacterium sp. MOTT36Y] |
| **170** | 504193039 | MULTISPECIES: cutinase [Mycobacterium avium complex (MAC)] |
| **170** | 564152739 | cutinase [Mycobacterium avium 10-5581] |
| **170** | 738507113 | MULTISPECIES: cutinase [Mycobacterium] |
| **170** | 750348407 | cutinase [Mycobacterium colombiense] |
| **170** | 757674744 | cutinase [Mycobacterium avium] |
| **170** | 806834142 | cutinase [Mycobacterium nebraskense] |
| **170** | 808658883 | serine esterase, cutinase [Mycobacterium europaeum] |
| **171** | 656905783 | hypothetical protein A1O9_12447 [Exophiala aquamarina CBS 119918] |
| **171** | 671379443 | hypothetical protein HMPREF1541_07065 [Cyphellophora europaea CBS 101466] |
| **172** | 477536600 | cutinase precursor [Colletotrichum orbiculare MAFF 240422] |
| **172** | 530465802 | cutinase [Colletotrichum gloeosporioides Cg-14] |
| **172** | 615472316 | hypothetical protein CFIO01_04189 [Colletotrichum fioriniae PJ7] |
| **172** | 685850091 | hypothetical protein FPSE_00693 [Fusarium pseudograminearum CS3096] |
| **172** | 758194528 | hypothetical protein FGSG_10634 [Fusarium graminearum PH-1] |
| **173** | 119480811 | cutinase, putative [Neosartorya fischeri NRRL 181] |
| **173** | 121698952 | cutinase, putative [Aspergillus clavatus NRRL 1] |
| **173** | 71001184 | cutinase [Aspergillus fumigatus Af293] |
| **173** | 849270814 | probable cutinase 1 [Neosartorya udagawae] |
| **174** | 380490868 | cutinase [Colletotrichum higginsianum] |
| **174** | 530460925 | cutinase [Colletotrichum gloeosporioides Cg-14] |
| **174** | 596686571 | cutinase precursor [Colletotrichum gloeosporioides Nara gc5] |
| **174** | 615441951 | cutinase [Colletotrichum fioriniae PJ7] |
| **174** | 640918847 | putative cutinase [Colletotrichum sublineola] |
| **174** | 821085594 | putative cutinase precursor [Diaporthe ampelina] |
| **174** | 827058753 | cutinase [Colletotrichum graminicola M1.001] |
| **175** | 407927884 | Cutinase [Macrophomina phaseolina MS6] |
| **175** | 615399858 | putative cutinase protein [Neofusicoccum parvum UCRNP2] |
| **175** | 685408777 | hypothetical protein GGTG_07159 [Gaeumannomyces graminis var. tritici R3-111a-1] |
| **175** | 685415698 | hypothetical protein GGTG_10566 [Gaeumannomyces graminis var. tritici R3-111a-1] |
| **175** | 821061751 | putative cutinase precursor [Phaeomoniella chlamydospora] |
| **175** | 835894375 | hypothetical protein MAPG_05317 [Magnaporthiopsis poae ATCC 64411] |
| **176** | 597578123 | cutinase precursor [Marssonina brunnea f. sp. 'multigermtubi' MB_m1] |
| **176** | 597585327 | cutinase [Marssonina brunnea f. sp. 'multigermtubi' MB_m1] |
| **176** | 636766473 | alpha/beta-Hydrolase [Glarea lozoyensis ATCC 20868] |
| **177** | 451998146 | carbohydrate esterase family 5 protein [Bipolaris maydis C5] |
| **177** | 627819255 | carbohydrate esterase family 5 protein [Bipolaris oryzae ATCC 44560] |
| **177** | 628087038 | carbohydrate esterase family 5 protein [Bipolaris sorokiniana ND90Pr] |
| **177** | 628207595 | carbohydrate esterase family 5 protein [Bipolaris zeicola 26-R-13] |
| **178** | 119499750 | cutinase family protein [Neosartorya fischeri NRRL 181] |
| **178** | 145242926 | cutinase 2 [Aspergillus niger CBS 513.88] |
| **178** | 169765364 | cutinase 1 [Aspergillus oryzae RIB40] |
| **178** | 238493425 | cutinase, putative [Aspergillus flavus NRRL3357] |
| **178** | 255956699 | Pc21g21220 [Penicillium rubens Wisconsin 54-1255] |
| **178** | 358367545 | cutinase [Aspergillus kawachii IFO 4308] |
| **178** | 525586265 | putative cutinase [Penicillium oxalicum 114-2] |
| **178** | 666436342 | cutinase [Aspergillus fumigatus var. RP-2014] |
| **178** | 768695920 | Cutinase monofunctional [Penicillium solitum] |
| **178** | 849274517 | probable cutinase 2 [Neosartorya udagawae] |
| **178** | 859263852 | Putative Cutinase [Penicillium brasilianum] |
| **178** | 94709368 | RecName: Full=Probable cutinase 2; AltName: Full=Cutin hydrolase 2; Flags: Precursor |
| **179** | 302418434 | cutinase [Verticillium alfalfae VaMs.102] |
| **179** | 342878365 | hypothetical protein FOXB_09757 [Fusarium oxysporum Fo5176] |
| **179** | 475671636 | Cutinase [Fusarium oxysporum f. sp. cubense race 4] |
| **179** | 584142546 | hypothetical protein FVEG_10728 [Fusarium verticillioides 7600] |
| **179** | 587659181 | hypothetical protein FOYG_15790 [Fusarium oxysporum FOSC 3-a] |
| **179** | 587687012 | hypothetical protein FOZG_13329 [Fusarium oxysporum Fo47] |
| **179** | 587712004 | hypothetical protein FOWG_13254 [Fusarium oxysporum f. sp. lycopersici MN25] |
| **179** | 587737942 | hypothetical protein FOVG_13704 [Fusarium oxysporum f. sp. pisi HDV247] |
| **179** | 590026902 | hypothetical protein FOMG_14660 [Fusarium oxysporum f. sp. melonis 26406] |
| **179** | 697087014 | cutinase [Verticillium dahliae VdLs.17] |
| **179** | 699032740 | unnamed protein product [Fusarium graminearum] |
| **179** | 751355946 | hypothetical protein FAVG1_02228 [Fusarium avenaceum] |
| **180** | 302411364 | cutinase [Verticillium alfalfae VaMs.102] |
| **180** | 697084632 | cutinase [Verticillium dahliae VdLs.17] |
| **181** | 154317906 | hypothetical protein BC1G_02936 [Botrytis cinerea B05.10] |
| **181** | 156050149 | hypothetical protein SS1G_07661 [Sclerotinia sclerotiorum 1980] |
| **181** | 29839372 | RecName: Full=Cutinase; AltName: Full=Cutin hydrolase; Flags: Precursor |
| **181** | 563290788 | hypothetical protein SBOR_8242 [Sclerotinia borealis F-4157] |
| **182** | 154314211 | hypothetical protein BC1G_05199 [Botrytis cinerea B05.10] |
| **182** | 156036046 | hypothetical protein SS1G_12709 [Sclerotinia sclerotiorum 1980] |
| **182** | 169601064 | hypothetical protein SNOG_03386 [Phaeosphaeria nodorum SN15] |
| **182** | 189193483 | cutinase precursor [Pyrenophora tritici-repentis Pt-1C-BFP] |
| **182** | 330925614 | hypothetical protein PTT_12551 [Pyrenophora teres f. teres 0-1] |
| **182** | 380483212 | cutinase [Colletotrichum higginsianum] |
| **182** | 451998711 | carbohydrate esterase family 5 protein [Bipolaris maydis C5] |
| **182** | 477535535 | cutinase [Colletotrichum orbiculare MAFF 240422] |
| **182** | 530473181 | cutinase [Colletotrichum gloeosporioides Cg-14] |
| **182** | 563298727 | cutinase precursor [Sclerotinia borealis F-4157] |
| **182** | 597570237 | cutinase [Marssonina brunnea f. sp. 'multigermtubi' MB_m1] |
| **182** | 615469256 | cutinase [Colletotrichum fioriniae PJ7] |
| **182** | 627915113 | carbohydrate esterase family 5 protein [Bipolaris oryzae ATCC 44560] |
| **182** | 628080620 | carbohydrate esterase family 5 protein [Bipolaris sorokiniana ND90Pr] |
| **182** | 628184134 | carbohydrate esterase family 5 protein [Bipolaris zeicola 26-R-13] |
| **182** | 630026666 | Cutinase [Pestalotiopsis fici W106-1] |
| **182** | 636599729 | carbohydrate esterase family 5 protein [Setosphaeria turcica Et28A] |
| **182** | 636755383 | alpha/beta-Hydrolase [Glarea lozoyensis ATCC 20868] |
| **182** | 640928224 | putative cutinase [Colletotrichum sublineola] |
| **182** | 682283878 | hypothetical protein O988_00824 [Pseudogymnoascus pannorum VKM F-3808] |
| **182** | 682291924 | hypothetical protein V492_06679 [Pseudogymnoascus pannorum VKM F-4246] |
| **182** | 682328069 | hypothetical protein V491_00503 [Pseudogymnoascus pannorum VKM F-3775] |
| **182** | 682332863 | hypothetical protein V493_01309 [Pseudogymnoascus pannorum VKM F-4281 (FW-2241)] |
| **182** | 682358387 | hypothetical protein V496_10666 [Pseudogymnoascus pannorum VKM F-4515 (FW-2607)] |
| **182** | 682397334 | hypothetical protein V499_04862 [Pseudogymnoascus pannorum VKM F-103] |
| **182** | 682419843 | hypothetical protein V500_05627 [Pseudogymnoascus pannorum VKM F-4518 (FW-2643)] |
| **182** | 682451410 | hypothetical protein V501_05047 [Pseudogymnoascus pannorum VKM F-4519 (FW-2642)] |
| **182** | 827074776 | cutinase [Colletotrichum graminicola M1.001] |
| **183** | 336263790 | hypothetical protein SMAC_04107 [Sordaria macrospora k-hell] |
| **183** | 666867077 | hypothetical protein SAPIO_CDS4432 [Scedosporium apiospermum] |
| **184** | 748488617 | hypothetical protein AOL_s00043g155 [Arthrobotrys oligospora ATCC 24927] |
| **184** | 748519148 | hypothetical protein H072_9426 [Dactylellina haptotyla CBS 200.50] |
| **185** | 115387611 | cutinase precursor [Aspergillus terreus NIH2624] |
| **185** | 115390627 | cutinase precursor [Aspergillus terreus NIH2624] |
| **185** | 630026713 | hypothetical protein PFICI_09125 [Pestalotiopsis fici W106-1] |
| **185** | 816331239 | hypothetical protein AOCH_004426 [Aspergillus ochraceoroseus] |
| **185** | 821077031 | putative cutinase precursor [Diaporthe ampelina] |
| **186** | 302408527 | cutinase-2 [Verticillium alfalfae VaMs.102] |
| **186** | 399168757 | probable cutinase 1 precursor [Claviceps purpurea 20.1] |
| **186** | 632911811 | carbohydrate esterase family 5 [Ustilaginoidea virens] |
| **186** | 697080594 | cutinase [Verticillium dahliae VdLs.17] |
| **187** | 1169141 | Alternaria brassicicola |
| **187** | 189210427 | cutinase precursor [Pyrenophora tritici-repentis Pt-1C-BFP] |
| **187** | 330929009 | hypothetical protein PTT_14312 [Pyrenophora teres f. teres 0-1] |
| **188** | 115401686 | cutinase precursor [Aspergillus terreus NIH2624] |
| **188** | 255935839 | Pc13g05110 [Penicillium rubens Wisconsin 54-1255] |
| **189** | 628852669 | carbohydrate esterase family 5 protein [Coniophora puteana RWD-64-598 SS2] |
| **189** | 761945566 | carbohydrate esterase family 5 protein [Cylindrobasidium torrendii FP15055 ss-10] |
| **189** | 816188296 | hypothetical protein PISL3812_06279 [Talaromyces islandicus] |
| **190** | 751745794 | carbohydrate esterase family 5 protein [Oidiodendron maius Zn] |
| **190** | 751748349 | carbohydrate esterase family 5 protein [Oidiodendron maius Zn] |
| **191** | 115396660 | cutinase precursor [Aspergillus terreus NIH2624] |
| **191** | 119487393 | cutinase, putative [Neosartorya fischeri NRRL 181] |
| **191** | 121714599 | cutinase, putative [Aspergillus clavatus NRRL 1] |
| **191** | 666431560 | cutinase [Aspergillus fumigatus var. RP-2014] |
| **191** | 849267222 | probable cutinase 3 [Neosartorya udagawae] |
| **192** | 169782674 | cutinase 1 [Aspergillus oryzae RIB40] |
| **192** | 350639345 | hypothetical protein ASPNIDRAFT_41640 [Aspergillus niger ATCC 1015] |
| **192** | 358370465 | cutinase [Aspergillus kawachii IFO 4308] |
| **192** | 599160332 | cutinase 1 [Aspergillus ruber CBS 135680] |
| **192** | 67542033 | hypothetical protein AN7180.2 [Aspergillus nidulans FGSC A4] |
| **192** | 770302748 | Cutinase [Aspergillus parasiticus SU-1] |
| **192** | 859263669 | hypothetical protein PMG11_07080 [Penicillium brasilianum] |
| **193** | 596551579 | hypothetical protein FG05_02342 [Fusarium graminearum] |
| **193** | 685856287 | hypothetical protein FPSE_03781 [Fusarium pseudograminearum CS3096] |
| **193** | 699043632 | unnamed protein product [Fusarium graminearum] |
| **193** | 758191744 | cutinase precursor [Fusarium graminearum PH-1] |
| **194** | 116198469 | hypothetical protein CHGG_07390 [Chaetomium globosum CBS 148.51] |
| **194** | 367051276 | carbohydrate esterase family 5 protein [Thielavia terrestris NRRL 8126] |
| **194** | 672797746 | Cutinase-like protein [Acremonium chrysogenum ATCC 11550] |
| **195** | 358382814 | carbohydrate esterase family 5 protein [Trichoderma virens Gv29-8] |
| **195** | 818161616 | cutinase [Trichoderma harzianum] |
| **196** | 117650 | RecName: Full=Cutinase; AltName: Full=Cutin hydrolase; Flags: Precursor |
| **196** | 117651 | RecName: Full=Cutinase 1; AltName: Full=Cutin hydrolase 1; Flags: Precursor |
| **196** | 213424159 | Chain A, Glomerella Cingulata Apo Cutinase |
| **196** | 477524946 | cutinase precursor [Colletotrichum orbiculare MAFF 240422] |
| **196** | 615475570 | cutinase [Colletotrichum fioriniae PJ7] |
| **196** | 822591845 | cutinase [Colletotrichum gloeosporioides] |
| **197** | 302405709 | cutinase [Verticillium alfalfae VaMs.102] |
| **197** | 342877647 | hypothetical protein FOXB_10382 [Fusarium oxysporum Fo5176] |
| **197** | 475670288 | Cutinase 1 [Fusarium oxysporum f. sp. cubense race 4] |
| **197** | 584144763 | cutinase [Fusarium verticillioides 7600] |
| **197** | 587659826 | cutinase [Fusarium oxysporum FOSC 3-a] |
| **197** | 672792829 | Cutinase-like protein [Acremonium chrysogenum ATCC 11550] |
| **197** | 697082544 | cutinase [Verticillium dahliae VdLs.17] |
| **197** | 829105592 | Uncharacterized protein LW93_9455 [Fusarium fujikuroi] |
| **198** | 169772161 | cutinase precursor [Aspergillus oryzae RIB40] |
| **198** | 770308344 | Cutinase [Aspergillus parasiticus SU-1] |
| **199** | 525582098 | putative cutinase [Penicillium oxalicum 114-2] |
| **199** | 859270068 | hypothetical protein PMG11_00008 [Penicillium brasilianum] |
| **200** | 212535488 | cutinase, putative [Talaromyces marneffei ATCC 18224] |
| **200** | 242793180 | cutinase, putative [Talaromyces stipitatus ATCC 10500] |
| **200** | 748557621 | cutinase [Talaromyces cellulolyticus] |
| **200** | 816190744 | Cutinase [Talaromyces islandicus] |
| **201** | 682301407 | hypothetical protein V492_03390 [Pseudogymnoascus pannorum VKM F-4246] |
| **201** | 682333637 | hypothetical protein V493_01007 [Pseudogymnoascus pannorum VKM F-4281 (FW-2241)] |
| **201** | 682379227 | hypothetical protein V496_04350 [Pseudogymnoascus pannorum VKM F-4515 (FW-2607)] |
| **201** | 682395177 | hypothetical protein V499_06075 [Pseudogymnoascus pannorum VKM F-103] |
| **201** | 682429476 | hypothetical protein V498_03087 [Pseudogymnoascus pannorum VKM F-4517 (FW-2822)] |
| **202** | 682264873 | hypothetical protein V490_06128 [Pseudogymnoascus pannorum VKM F-3557] |
| **202** | 682270825 | hypothetical protein O988_06104 [Pseudogymnoascus pannorum VKM F-3808] |
| **202** | 682295954 | hypothetical protein V492_05213 [Pseudogymnoascus pannorum VKM F-4246] |
| **202** | 682347576 | hypothetical protein V495_05499 [Pseudogymnoascus pannorum VKM F-4514 (FW-929)] |
| **202** | 682352941 | hypothetical protein V494_01804 [Pseudogymnoascus pannorum VKM F-4513 (FW-928)] |
| **202** | 682412587 | hypothetical protein V500_09223 [Pseudogymnoascus pannorum VKM F-4518 (FW-2643)] |
| **202** | 682438926 | hypothetical protein V502_11496 [Pseudogymnoascus pannorum VKM F-4520 (FW-2644)] |
| **203** | 1045205 | cut1 [Magnaporthe grisea] |
| **203** | 401888647 | cutinase [Trichosporon asahii var. asahii CBS 2479] |
| **204** | 116203001 | hypothetical protein CHGG_09385 [Chaetomium globosum CBS 148.51] |
| **204** | 367029345 | carbohydrate esterase family 5 protein [Myceliophthora thermophila ATCC 42464] |
| **204** | 576045666 | cutinase-like protein [Chaetomium thermophilum var. thermophilum DSM 1495] |
| **205** | 169625712 | hypothetical protein SNOG_16132 [Phaeosphaeria nodorum SN15] |
| **205** | 512381000 | cutinase [fungal sp. B47-9] |
| **205** | 666409960 | hypothetical protein S7711_05022 [Stachybotrys chartarum IBT 7711] |
| **205** | 667728452 | hypothetical protein S40285_04613 [Stachybotrys chlorohalonata IBT 40285] |
| **205** | 667740225 | hypothetical protein S40288_02359 [Stachybotrys chartarum IBT 40288] |
| **206** | 171684047 | hypothetical protein [Podospora anserina S mat+] |
| **206** | 171694704 | hypothetical protein [Podospora anserina S mat+] |
| **206** | 347976421 | unnamed protein product [Podospora anserina S mat+] |
| **207** | 189210263 | cutinase precursor [Pyrenophora tritici-repentis Pt-1C-BFP] |
| **207** | 330921100 | hypothetical protein PTT_10243 [Pyrenophora teres f. teres 0-1] |
| **208** | 451995649 | carbohydrate esterase family 5 protein [Bipolaris maydis C5] |
| **208** | 578492124 | carbohydrate esterase family 5 protein [Bipolaris victoriae FI3] |
| **208** | 627824766 | carbohydrate esterase family 5 protein [Bipolaris oryzae ATCC 44560] |
| **208** | 628069111 | carbohydrate esterase family 5 protein, partial [Bipolaris sorokiniana ND90Pr] |
| **209** | 302882620 | hypothetical protein NECHADRAFT_55040 [Nectria haematococca mpVI 77-13-4] |
| **209** | 751346299 | hypothetical protein FAVG1_12716 [Fusarium avenaceum] |
| **210** | 475672137 | Cutinase [Fusarium oxysporum f. sp. cubense race 4] |
| **210** | 477515815 | Cutinase [Fusarium oxysporum f. sp. cubense race 1] |
| **210** | 517316729 | probable cutinase precursor [Fusarium fujikuroi IMI 58289] |
| **210** | 587667491 | cutinase [Fusarium oxysporum FOSC 3-a] |
| **210** | 587708741 | cutinase [Fusarium oxysporum f. sp. lycopersici MN25] |
| **210** | 685862071 | hypothetical protein FPSE_06675 [Fusarium pseudograminearum CS3096] |
| **210** | 751348433 | cutinase [Fusarium avenaceum] |
| **210** | 758201106 | cutinase-1 precursor [Fusarium graminearum PH-1] |
| **211** | 148954201 | cutinase [Fusarium oxysporum] |
| **211** | 342889518 | hypothetical protein FOXB_00914 [Fusarium oxysporum Fo5176] |
| **211** | 517324211 | probable cutinase 1 precursor [Fusarium fujikuroi IMI 58289] |
| **211** | 587733182 | cutinase 3 [Fusarium oxysporum f. sp. pisi HDV247] |
| **211** | 685869273 | hypothetical protein FPSE_10277 [Fusarium pseudograminearum CS3096] |
| **211** | 751349976 | cutinase 3 [Fusarium avenaceum] |
| **211** | 168146 | Fusarium solani |

^†^ Phylogenetic clades are shown in Figure 1
