# Supplementary figures and images for: Combined use of cutinase and high-resolution mass-spectrometry to query the molecular architecture of cutin
Source: Plant Methods. 2018 Dec 26;14:117. doi: 10.1186/s13007-018-0384-6 (PMC6306009; doi:10.1186/s13007-018-0384-6)

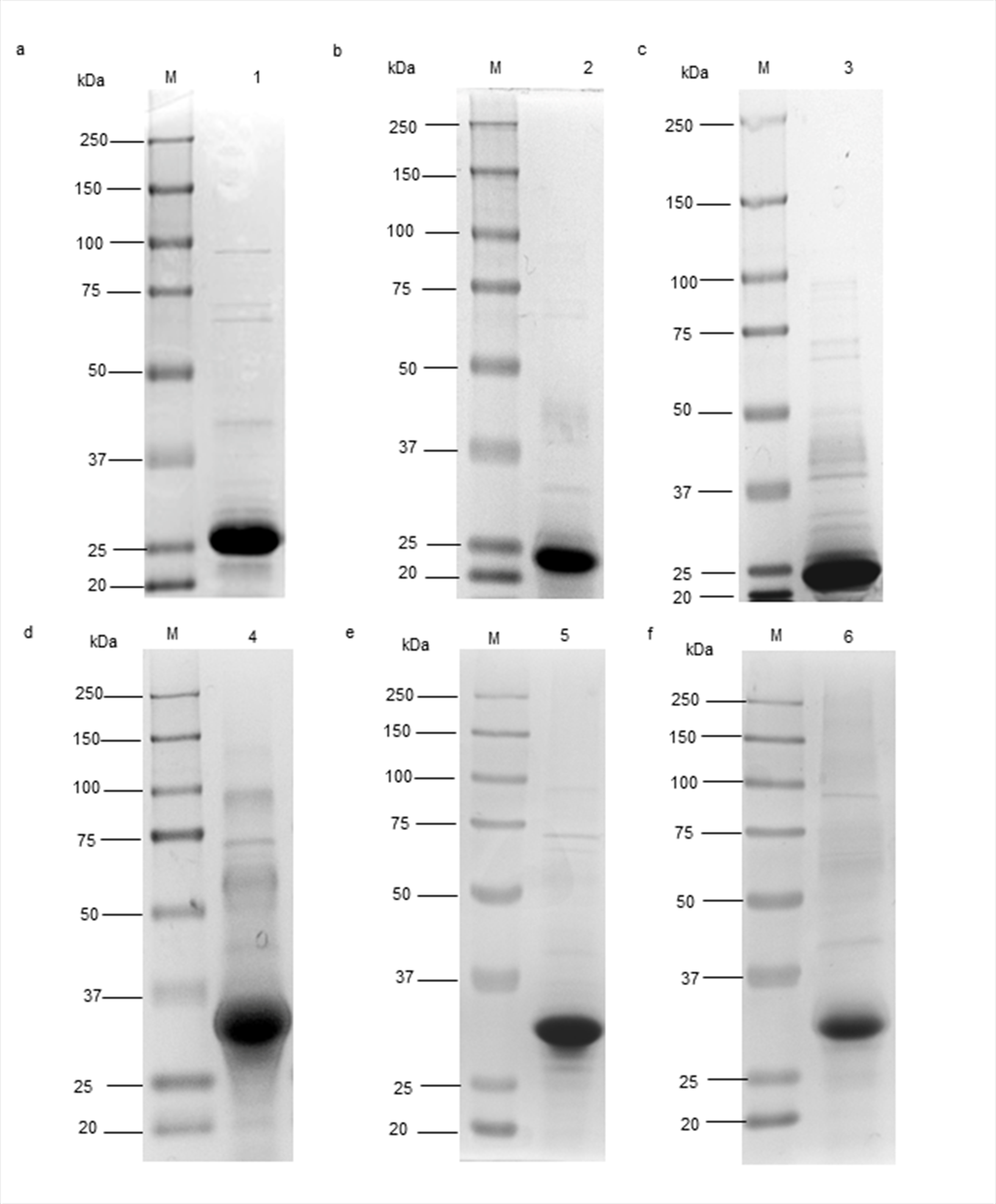

Supplement: Supplementary file 2 — Additional file 2: Fig. S1. SDS-PAGE analysis of purified cutinases recombinantly expressed in E. coli. a) Fusarium solani cutinase (gi 168146); b) Alternaria brassicicola cutinase (gi 1169141); c) Blastomyces gilchristii cutinase (gi 261196822); d) Bipolaris victoriae cutinase (gi 578495481); e) Catenuloplanes japonicus cutinase (gi 703060160); f) Oidiodendron maius cutinase (gi 751745794). [file 13007_2018_384_MOESM2_ESM.tif]

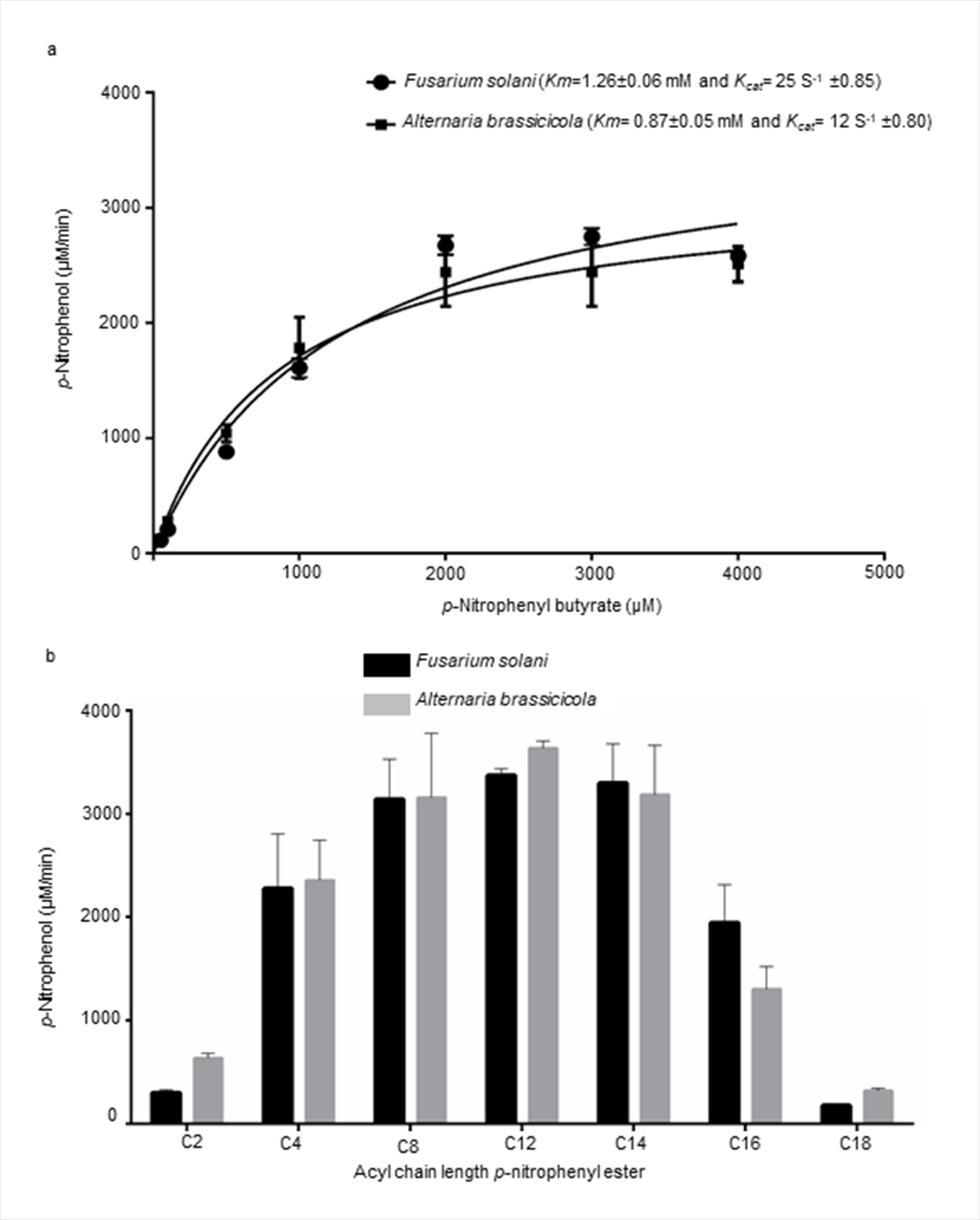

Supplement: Supplementary file 3 — Additional file 3: Fig. S2. Enzymological characterization of F. solani and A. brassicicola cutinases. Cutinase activity was determined as the rate of p-nitrophenyl butyrate ester hydrolysis, which was monitored by the increasing absorbance at 405 nm. a) The dependence of F. solani and A. brassicicola cutinase on the concentration of the substrate p-nitrophenyl butyrate ester. Error bars represent the standard deviation of three determinations. b) Substrate specificity of F. solani and A. brassicicola cutinases using the substrates p-nitrophenyl acetate (C2), p-nitrophenyl butyrate (C4), p-nitrophynyl octanoate (C8), p-nitrophynyl laurate (C12), p-nitrophenyl myristate (C14), p-nitrophenyl palmitate (C16) and p-nitrophenyl stearate (C18). Error bars correspond to the standard deviation of three determinations. [file 13007_2018_384_MOESM3_ESM.tif]

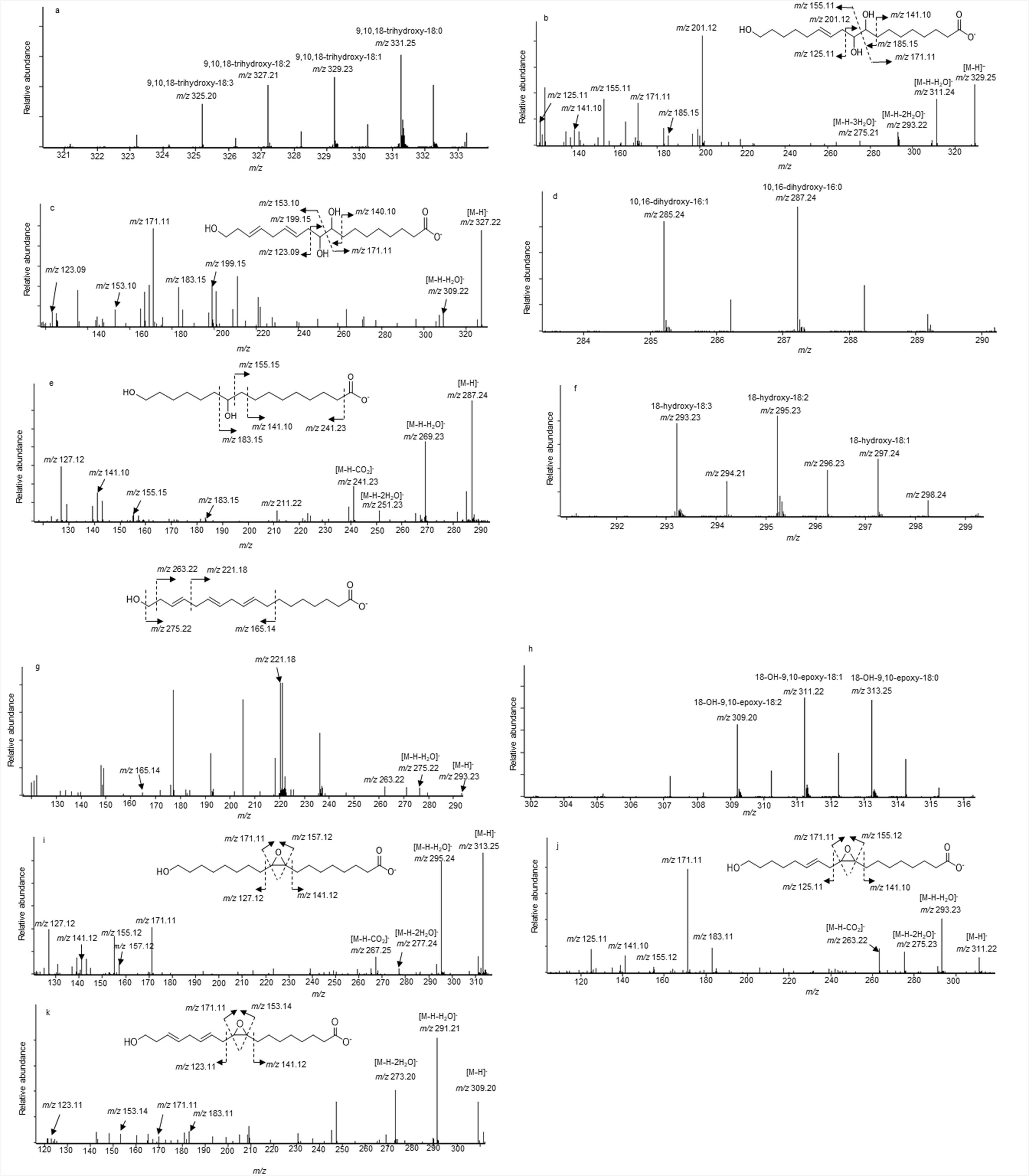

Supplement: Supplementary file 4 — Additional file 4: Fig. S3. LC–MS and LC–MS/MS characterization of individual cutin monomers of saturated and unsaturated FAs a) trihydroxy-18-carbon FAs; b) 9,10,18-trihydroxy-18:1 (m/z 329.25); c) 9,10,18-trihydroxy-18:2 (m/z 327.22); d) dihydroxy-16-carbon FAs; e) 10,16-dihydroxy-16:0 (m/z 287.24); f) hydroxy-18-carbon FAs; g) 18-hydroxy-18:3 (m/z 293.23); h) hydroxy-9,10-epoxy-18-carbon FAs; i) 18-hydroxy-9,10-epoxy-18:0 (m/z 313.25); j) 18-hydroxy-9,10-epoxy-18:1 (m/z 311.22); k) 18-hydroxy-9,10-epoxy-18:2 (m/z 309.20). [file 13007_2018_384_MOESM4_ESM.tif]

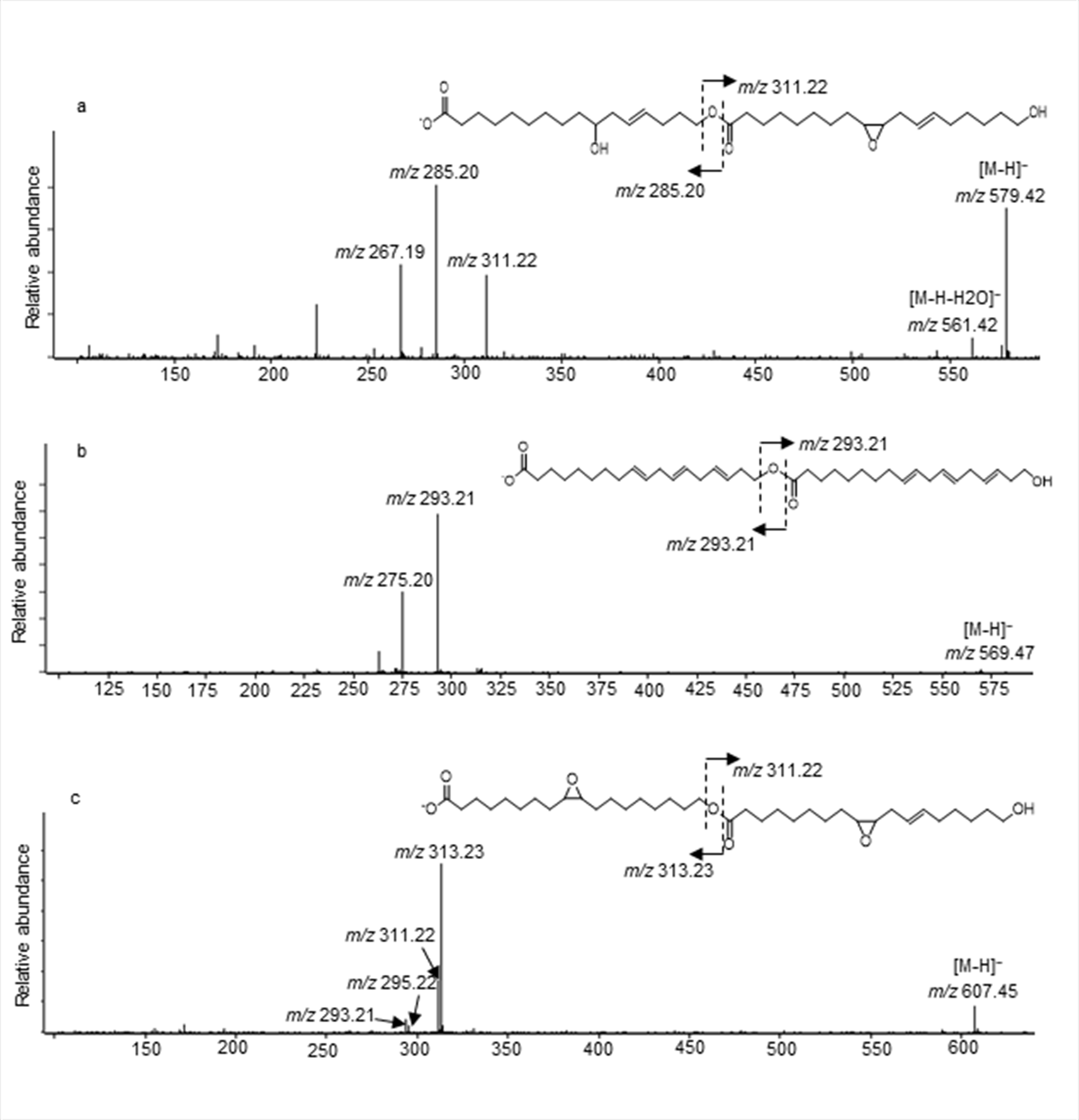

Supplement: Supplementary file 5 — Additional file 5: Fig. S4. LC–MS/MS fragmentation spectra of cutinase-generated cutin dimers: a) 10,16-dihydroxy-16:1 acylated by 18-hydroxy-9,10-epoxy-18:1 (m/z 579.42); b) 18-hydroxy-18:3 acylated by 18-hydroxy-18:3 (m/z 569.47); c) 18-hydroxy-9,10-epoxy-18:0 acylated by 18-hydroxy-9,10-epoxy-18:1 (m/z 607.45). [file 13007_2018_384_MOESM5_ESM.tif]

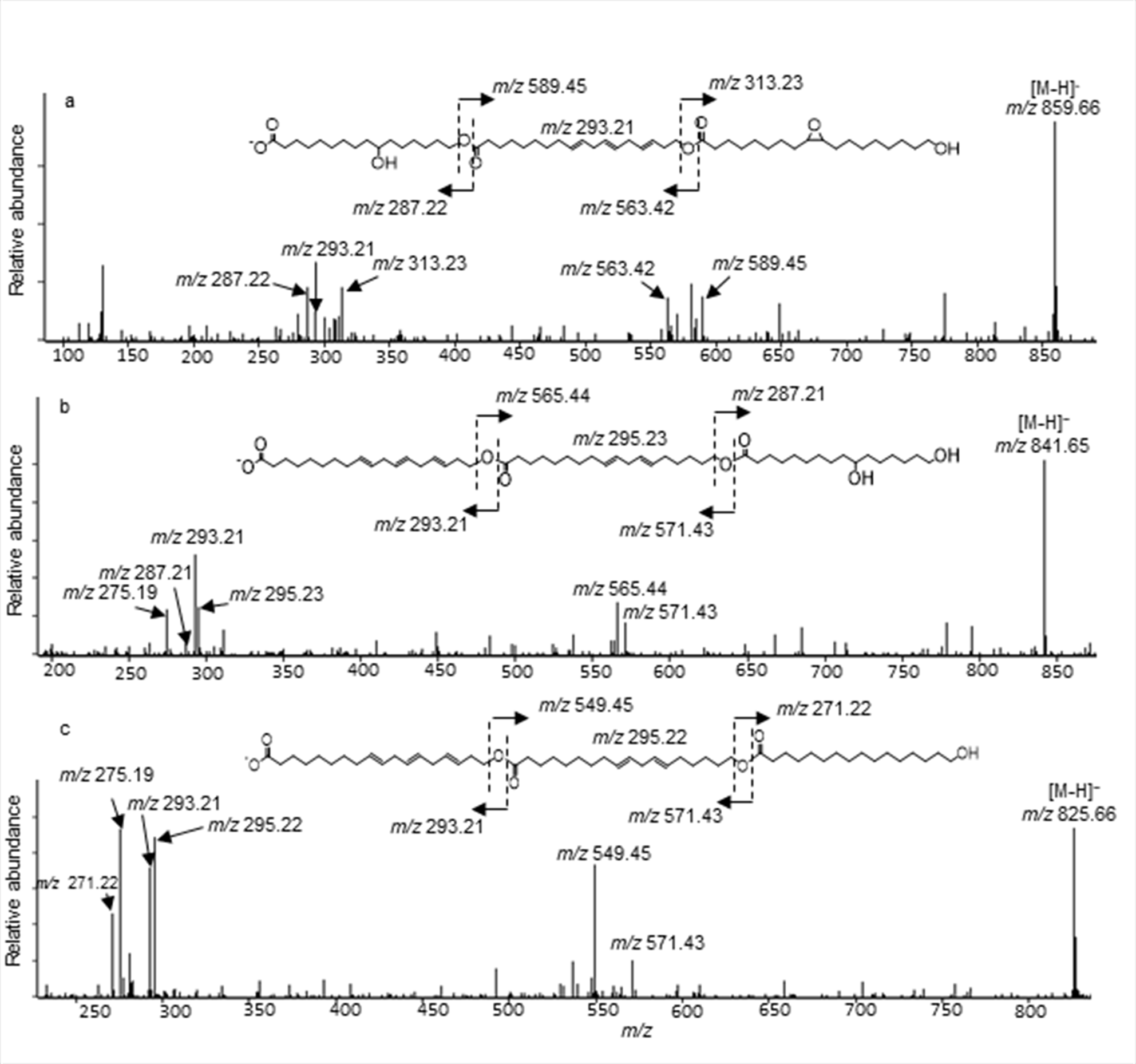

Supplement: Supplementary file 6 — Additional file 6: Fig. S5. LC–MS/MS fragmentation spectra of cutinase-generated cutin trimers: a) 10,16-dihydroxy-16:0 acylated by 18-hydroxy-18:3, which is acylated by 18-hydroxy-9,10-epoxy-18:0 (m/z 859.66); b) 18-hydroxy-18:3 acylated by 18-hydroxy-18:2, which is acylated by 10,16-dihydroxy-16:0 (m/z 841.65); c) 18-hydroxy-18:3 acylated by 18-hydroxy-18:2, which is acylated by 16-hydroxy-16:0 (m/z 825.66). [file 13007_2018_384_MOESM6_ESM.tif]

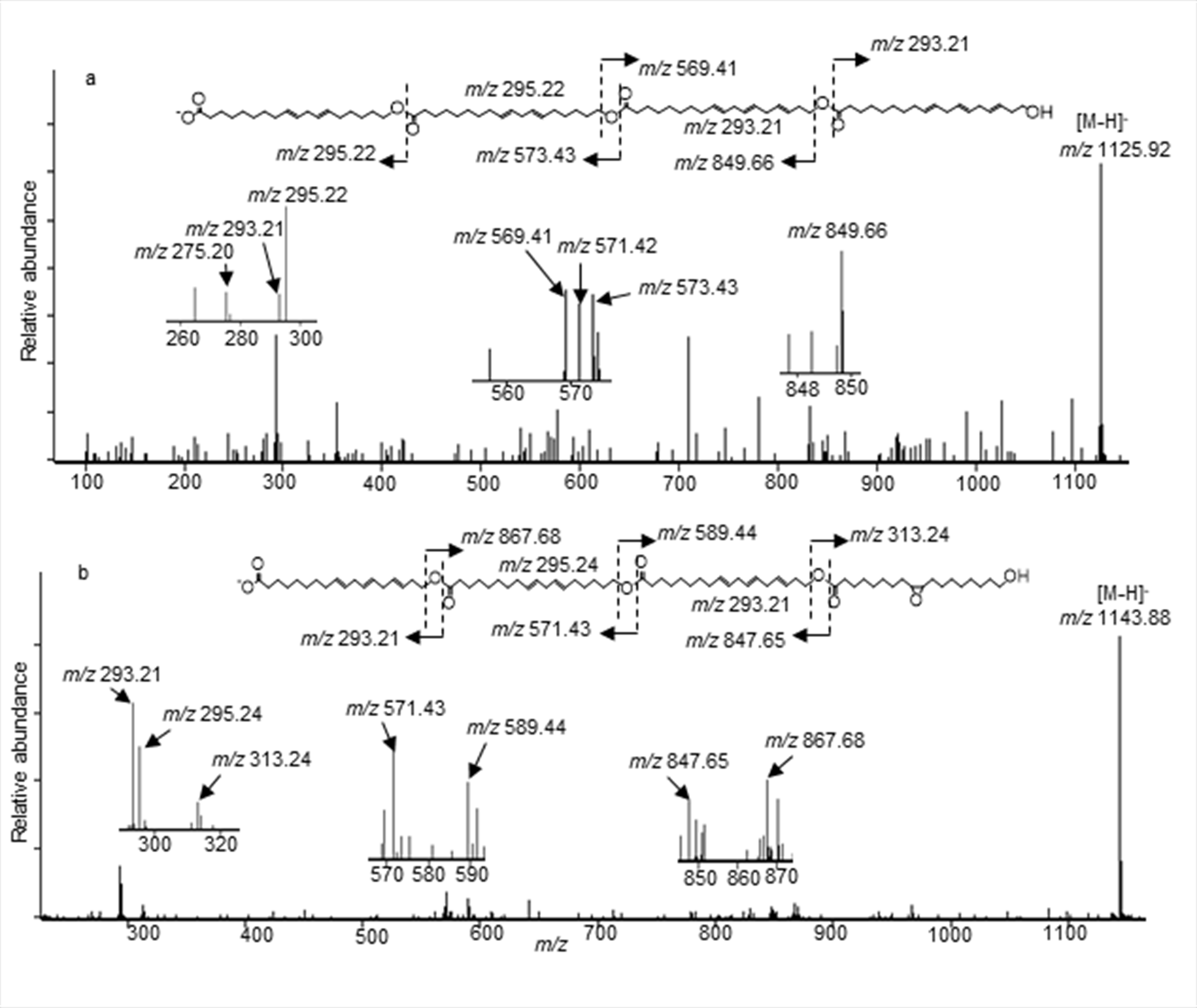

Supplement: Supplementary file 7 — Additional file 7: Fig. S6. LC–MS/MS fragmentation spectra of cutinase-generated cutin tetramers: a) 18-hydroxy-18:2 acylated by 18-hydroxy-18:2, which is acylated by 18-hydroxy-18:3, which is further acylated by 18-hydroxy-18:3 (m/z 1125.92); b) 18-hydroxy-18:3 acylated by 18-hydroxy-18:2, which is acylated by 18-hydroxy-18:3, which is further acylated by 18-hydroxy-9,10-epoxy-18:0 (m/z 1143.88). [file 13007_2018_384_MOESM7_ESM.tif]

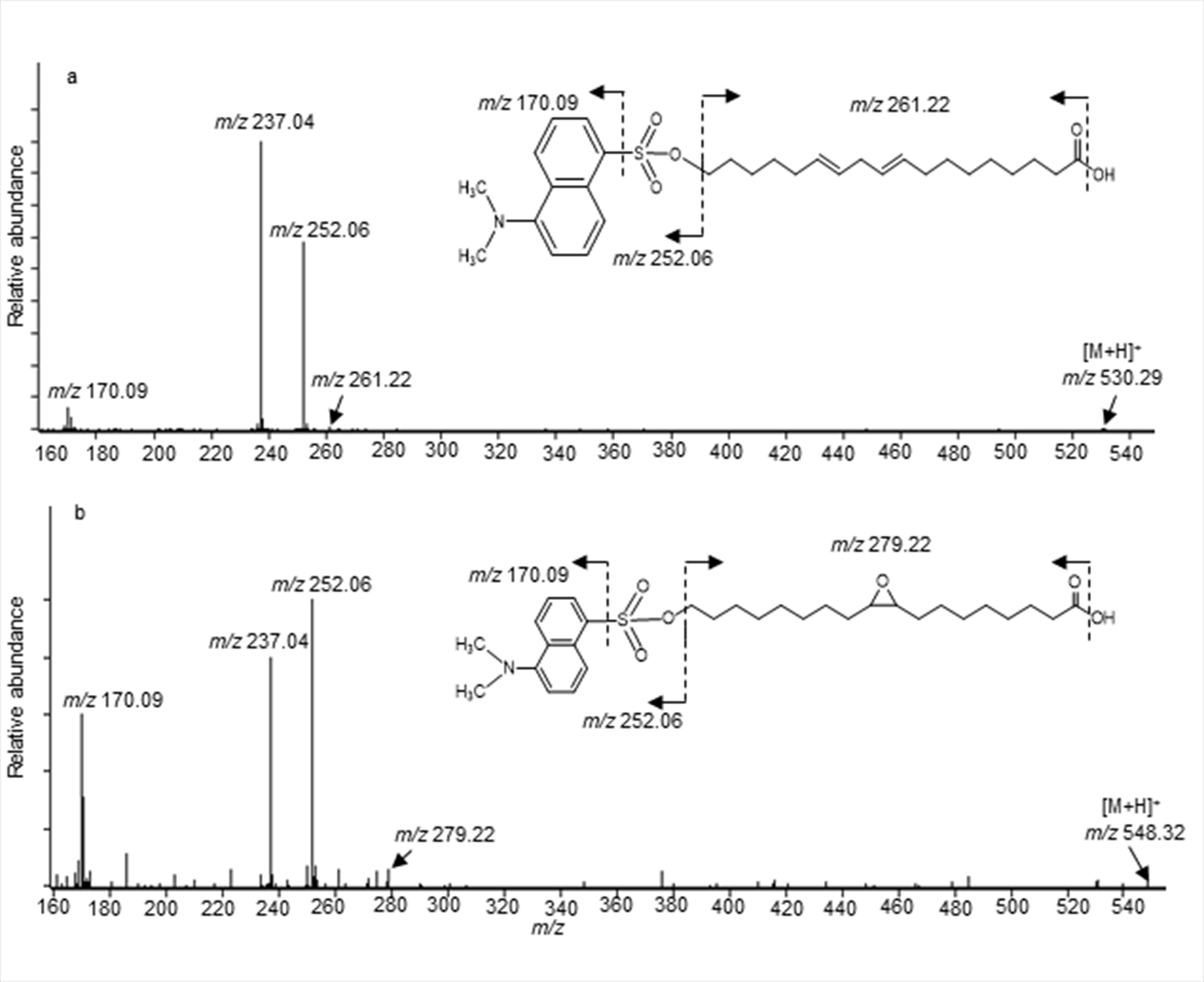

Supplement: Supplementary file 8 — Additional file 8: Fig. S7. LC–MS/MS identification of dansyl-derivatized hydroxy-FAs a) 18-hydroxy-18:2 (m/z 530.29); and b) 18-hydroxy-9,10-epoxy-18:0 (m/z 548.32). [file 13007_2018_384_MOESM8_ESM.tif]

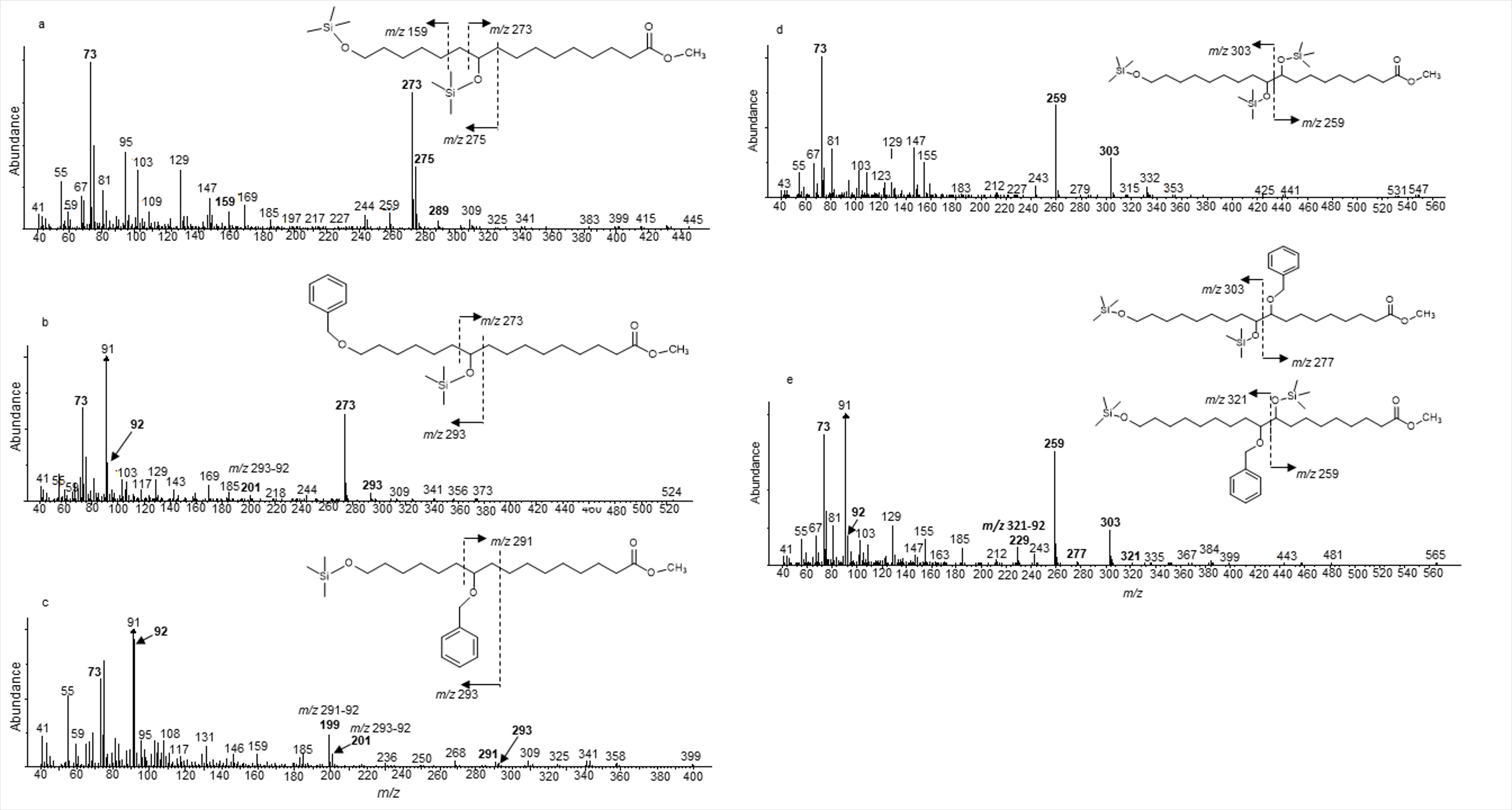

Supplement: Supplementary file 9 — Additional file 9: Fig. S8. GC/MS identification of silylated, benzyl-O-alkylated hydroxy-FA methyl esters: a) non-alkylated 10,16-dihydroxy-16:0; b) ω-alkylated 10,16-dihydroxy-16:0; c) 10-alkylated 10,16-dihydroxy-16:0; d) non-alkylated 9,10,18-trihydroxy-18:0; e) 10- or 9-alkylated 9,10,18-trihydroxy-18:0. [file 13007_2018_384_MOESM9_ESM.tif]

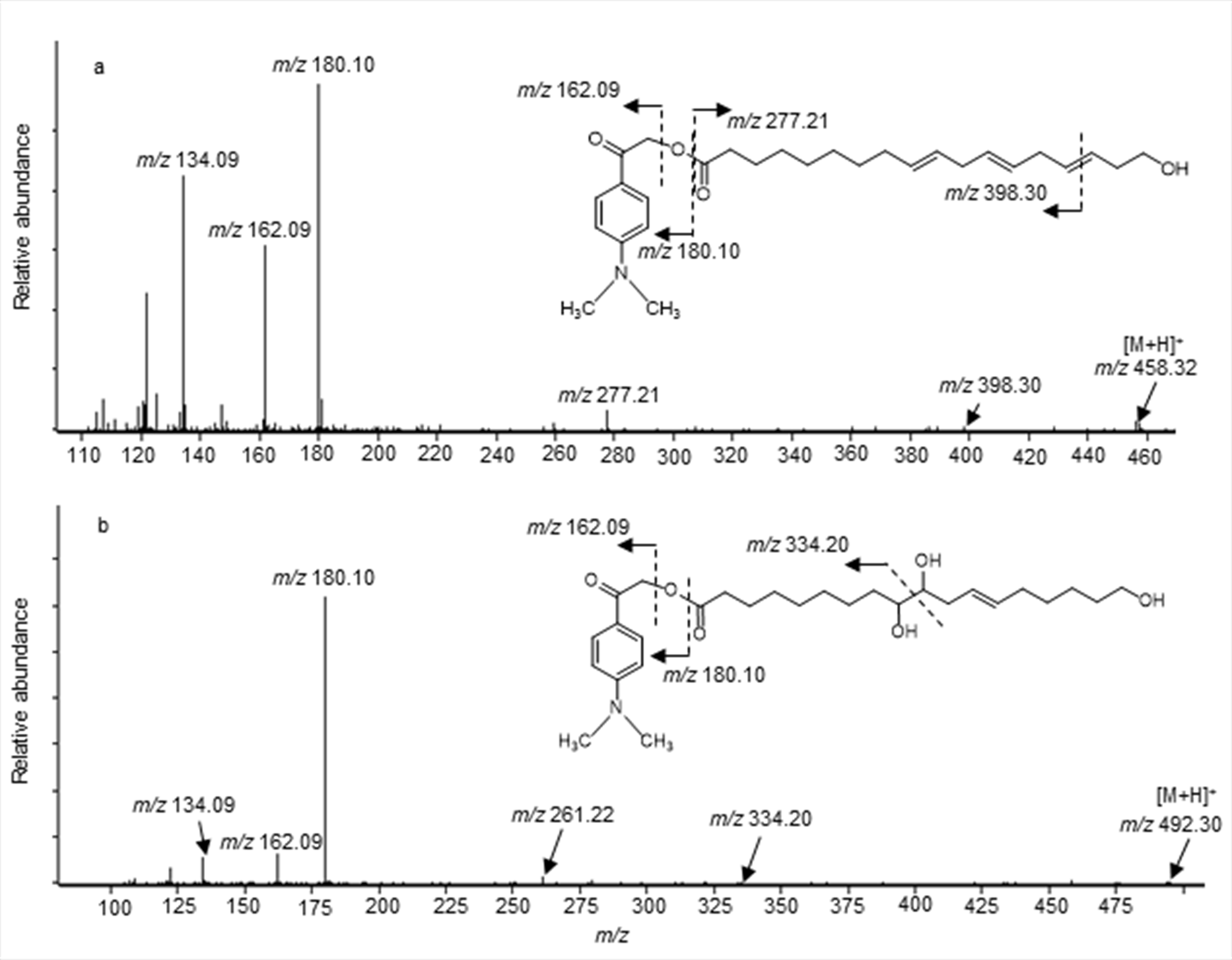

Supplement: Supplementary file 10 — Additional file 10: Fig. S9. LC–MS/MS identification of DmPA-derivatized a) 18-hydroxy-18:3 (m/z 458.32); and b) 9,10,18-trihydroxy-18:0 0 (m/z 492.30). [file 13007_2018_384_MOESM10_ESM.tif]

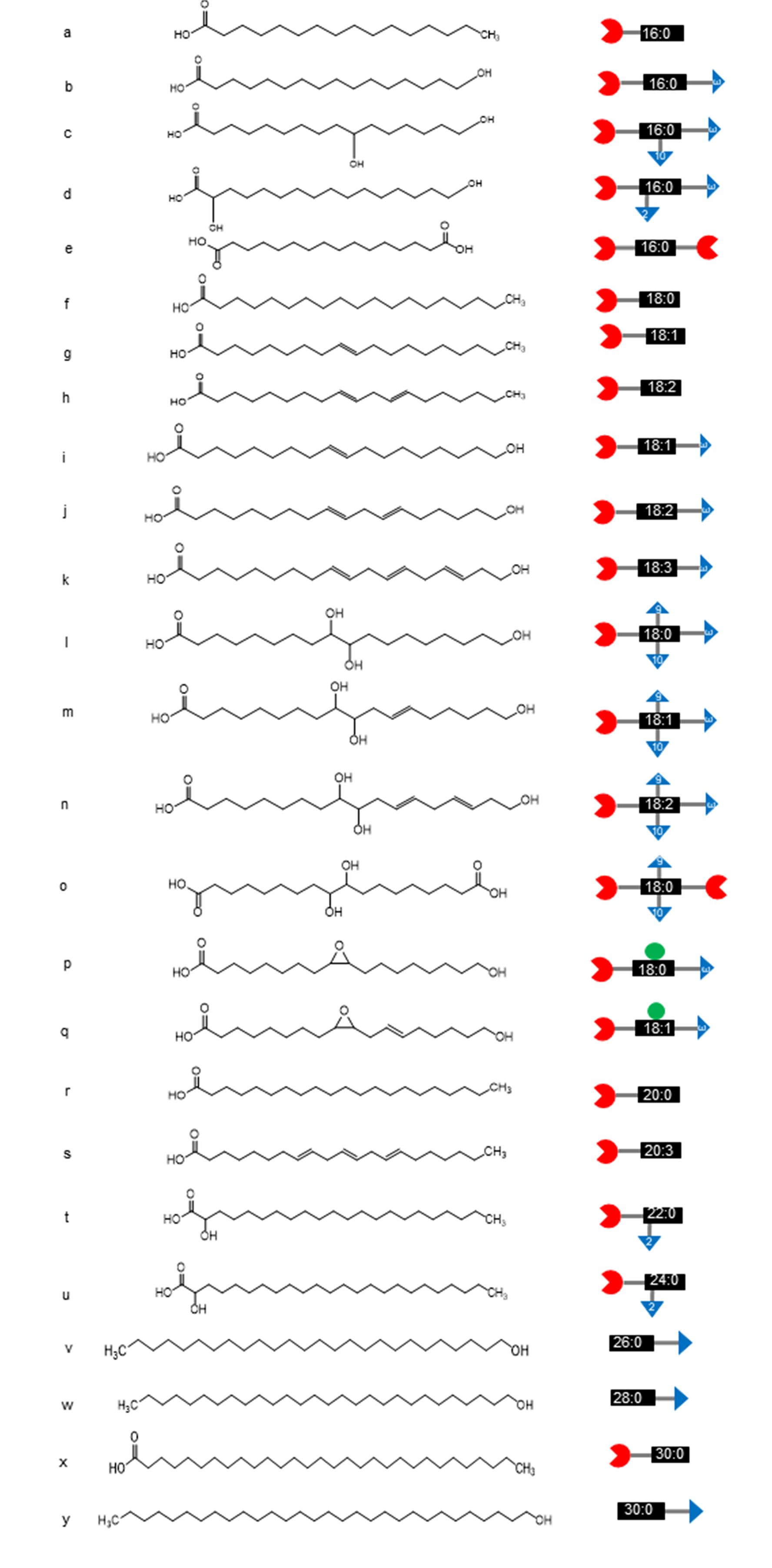

Supplement: Supplementary file 11 — Additional file 11: Fig. S10. Schematic representation of cutin monomers. The digits within the black-filled rectangle represent the nature of the acyl moiety in the standard short-hand fatty acid nomenclature. The red-shaded “PAC-MAN” symbol represents the carboxyl-group of each acyl-chain. The digits in the blue-shaded triangles represent the positions of hydroxyl groups on the acyl-chain, and the digits in the green-shaded circles represent the positions of epoxy-groups on the acyl-chain. The blue-shaded triangle juxtaposed in the red PAC-MAN schematic represents the ester bond between adjoining acyl-monomers. a) Hexadecanoic acid (palmitic acid). b) 16-Hydroxyhexadecanoic acid. c) 10,16-Dihydroxyhexadecanoic acid. d) 2-Hydroxyhexadecanoic acid. e) Hexadecanedioic acid. f) Octadecanoic acid (stearic acid). g) 9-Octadecenoic acid (oleic acid). h) 9,12-octadecadienoic acid (linoleic acid). i) 18-hydroxy-9-octadecenoic acid. j) 18-hydroxy-9,12-octadecenoic acid. k) 18-hydroxy-9,12,15-octadecenoic acid. l) 9,10,18-trihydroxyoctadecanoic acid. m) 9,10,18-trihydroxyoctadec-12-enoic acid. n) 9,10,18-trihydroxyoctadec-12,15-dienoic acid. o) 9,10-dihydroxy-octadecanedioic acid. p) 18-hydroxy-9,10-epoxoyctadecanoic acid. q) 18-hydroxy-9,10-epoxyoctadeca-12-enoic acid. r) Eicosanoic acid. s) 8,11,14-eicosatrienoic acid. t) 2-hydroxydocosanoic acid. u) 2-hydroxytetracosanoic acid. v) 1-hexacosanol. w) 1-octacosanol. x) Triacontanoic acid. y) 1-triacontanol. [file 13007_2018_384_MOESM11_ESM.tif]

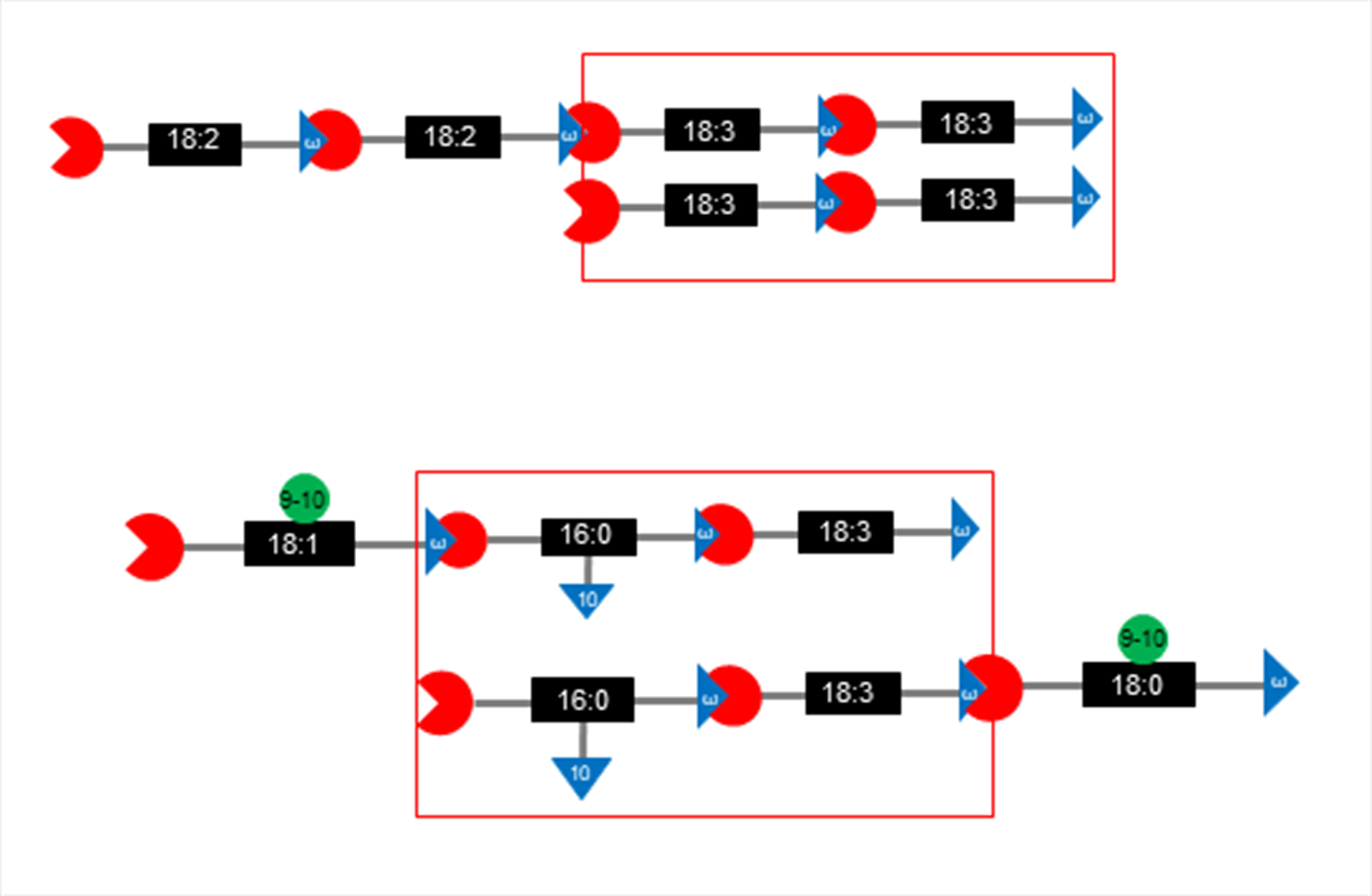

Supplement: Supplementary file 12 — Additional file 12: Fig. S11. Schematic representation of overlapping region of cutin subfragments. The red-box identifies monomer overlaps among different subfragments that may indicate they are adjoining in the cutin polymer. [file 13007_2018_384_MOESM12_ESM.tif]
